# Selective, Intrinsically Fluorescent Trk Modulating Probes

**DOI:** 10.1021/acschemneuro.4c00290

**Published:** 2024-10-02

**Authors:** Thitima Pewklang, Tye Thompson, Arthur Sefiani, Cédric G. Geoffroy, Anyanee Kamkaew, Kevin Burgess

**Affiliations:** † Department of Chemistry, Texas A & M University, Box 30012, College Station, Texas 77842-3012, United States; ‡ School of Chemistry, Institute of Science, Suranaree University of Technology, Nakhon Ratchasima 30000, Thailand; § Department of Neuroscience and Experimental Therapeutics, Texas A & M University Health Science Center, Bryan, Texas 77807, United States; ∥ NeuroCreis, Inc., College Station, Texas 77840, United States

**Keywords:** Tropomysin, neurotrophin, Trk, fluorescent probe, peptidomimetic, BODIPY, *cyclo*-organopeptides

## Abstract

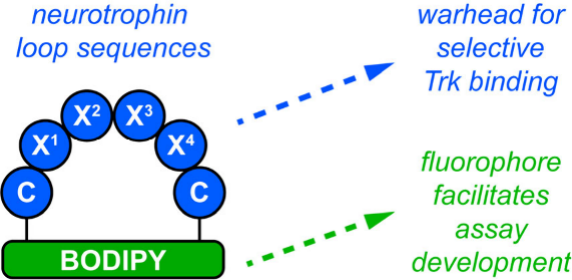

Neurotrophins (NTs) elicit the growth, survival, and differentiation of neurons and other neuroectoderm tissues via activation of Trk receptors. Hot spots for NT·Trk interactions involve three neurotrophin loops. Mimicry of these using “*cyclo*-organopeptides” comprising loop sequences cyclized onto endocyclic organic fragments accounts for a few of the low molecular mass Trk agonists or modulators reported so far; the majority are nonpeptidic small molecules accessed without molecular design and identified in random screens. It has proven difficult to verify activities induced by low molecular mass substances are due to Trk activation (rather than via other receptors), enhanced Trk expression, enhanced NT expression, or other pathways. Consequently, identification of selective probes for the various Trk receptors (e.g., A, B, and C) has been very challenging. Further, a key feature of probes for early stage assays is that they should be easily detectable, and none of the compounds reported to date are. In this work, we designed novel *cyclo*-organopeptide derivatives where the organic fragment is a BODIPY fluor and found ones that selectively, though not specifically, activate TrkA, B, or C. One of the assays used to reach this conclusion (binding to live Trk-expressing cells) relied on intrinsic fluorescence in the tested materials. Consequently, this work established low molecular mass Trk-selective probes exhibiting neuroprotective effects.

## Introduction

Neurotrophins (NTs) activate Trk receptors (a subset of receptor tyrosine kinases, RTKs) to regulate growth, survival, and differentiation of neurons and other neuroectoderm tissues in which they are expressed.^1^ Trk receptors are selective (NGF for TrkA; BDNF and NT-4 for B; and NT-3 for C) but not specific.^2^ For instance, NT-3 binds TrkA and B, but with lower affinity than for C.^3,4^ All NTs also bind the “death receptor” p75 which can promote apoptosis and survival or otherwise regulate Trk activities.^5−11^ Expression of p75 can also determine whether NT-3 binds and activates TrkA.^12−14^ Stimulation of NT·Trk combinations at the cell surface induces different signaling pathway effects. For instance, NGF·TrkA stimulates phosphorylation of ERK1 and JNK1, epithelial colony formation, *and* proliferation, but BDNF·TrkB only enhances colony formation.^15,16^ Ultimately, these signaling pathways result in different neural growth and differentiation outcomes.^17^

Neurotrophins are attractive therapeutic targets,^18^ but the native proteins are not viable for most disease states. Their blood half-lives are on the order of minutes,^19^ and side-effects in clinical trials include neuropathy.^20,21^ One exception is humanized NGF (Cenegermin) for treatment of neurotrophic keratitis (in the eye).^22^ However, this drug is extremely expensive (recombinant NGF is difficult to make reproducibly and has a limited shelf life), and requires frequent, prolonged administration. In any case, delivery into the eye is a special case which circumvents some of the pharmacokinetic (PK) obstacles to using NTs for other neurodegenerative diseases, notably the blood–brain and blood spinal cord barriers which mediate permeation into the brain^19,23^ and spinal cord, respectively.^24^ Further, gene therapy strategies to express neurotrophic factors (NTFs) for nerve repair in the peripheral nervous system can result in uncontrolled axon growth and hypersensitivity.^25^ Consequently, discovery of small molecule Trk agonists^20,26^ is a particularly appealing alternative.

Small molecule Trk agonists have different PK profiles from the parent NTs, most significantly half-lives *in vivo* and permeation into the brain and spinal cord. They also tend to have more favorable shelf-lives, production costs, and batch-to-batch reproducibility.^27^

**Figure 1:**
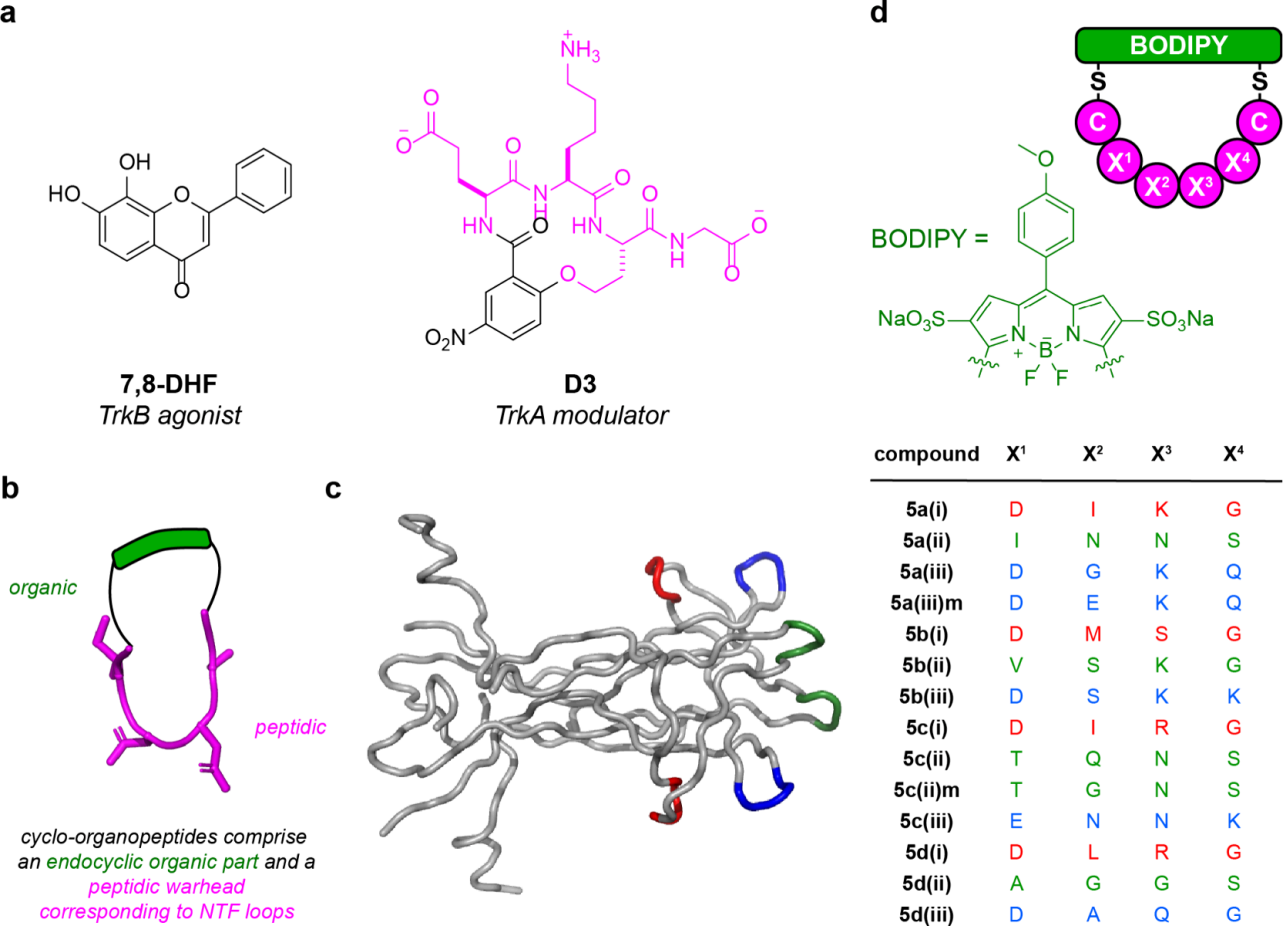
(a) Examples of other previously reported Trk modulators **7,8-DHF** and **D3**. (b) General strategy depicting the construction of *cyclo*-organopeptides for this work. (c) Crystal structure (PDB 1WWW) of NGF indicating the three mimicked loops (loop (i), red; (ii), green; and (iii), blue). (d) *This work:* cyclo-organopeptides comprising a BODIPY organic fragment cyclized to a CX^1^X^2^X^3^X^4^C-peptide. Warhead sequences of the four central amino acids correspond to each neurotrophin loop.

Various completely nonpeptidic small molecule Trk agonists have been reported,^26^ several of which were identified from biological screening of large libraries. 7,8-Dihydroxyflavone^28^ (7,8-DHF, Figure 1a) has been studied extensively as a TrkB agonist,^29^ though molecular rationales for why these types of compounds bind and activate Trk can be difficult to conceive, necessitating extra vigilance for receptor target validation and selectivity. This compound was recently reported to bind to a total of 133 intramolecular targets, highlighting its lack of selectivity,^30^ and there are several reports of alternative mechanisms of action.^31,32^ There have been numerous publications describing difficulties in reproducing the reported effects of several other small-molecule Trk agonists,^30,33,34^ including ones designed by mimicking neurotrophin loop structures.^35^ Establishing selectivity between TrkA–C can also be problematic, and this is accentuated by issues with some cell assays, as now outlined.

Rigorous binding studies require radiolabeling, but this is frequently avoided due to synthesis difficulties, safety concerns, and experimental inconvenience. Reliabilities of Western blots depend on antibody qualities, and commercial antibodies for phosphorylated Trks (pTrks) tend to be poor.^36^ Consequently, blots reported for small molecule derivatives interacting with Trks tend to be inconclusive. Indeed, data based on pTrkB has been questioned in a thorough study of putative agonists by Sames and co-workers,^36^ and others have reported similar concerns.^33,37,38^ Sames’ work showed an ELFI (enzyme-linked fixed-cell immunoassay) can be a useful alternative to blotting because it detects downstream points for phosphorylative signaling (e.g., MAPK and AKT) via widely used and well-validated antibodies. Part of our studies reported here confirm and elaborate on the limitations of blotting assays in this field.

*cyclo*-Organopeptides (Figure 1b) comprising a peptidic fragment ring-closed via an endocyclic organic (i.e., nonpeptidic) part can be NT mimics. NT β-turns influence Trk-selectivity (NGF to A, BDNF and NT-4 to B, and NT-3 to C). Crystallographic evidence indicates NGF buries its three β-turns (per monomer, Figure 1c) into the linker region between the extracellular domain and the transmembrane domain of TrkA.^39,40^ Studies featuring NT point mutations and chimeras confirm these turn regions are hot-loop^41−44^ binding and selectivity determinants.^45−52^ Throughout this paper, loops (i)–(iii) are color coded red, green, and blue, respectively, as in Figure 1c.

In 1998, we designed and reported^53^ a *cyclo*-organopeptide **D3** (Figure 1a) which mimics one of the β-turns in NGF.^54^**D3** is an NGF potentiator through TrkA (meaning it enhances the activity of NGF) and does not bind TrkC or p75.^55^ We used the same strategy to prepare similar NT loop mimics,^53,54,56,57^ hence generating TrkC modulators (modulator refers to a molecule which can affect Trk by some mechanism other than direct agonism, typically by increasing the affinity of the native neurotrophin for the receptor).^58−65^ Since then, **D3** (Tavilermide) reached phase 3 trials for treatment of dry eye disease, and the Burgess lab has reported^66^ and patented^67^ other hot loop mimics that are *superior in vivo* for this malady; others have also prepared *cyclo*-organopeptide loop mimics of NTs.^48,49,51,68−72^

Based on these observations, we assert *cyclo*-organopeptide β-turn mimics of NTs are *privileged* chemotypes for Trk agonism; i.e., they give hits in Trk assays at ∼100–10,000× the rate of those in random screens.

Recently, members of our team reported new chemistry to make *cyclo*-organopeptide loop mimics involving a novel CLIPS (Chemical Linkage of Peptides onto Scaffolds)^73−78^ reaction. Specifically, a peptide *warhead* sequence (e.g., corresponding to one of the hot loops in BDNF) flanked by two Cys (or similar) residues was cyclized onto a sulfonated, dichloro-BODIPY dye^79^ via two S_N_Ar reactions^80^ giving structures generalized in Figure 1c. These reactions were performed on unprotected peptides in aqueous buffer and efficiently gave *cyclo*-organopeptides with high conversions, facilitating convenient purifications via preparative HPLC.

Conceptually, *cyclo*-organopeptides deliberately containing an endocyclic fluorophore for screening are new (see ref (81) for the most closely related work). This strategy has “up-front” advantages insofar as fluorescence can be used to quantitate binding to live cells selectively expressing targeted receptors (in parent cell lines that do not naturally), without radiolabeling or compound modification. Intracellular permeability and localization are also easy to observe for intrinsically fluorescent loop mimics. Conversely, clinical candidates having unnecessary fluorescence are unusual; therefore, substitutions may sometimes be necessary. Overall, in some situations, it may be advantageous to make discovery easier by using intrinsically fluorescent loop mimics, even if downstream structural changes are inevitable after.

Here, we report a study of 14 *fluorescent cyclo*-organopeptides based on Trk (Figure 1d, where **5** delineates the fifth series prepared in this lab, **a**–**d** means NGF, BDNF, NT-3, and NT-4 mimic, respectively, and **(i)**–**(iii)** corresponds to those loops shown in Figure 1b). We also identified some of these that bind TrkA, B, and C selectively. One, **5c(ii)**, was tested in primary adult cortical neurons (extracted *post-mortem* from six-week-old male mice) in neurite outgrowth and a cell survival assay as a screen for neuroprotective effects.

## Results and Discussion

### Cell Survival Assays

#### Approach

**Figure 2:**
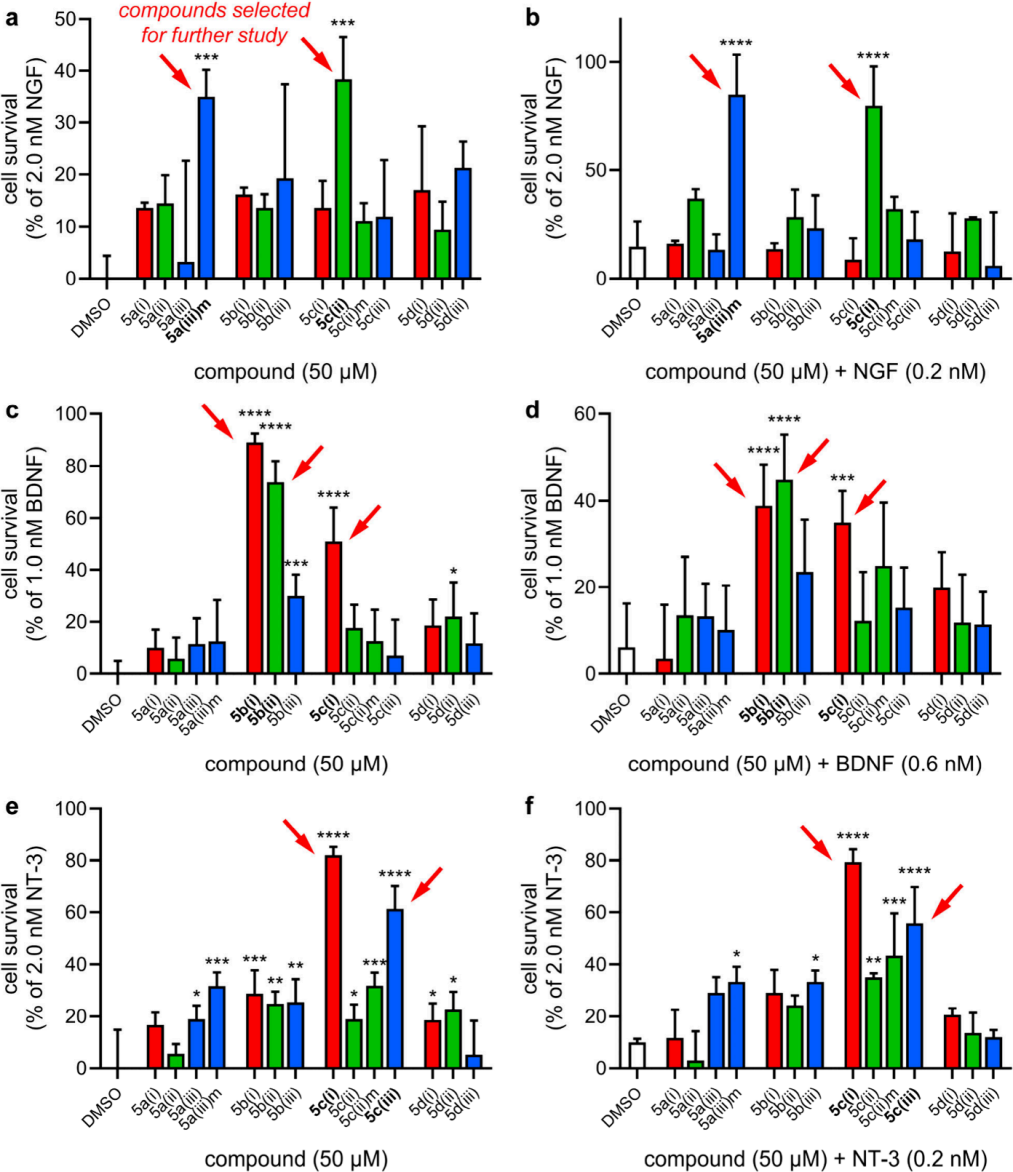
Mimic-induced cell survival of TrkA-expressing cells (a and b), B-expressing cells (c and d), or C-expressing cells (e and f) in the absence of NT (“**-NT**”, a, c, e) or in the presence of suboptimal NT (“**+NT**”, b, d, f). Cells were incubated in serum-free media with compound and/or neurotrophin for 48–72 h; then, viability was assessed via flow cytometry. Data is represented as mean ± SD where *n* = 3. Data was analyzed via one-way ANOVA followed by Dunnett’s *t* test compared to the DMSO control where *p < 0.05, **p < 0.01, ***p < 0.001, and ****p < 0.0001.

**Figure 3:**
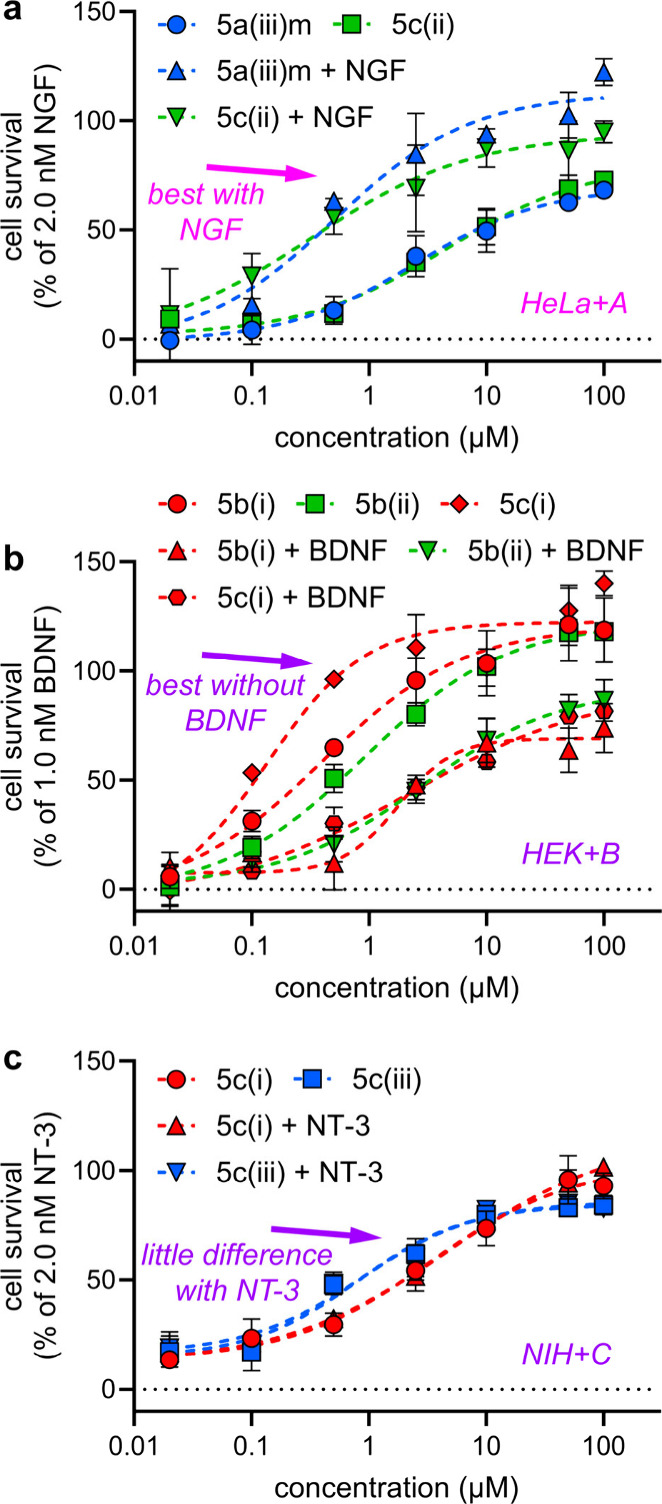
(a–c) Concentration-dependent cell survival (both **-NT** and **+NT**) for top-performing compounds in TrkA-, B-, and C-expressing cells, respectively. Cells were incubated in serum-free media with compound and/or neurotrophin for 48–72 h; then, viability was assessed via flow cytometry. Data points for **-NT** are circles, squares, and diamonds; those for **+NT** are triangles, inverted triangles, and hexagons. Data is represented as mean ± SD where *n* = 3. Curves were fit and EC_50_ calculated using the nonlinear regression “{agonist} *vs* response – Variable slope (four parameters)” analysis in GraphPad Prism 10.2.

Loop mimics **5** were screened in cell survival assays to evaluate their efficacy in stimulating Trk receptors (Figure 2). Mimics were assayed for the ability to rescue cells from cell death when incubated in serum-free media (SFM) in the absence of NTs (a test for “agonism”) or in the presence of a suboptimal NT concentration (a test for NT “modulation”). Simultaneously, these assays may confirm test mimics are not cytotoxic at lower doses (though cytotoxicity assays were also performed up to higher doses, Figure S1). Throughout, the data are normalized relative to maximum survival imparted by NT (determined by experiment to be 2.0 nM NGF; 1.0 nM BDNF; and 2.0 nM NT-3). All the test compounds were screened at 50 μM concentrations initially, and then, dose response curves were measured for the hits (Table 1 and Figure 3). The data showed sigmoidal relationships in the concentration range tested.

**Table 1:** Efficacy in Dose-Dependent Cell Survival and Cell-Surface Binding^a^

receptor	compound	EC_50_ (μM)	EC_50_ (μM) with suboptimal neurotrophin	*K*_d_ (nM) for cell surface
TrkA	**5a(iii)m**	2.4	0.5	120 ± 3
	**5c(ii)**	4.5	0.3	91 ± 2
TrkB	**5b(i)**	0.4	1.8	73 ± 13
	**5b(ii)**	0.9	2.8	115 ± 66
	**5c(i)**	0.1	2.2	43 ± 2
TrkC	**5c(i)**	3.3	4.9	97 ± 4
	**5c(iii)**	0.8	0.9	91 ± 4

a Suboptimal NT concentrations were 0.2 nM NGF, 0.6 nM BDNF, and 0.2 nM NT-3 for TrkA-, B-, and C-expressing cell lines, respectively. Survival data was normalized relative to DMSO (0% survival) and optimal neurotrophin (2.0 nM NGF, 1.0 nM BDNF, 2.0 nM NT-3; 100% survival). EC_50_ values were calculated using the nonlinear regression “{agonist} *vs* response – Variable slope (four parameters)” analysis, and K_d_’s were calculated by first subtracting nonspecific binding, then using the nonlinear regression “One site – specific binding” analysis in GraphPad Prism 10.2.

We were forced to use transfectants in different cell lines (HeLa, HEK-293, NIH-3T3 expressing TrkA, B, and C, respectively) due to difficulties in obtaining transfected cells derived from a single wild type; however, none of the cells used express TrkA-C prior to transfection. Throughout this paper, HeLa cells are denoted as “HeLa”, and their TrkA transfectants are “HeLa+A”. Similarly, HEK-293 cells are “HEK”; their TrkB transfectants are HEK+B. NIH-3T3 cells are “NIH”, and their TrkC transfectants are “NIH+C”.

#### Tests for Activities Mediated via TrkA

**5a(iii)m** (**m** stands for mouse loop sequence, human sequences are not indicated throughout) and **5c(ii)** at 50 μM induced the highest levels of cell survival, without NGF (35 ± 5% and 38 ± 8%, respectively, Figure 2a) and in the presence of 0.2 nM NGF (85 ± 18% and 80 ± 18%, respectively, Figure 2b). In dose dependence experiments, TrkA-selective loop mimics **5a(iii)m** and **5c(ii)** had lower EC_50_’s and higher maximal activity with suboptimal NGF (0.5 and 0.3 μM, respectively) compared to that without (2.4 and 4.5 μM; Figure 3a, Table 1). In summary, TrkA leads **5a(iii)m** and **5c(ii)** gave more cell survival in the **+NT** experiments designed to detect modulation.

#### Activities Mediated via TrkB

In assays with TrkB-expressing cells, **5b(i)**, **5b(ii)**, and **5c(i)** induced cell survival without supplemental BDNF (89 ± 3%, 74 ± 8%, 51 ± 13%, respectively, Figure 2c). When TrkB-expressing cells were incubated with loop mimics *and* 0.6 nM BDNF, the efficacy was less: 39 ± 9%, 45 ± 10%, and 35 ± 7% cell survival, Figure 2d. These data are indicative of competitive TrkB activation by the loop mimics and BDNF. All three loop mimics exhibited dose-dependently induced survival of TrkB-expressing cells. When incubated with 0.6 nM BDNF, they reached their activity ceilings at *higher* concentrations (higher EC_50_’s) than without the NT (Figure 3b, Table 1). In other words, they appear to be agonists of TrkB and are *less* effective with suboptimal NT. Thus, **5b(i)**, **5b(ii)**, and **5c(i)** gave more cell survival in the **-NT** experiments designed to detect agonism, in contrast to data described above for TrkA activation with other mimics.

#### TrkC

In the initial screens, **5c(i)** induced 82 ± 3% cell survival, and **5c(iii)** induced 61 ± 9% survival in TrkC-expressing cells in the absence of NT-3, Figure 2e. With 0.2 nM NT-3, survivals induced by the loop mimics were slightly less (79 ± 5% and 56 ± 14%, respectively; Figure 2f). **5c(i)** and **5c(iii)** also induced dose-dependent cell survival of TrkC-expressing cells without NT-3. However, in contrast to the TrkA and TrkB effectors above, incubation with suboptimal levels of neurotrophin (0.2 nM NT-3) results in little difference in EC_50_ or maximum compound activity (Figure 3c, Table 1); i.e., for **5c(i)** and **5c(iii)** the **-NT** and **+NT** data are similar.

#### Lead Selection from Cell Survival

**5a(iii)m** and **5c(ii)** were selected for TrkA (**+NT**); **5b(i)**, **5b(ii)**, and **5c(i)** were selected for B (**-NT**), and **5c(i)** and **5c(iii)** were selected for C (**-/+NT**). Thus, all mimics selected for activation of TrkA–C, respectively, were different, except **5c(i)** which was selected for TrkB and C. Loop correspondences for the best TrkA activators **5a(iii)m** and **5c(ii)** were primarily for (iii) and (ii), though there is some overlap between sequences (discussed in the Conclusions, Figure 8). For the TrkB hits, the loop correspondences were **5b(i)**, **5b(ii)**, and **5c(i)**, i.e., (i) twice and (ii); and for C, **5c(i)** and **5c(iii)** have neurotrophin loop (i) and (iii) sequences. To find active compounds, these limited data imply no preference for mimicry of any particular loop. From this point on, we considered only the six mimics selected above.

We then tried to establish convenient secondary confirmatory assays. Ultimately, data showed that ELFI assays had little value, and we suggest why. However, first we discuss binding data featuring live transfectant cells; this was useful and demonstrates one of the advantages of focusing early work on intrinsically fluorescent loop mimics.

### Binding Assays

Binding of the selective, intrinsically fluorescent mimics to cells can be observed and quantitated via fluorescence. Raw data includes fluorescence associated with material not removed in the washing step (sticky, nonselective binding), and if there were binding to any receptors other than Trk, then fluorescence would be observed for this too. In a second experiment, fluorescence associated with the wild-type cells *without Trk expression* was similarly recorded to provide a baseline correction representative of that nonselective binding, plus affinity to non-Trk receptors if that occurred. Consequently, fluorescence observed in the noncompetitive experiment minus that in the one using Trk-negative cell lines represents a minimum associated with binding to the expressed Trk receptor. Overall, this procedure is based on one by Low and co-workers^82^ featuring fluorescently *labeled* small molecule ligands (for receptors other than Trks) and a competitive control. Our variant does not require introduction of a fluorescent label because the leads are intrinsically fluorescent. This is a significant advantage because “extrinsic fluorophores” tend to be as large as many small molecule leads and have their own binding characteristics.

Throughout, experiments were also performed in the presence of NTs corresponding to the Trk receptors expressed. It was anticipated that NTs would block some binding of the intrinsically fluorescent leads. If so, that would be indicative of lead/NT competition *for the same Trk receptor binding site*, giving further evidence hits bind transfected Trk receptors.

**Figure 4:**
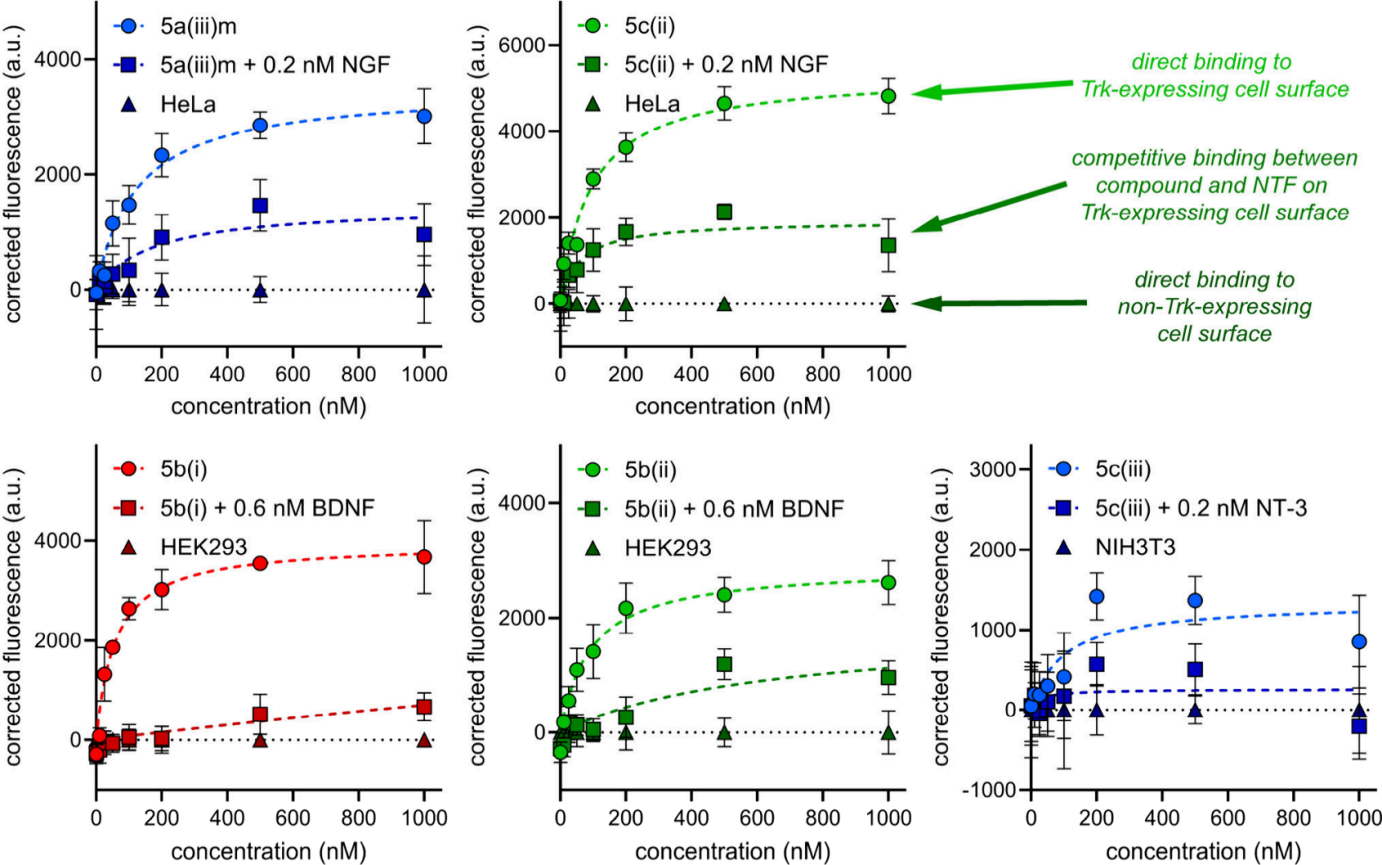
Cell-surface fluorescence binding experiments for **5a(iii)m** to HeLa+A; **5c(ii)** to HeLa+A; **5b(i)** to HEK293+B; **5b(ii)** to HEK293+B; **5c(iii)** to NIH3T3+C. Data are represented as mean ± SD where *n* = 3 and are representative of three independent experiments. Corrected fluorescence means were obtained by subtracting the background fluorescence of compound binding to the wild-type cell line and SD by propagation of error. K_d_’s were calculated using the nonlinear regression “One site – specific binding” analysis in GraphPad Prism 10.2.

**Figure 5:**
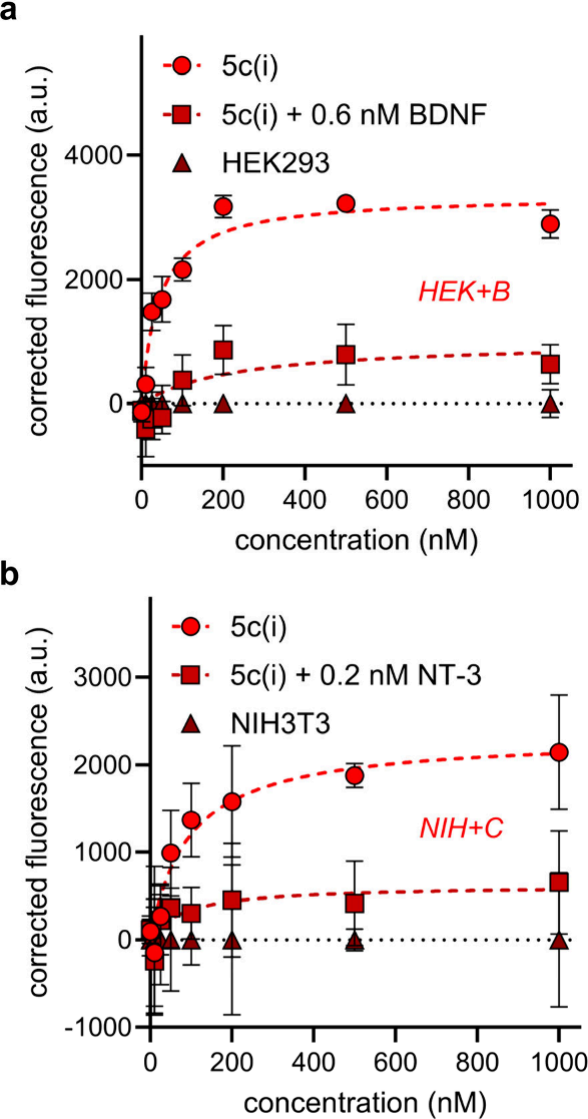
Cell-surface fluorescence binding experiments for **5c(i)** to (a) HEK-TrkB and (b) NIH-TrkC. Data are represented as mean ± SD where *n* = 3 and are representative of three independent experiments. Corrected fluorescence means were obtained by subtracting the background fluorescence of compound binding to the wild-type cell line and SD by propagation of error. K_d_’s were calculated using the nonlinear regression “One site – specific binding” analysis in GraphPad Prism 10.2.

Data for binding of the lead compounds to Trk transfectants are shown in Figure 4 and 5 where red, green, and blue colors follow the Figure 1c convention to indicate loop sequences the peptide warheads are based upon. Throughout Figure 4, data points for key experiments are circles, squares represent the lead/NT competitions, and triangles are data for binding to the wild-type cell lines. Data for **5c(i)** is shown separately in Figure 5 for clarity since this compound binds both TrkB and C transfectants.

The following generalities apply to Figures 4 and 5. First, more binding occurred to the transfectants (circular data points) than to the parent cell lines (triangles), as expected for Trk binders. Second, loop mimic binding was reduced in the lead/NT competition experiments, confirming the lead compounds bind at the NT binding sites. Third, *K*_d_ values deduced for their binding TrkA–C were within the range 43–120 nM (Table 1, far right column).

**5c(i)** was the highest affinity Trk binder to TrkB-expressing cells (43 nM), and it also bound TrkC-expressing cells with midrange affinity (97 nM). For cross-assay comparisons, note that Figure 2 showed **5c(i)** caused more cell survival than any other lead for the TrkB and C transfectants (Table 1). Based on those data combined, **5c(i)***is a selective agonist of TrkB and C*.

Another loop mimic, **5c(ii)**, which was exceptional in the ELFI assay outlined below, bound TrkA-expressing cells with a affinity of 91 nM affinity. Figure 2 shows **5c(ii)** was one of the two most effective survival inducers for TrkA transfectants in both **-** and **+NT** experiments, but it was unremarkable with respect to induced cell survival of transfectants bearing TrkB and C. Based on this data combined, **5c(ii)***appears to be a TrkA selective modulator*.

Our first paper in this series^81^ featured experiments with **5b(i)** and **5b(ii)** but only on TrkB transfectants. The studies here now show these mimics have TrkB *selectivity* in cell survival assays (Figure 2c,d, compared with a, b, e, and f). Further, they bound the TrkB-expressing cells with 73 and 115 nM K_d_’s (Table 1).

### Cell Signaling

The introduction to this paper describes literature evidence that anti-phosphoTrk antibodies are poor and give unreliable Western blotting data. We labored over Western blot experiments and ultimately reached the same conclusion. Besides, three other limitations became apparent: (i) small molecules tend to perturb Trk on shorter time scales than the NTs, which we now know give maximal response with 1–2 h of cell treatment in our methodology, so experimentation is required to optimize these times to detect their effects; (ii) pTrkA–C antibodies are required; these vary in quality so much that comparisons between receptor responses becomes uncertain; and (iii) Western blotting procedures, especially for dose response studies, have poor throughput.

For the reasons outlined above, we switched to ELFI assays. ELFI for Trk focuses on the same signaling restriction points, here pMAPK and pAKT, using reliable antibodies, and data can be compared for activation of the different Trks. Further, throughput for ELFI assays is probably 2–10× that for Western blots. Consequently, we expended significant effort to validate ELFI for the detection of small differences in signaling through MAPK and AKT with an acceptable degree of confidence. Procedures now described were used to do this.

**Figure 6:**
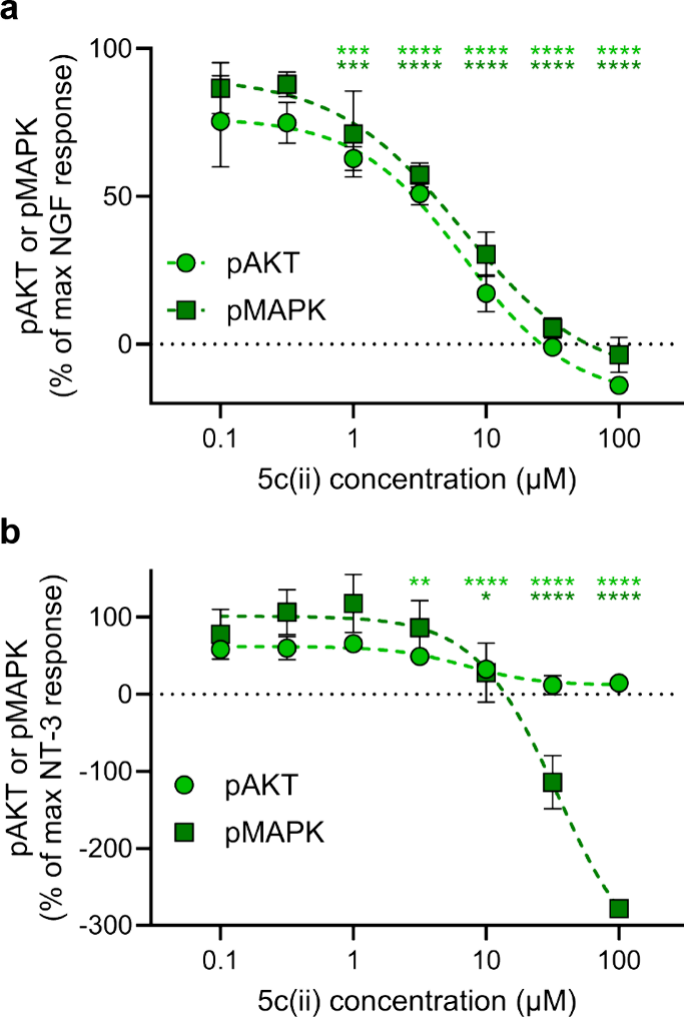
ELFI for **5c(ii)** competing against NT (10 ng/mL) for downstream signaling in (a) PC12 cells and (b) NIH+C. Data are represented as mean ± SD where *n* = 3 and are representative of three independent experiments. Data is analyzed via two-way ANOVA followed by Dunnett’s *t* test compared to 10 ng/mL NT control. Significance is denoted as *p < 0.05, **p < 0.01, ***p < 0.001, and ****p < 0.0001.

Throughout, the positive control was concentration-dependent treatment with the parent NT in half-log dilutions from 100 ng/mL to 0.1 ng/mL, and the negative control was to treat the cells with only DMSO. We first determined Z′ factors^83^ based on these boundary controls. Once the assays had been validated (Z′ > 0.5), we determined optimal times to fix cells after treatment (taking into account potential differences in the kinetics of Trk activation between small molecules and neurotrophins), mimicked dose ranges, and then performed dose response studies. For TrkA activation, no statistically significant signaling was observed for HeLa+A cells treated with NGF, so wild-type rat brain PC12 (*TrkA+*) cells were tested instead. Figure 6 shows data normalized to the NT controls (100% response, typically 10 ng/mL NT) and the solvent blank (0%).

Unfortunately, testing most of the lead compounds in ELFI without added NT showed no detectable agonistic response in any case. In actuality, there may have been low levels of agonistic responses below the limits of detection of this assay, which could be responsible for activity observed in cell survival assays.

Repetition of these experiments *with* suboptimal NTs (doses as used in the tests for modulators in the cell survival assays of Figure 2) showed no agonism throughout and apparently *antagonistic* responses in a few cases (data not shown). Consequently, the NT doses were increased to 10 ng/mL to explore this behavior further.

Most compounds with these higher doses of NT showed *inconsequential* (minimum not reached at the highest concentrations tested) signaling *decreases* in pAKT and pMAPK (data not shown). **5c(ii)** was the only exception; it showed statistically significant signaling *decreases* when competed with 10 ng/mL NT: IC_50_ values of 7 and 7 μM for pAKT in PC12 and NIH+C cells, and 7 and 35 μM for pMAPK in PC12 and NIH+C cells. Recall, **5c(ii)** elicited significant cell survival only for TrkA transfectants (**-NT** or **+NT**), and that effect was positive not negative (Figure 2). Others have observed putative Trk ligands (small molecules) can bind cell surface receptors and elicit *positive* cellular responses but *negatively* impact cell signaling. While **5c(ii)** was found to display agonistic activity in cell survival assays, inhibition of the downstream Trk effectors would seem to contradict these results; however, this phenomenon is consistent with the expected pharmacodynamic activity of a partial agonist and is further discussed in the Conclusions.

### Effects on Primary Neuronal Cells

**Figure 7:**
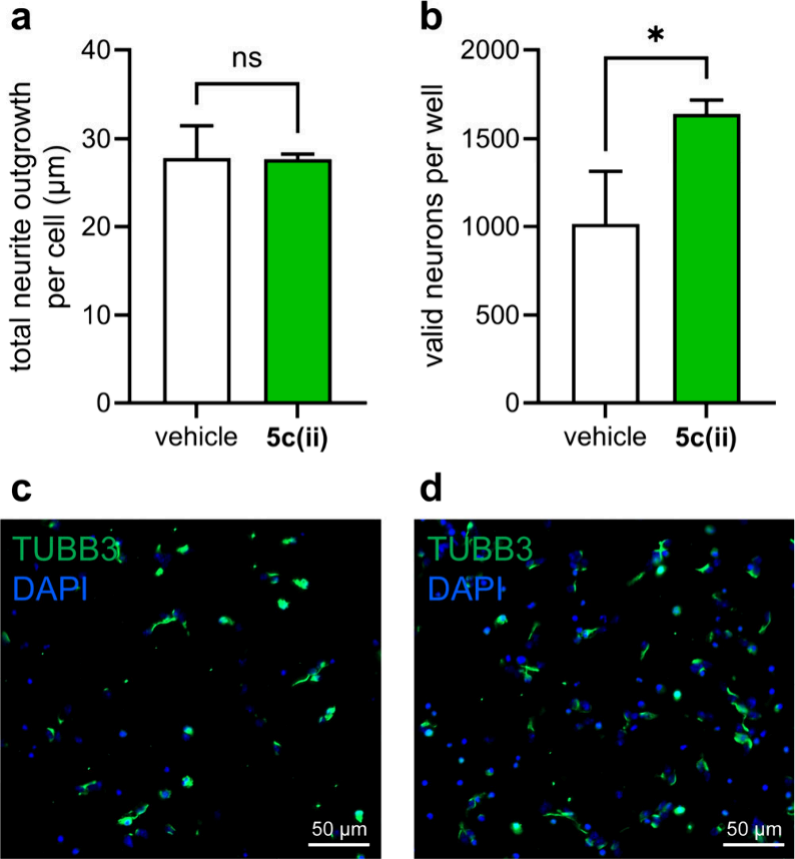
**5c(ii)** promotes neuroprotection. Primary adult cortical neurons were extracted from 6-week-old male mice and plated on 384-well PDL-coated plastic bottom plates at 10,000 cells/well. **5c(ii)** at 5 μM concentration was added at the time of plating. 48 h after plating, the cells were fixed to conduct immunocytochemistry. Graph represents the mean ± SEM of (a) total neurite outgrowth per cell and (b) number of valid neurons per well. Representative images of cortical neurons treated with (c) vehicle and (d) 5 μM **5c(ii)**. Student’s *t* test. *N* = 3–5. *p < 0.05.

We next decided to test the effects of **5c(ii)** on primary mouse adult cortical neurons. These neurons naturally express TrkA^84^ and therefore provide a more clinically relevant substrate to test compound leads in than analogous Trk-expressing transfectants. In addition, using neurons extracted from *adult* mice provide increased relevance to neurodegenerative and other nervous-system related diseases and injury, since most of these primarily occur in adult populations.^85,86^ To determine any potential for preclinical applications, **5c(ii)** (5 μM) was incubated with primary cortical neurons from 6-week-old male mice at the time of plating, and neuronal survival and neurite outgrowth were quantified. While there was no significant change in neurite outgrowth (Figure 7a), **5c(ii)** significantly increased the number of valid neurons after 48 h by over 50% (Figure 7b). The effect of **5c(ii)** is comparable to those of the previously reported and validated compound **RO48** in the same assay.^85^ In contrast, **D3** had no measurable effect in this assay (data not shown).

## Conclusions

This work unambiguously shows that *cyclo*-organopeptides based on NT loop sequences are privileged chemotypes for selective Trk modulation and agonism. Trk *agonism* by small molecules, including *cyclo*-organopeptides, is relatively rare;^87^ many hits in this area, including **D3**, are *modulators*. These studies also demonstrate how intrinsically fluorescent *cyclo*-organopeptides can facilitate direct binding assays on live cells expressing the surface receptors of interest. Radiolabeling test compounds with like-for-like substitutions (e.g., ^3^H for ^1^H or ^14^C for ^12^C) would have involved expensive syntheses with arduous safety precautions.

**Figure 8:**
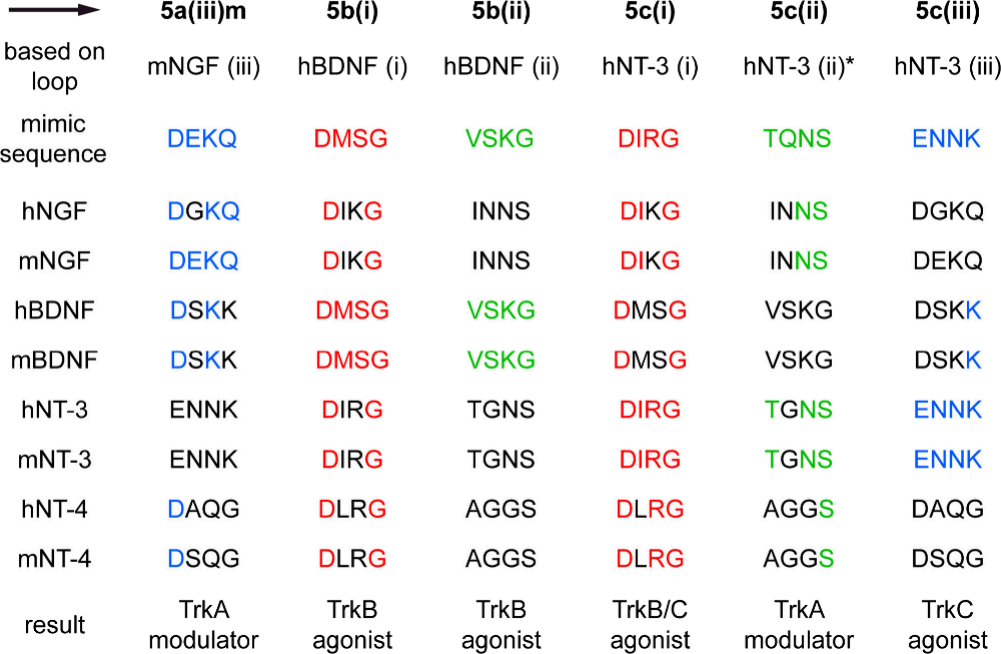
Top three rows indicate selected lead compounds, NTs they were conceived to target, and hence loop sequences incorporated into the warheads. Columns under each mimic then indicate the degree of correspondence to other NTs (m, mouse; h, human): highest correspondences have four colored residues; lowest have none. *5c(ii) was designed to mimic NT-3 with the loop (ii) sequence indicated in (PDB entry 1BND), i.e., TQNS. However, the literature consensus indicates this loop is in fact TGNS (UniProtKB: P20783).

Figure 8 illustrates three mimics designed on loops in TrkA, B, and C (with **a**, **b**, or **c** in their compound labels) affected the Trks targeted (i.e., A, B, and C for **5a(iii)m**, **5b(i)**, **5b(ii)**, **5c(i)**, and **5c(iii)**). In our view, these trends indicate that these loop mimics are privileged chemotypes for selectively targeting Trk receptors with loop sequences corresponding to the mimic warheads. However, deviations were observed for **5c(i)**, which was also an agonist of TrkB, and **5c(ii)** which modulated TrkA, and we proposed the following explanation.

At least two factors may account for TrkB agonism by **5****c****(i)** and modulation of TrkA by **5****c****(ii)**. First, NGF, BDNF, NT-4, and NT-3 are TrkA, B, and C selective and not specific; we already alluded to crosstalk between these ligand–receptor pairs in the introduction. NT-3 is unique in the sense that it binds to all 3 Trk receptors with reasonable affinity.^88^ Second, there is sometimes partial overlap between some of the mimic sequences and NT loops that they were *not* designed to selectively target. Figure 8 illustrates this, where red, green, and blue correspond to NT loops (i)–(iii), respectively, as in Figure 1c. Thus, the sequence of **5c(i)** was designed to be a mimic of the NT-3 loop (i), DIRG, and it is indeed a TrkC agonist. However, it also activates TrkB. **5c(i)** is designed to mimic NT-3 which natively exhibits TrkB-mediated activity. Alternatively, three of the DIRG residues, D-RG, correspond to NT-4 loop (i); either of these observations could explain **5c(i)**’s TrkB activity. A similar correspondence was also observed for **5****c****(ii)** activating TrkA where two of the amino acids encapsulated in **5****c****(ii)**, --NS, also correspond to the NGF loop (ii).

We offer the following explanation for how loop mimics can bind Trk receptors and elicit *positive* cell survival responses but *negatively* impact cell signaling. This is based on one asserted in other pharmacodynamic studies^89^ and by Longo and co-workers to explain why many small molecules exhibit characteristics of Trk agonists in some cases and antagonists in others.^90^

**Figure 9:**
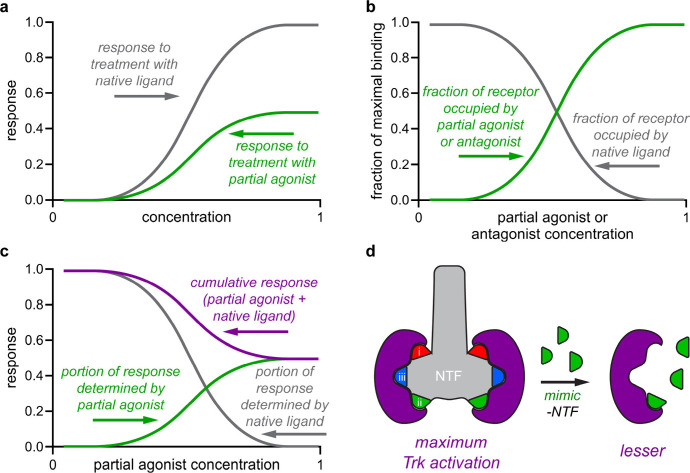
(a) Response to the native ligand (higher response) and partial agonist (lower). (b) Mimics in the presence of fixed NT become the prevalent receptor binder at high concentrations. (c) Increased mimic binding decreases responses by displacing the more potent NT ligands. (d) Elevated loop mimics concentrations displace NT and stimulate the Trks less effectively.

Figure 9a depicts hypothetical responses to native NTs and mimics, where concentration ranges are much lower for the native protein ligands because high mimic doses are required to give comparable responses. Figure 9b,c are different insofar as NT concentrations are *fixed*, but concentrations for mimics are progressively increased. Figure 9b plots binding site fractions occupied by NT (gray) or mimic (green). Ligand (NT or mimic) occupying Trk binding sites are mostly NT initially, but progressively more mimic binds as its concentration is increased. Figure 9c shows responses typical of **5c(ii)** in ELFI (purple) corresponding to Figure 9b assuming that NTs elicit significantly greater response than the undetectable response of our mimics. Thus, most Trk sites are occupied by NTs at low mimic doses, and this triggers maximal responses. Mimics are prevalent binders at elevated concentrations because they displace NTs, but they elicit less response per binding event. *Agonistic mimics can appear to be antagonists because at high concentrations they bind Trks competitively, but in fact, they are simply less potent agonists*.

In the primary adult cortical neuron assays, **5c(ii)** performed as expected. This compound facilitated survival in HeLa+A cells (Figure 2) and did the same in primary adult mouse neurons known to express TrkA (Figure 7b).^84^ Using primary adult neurons in this type of assay is critical for compounds designed to have potential therapeutic effects, since there are age-dependent effects on the expression of TrkA in the brain.^91^ Most neurological disorders are diagnosed in adults, not embryos, highlighting the importance of using adult neuronal cells in confirmatory assays which are more relevant to diseases.^85,86^ In addition, even the effect of gene therapy on CNS injury has been shown to be age-dependent, and promoting axon growth is reduced with age.^92−94^

We assert our selected compounds are *agonists or modulators* based on: (i) positive cell survival; (ii) direct observation of significantly more mimic binding to Trk transfectants than corresponding *Trk*- cells; and (iii) primary neuron survival in the neurite outgrowth and survival assay. The most pronounced ambiguity in this series was **5c(ii)** which was an agonist in cell survival assays but gave *apparent* antagonism in ELFI assays. Further, some of the other compounds which proved to be agonists or modulators of cell survival did *not* stimulate phosphorylation mediated by Trk receptors. However, we believe that the explanation for this offered in Figure 9 reconciles these discrepancies.

Our data suggest that, with an increase in Trk receptor expression, there is an increase in cellular binding of our fluorescent compounds. One potential application of this discovery would be to determine relative levels of Trk expression *in vitro*, since we observe minimal off-target binding. Another envisioned potential application (though outside the scope of this publication) is its use *in vivo* in rodent cancer models, where Trk expression is correlated with cancer. Administering our compounds systemically in an animal in this case, followed by fixating and sectioning the region of interest, allows one to image and quantify the relative fluorescence presence in a particular region, which is potentially an indirect determination of the relative expression of a Trk receptor.

## Methods

### Synthesis and Characterization of Compound Series **5**

Peptides were synthesized according to standard Fmoc-^t^Bu solid-phase peptide synthesis (SPPS) protocols. BODIPY was synthesized and cyclized with peptides as previously reported on a 0.02 mmol scale.^81^ Purification was conducted via preparative-HPLC (Varian/Agilent SD-1 pump modules, Agilent 1260 DAD UV–vis detector) with a 30–95% MeCN/H_2_O + 0.1% TFA gradient (Agilent 5 Prep-C18 column, 5 μm particle size, ID 30 mm, length 100 mm). Compounds were characterized via ^1^H and TOCSY NMR (Bruker Avance III, 400 MHz, room temperature, solvent = 90% H_2_O and 10% D_2_O), high-resolution mass spectrometry (electrospray ionization in negative mode), analytical HPLC (Agilent 1260 Infinity II), UV–vis (Cary 100 Bio UV–visible Spectrophotometer), and fluorescence (Cary Eclipse Fluorescence Spectrophotometer). Detailed characterization information including NMR, MS, HPLC, and UV–vis/fluorescence spectra for all compounds is included in the SI.

### General Cell Culture

Dulbecco’s Modified Eagle’s Medium-high glucose (DMEM-high glucose) and Dulbecco’s Modified Eagle’s Medium/Nutrient Mixture F-12 Ham (DMEM/F12) were purchased from MilliporeSigma. Fetal Bovine Serum (FBS), Horse Serum (HS), Newborn Calf Serum (NBCS), and Penicillin–Streptomycin (PS) were purchased from Corning. Cell lines were cultured in sterile T-75 culture flasks in complete media (HeLa, HeLa-TrkA, HEK293, HEK293-TrkB: DMEM-high glucose + 10% FBS + 1% PS; NIH3T3 and NIH3T3-TrkC: DMEM/F12 + 10% NBCS + 1% PS; PC12: DMEM-high glucose + 10% HS + 5% FBS + 1% PS) at 37 °C in a humidified atmosphere containing 5% CO_2_ and split upon reaching 70% confluency.

### Cytotoxicity Assays

Cells were seeded at a density of 1 × 10^4^ cells/well in complete media in 96-well plates and incubated for 24 h to allow cells to adhere. Media was refreshed, and cells were treated with the compounds in a serial dilution for 24 h. Cells were washed with DPBS buffer twice and detached from the plate with 50 μL of trypsin for 3 min at 37 °C. Trypsinization was quenched with 100 μL of complete media. Cells were suspended, and live cells were counted using flow cytometry (CytoFLEX LX). Data is quantified and reported in Figure S1.

### Cell Survival Assays

Cells were seeded at a density of 2 × 10^3^ cells/well in complete media in 96-well plates and incubated for 24 h to allow cells to adhere. Cells were washed with DPBS, and then, media were swapped for serum-free media of the same type as the complete media. Cells were then treated with 50 μM compound either alone or in conjunction with suboptimal neurotrophin corresponding to the Trk receptor expressed for 48 h. Cells were washed with DPBS buffer twice and detached from the plate with 50 μL of trypsin for 3 min at 37 °C. Trypsinization was quenched with 100 μL of complete media. Cells were suspended and live cells counted using flow cytometry (CytoFLEX LX). Cell survival was normalized from cell count (CC) readings relative to optimal neurotrophin (NT = 100%) and DMSO (0%) utilizing GraphPad Prism 10.2, followed by statistical analysis via one-way ANOVA and Dunnett’s *t* test compared to the DMSO control. Dose response versions of this assay were conducted following the same procedure, but the data was analyzed in GraphPad Prism 10.2 using the nonlinear fit: {agonist} vs response – variable slope (four parameters) function.

### Trk-Expressing Cell Surface Binding Assays

Trk-positive transfected cell lines (HeLa-TrkA, HEK293-TrkB, and NIH3T3-TrkC) and Trk-negative cell lines (HeLa, HEK293, and NIH3T3) were seeded at a density of 2 × 10^3^ cells/well in 96-well plates and incubated for 24 h to allow them to adhere. Cells were treated with a serial dilution of the compound in serum-free media (SFM) with and without neurotrophin (NT) in Trk-positive cells (0.2 nM NGF in HeLa-TrkA; 0.6 nM BDNF in HEK293-TrkB; and 0.2 nM NT-3 in NIH3T3-TrkC) and without NT in the Trk-negative cell lines for 2.5 h. Cells were washed with DPBS to remove unbound fluorescent compound and dissolved in 1% (w/v) aqueous sodium dodecyl sulfate. Cell associated fluorescence was then determined by measuring emission of the resulting solution on a plate reader (BioTek Synergy H4 Hybrid Reader), λ_ex_ (540/25 nm) and λ_em_ (620/40 nm). *K*_d_ was calculated by subtracting the fluorescence observed in Trk-negative cells from that observed in Trk-positive cells of the corresponding type and then using the GraphPad Prism 10.2 function nonlinear fit: one site – specific binding.

### Enzyme-Linked Fixed-Cell Immunoassays (ELFI)

Assays were conducted as previously reported.^36^ Trk-positive cells (PC12, HEK293-TrkB, and NIH3T3-TrkC) were seeded at a density of 2 × 10^4^ cells per well in poly-d-lysine (PDL)-coated 96-well white flat-bottomed plates and allowed to adhere for 24 h. Cells were washed once with SFM and then incubated for 1–2 h in SFM. A serial dilution of compound and/or NT was added, and the cells were incubated for 15 min (agonism experiments) or 1 h (antagonism). Cells were then washed with DPBS and then fixed for 20 min with buffered 4% formaldehyde solution. Cells were washed and permeabilized 6 times with washing buffer (WB; 0.01 M PBS, 0.05%/v Tween-20, pH 7.4) and then blocked for 1 h with blocking buffer (BB; 0.01 M PBS, 0.05%/v Tween-20, 10% BSA, pH 7.4). Cells were incubated with primary antibody (anti-pAkt {Phospho-Akt (Ser473) (D9E) XP Rabbit mAb #4060, Cell Signaling Technology} 1:200 dilution or anti-pMAPK {Phospho-p44/42 MAPK (Erk1/2) (Thr202/Tyr204) (D13.14.4E) XP Rabbit mAb #4370, Cell Signaling Technology} 1:100 dilution) diluted in WB + 0.1% BSA for 4 h at room temperature and then washed 6× with WB, followed by incubation for 1 h with 1:1000 dilution of 2° antibody-HRP conjugate (Antirabbit IgG, HRP-linked Antibody #7074, Cell Signaling Technology). Cells were washed 6× with WB, and then, levels of phosphorylation were quantified using SuperSignal ELISA Pico Chemiluminescent Substrate (Thermo Scientific). Data are normalized to DMSO (0%), and maximum signal imparted by neurotrophin (10 ng/mL NT, 100%). The antibodies are stripped by treating with stripping buffer (SB; 6 M guanidine-HCl, 0.2%/v Triton X-100, 20 mM tris-HCl, pH 7.5) for 5 min, followed by 6 washes with WB. The process is then repeated on the same cells using the other primary antibody. Statistical analyses are carried out using two-way ANOVA followed by Dunnett’s *t* test. IC_50_ is calculated for **5c(ii)** using the {inhibitor} vs response – variable slope (four parameters) function in GraphPad Prism 10.2.

### Primary Adult Cortical Neuron Assay

Cortical neuron assay was conducted as previously described.^85^ Briefly, wild-type 6-week-old C57Bl/6 male mice were euthanized using CO_2_, and the brains were removed. The cortex was isolated and transferred to a MACS C-tube and then dissociated using the Miltenyi gentleMACS octo-dissociator on a preset protocol designed for adult rodent brains. This was followed by the removal of debris and endothelial blood cells using a Mitlenyi MACS Adult Brain Dissociation Kit (Miltenyi Biotec, 130-107-677). Afterward, following manufacturer’s instructions, the Adult Neuron Isolation Kit (Miltenyi Biotec, 130-126-603) was used, and the negative fraction containing an enriched neuronal population was collected and seeded onto PDL coated plastic bottom plates (Greiner-Bio, 781091) at 10,000 cells/well for 2 days. **5c(ii)** and Vehicle were added at the time of plating and left in the media for the entire 2 days. No media changes occurred during the experiment. Neuronal media consisted of MACS Neuro Media (Miltenyi Biotec, 130-093-570), 2 mM l-alanine-l-glutamine dipeptide (Sigma-Aldrich, G8541-100 ML), and 1× B-27 Plus Supplement (ThermoFisher Scientific, A3582801). Immunocytochemistry and imaging were conducted as previously described.^85^ First, the cells were fixed with 4% PFA, followed by an overnight incubation with the Class III β-tubulin (TUBB3, BioLegend, 802001) antibody at room temperature. The next day, the cells were stained with an Alexa Fluor (ThermoFisher Scientific, A32723) secondary antibody for 1 h and DAPI for 5 min at room temperature. Images were acquired using the 20× magnification lens of the Zeiss Axio Observer 7 microscope. Images were quantified using the Neurite Outgrowth Analysis Module in MetaXpress 6 software (Molecular Devices). For quantification, the number of valid neurons is determined by quantifying the number of TUBB3^+^/DAPI^+^ cells in a well with ≥10 μm of total neurite outgrowth. Total neurite outgrowth is determined by dividing the length of all neurites in a well by the number of valid neurons in that respective well. Only wild-type C57Bl/6 mice were used herein. All procedures were conducted according to the protocol approved by the Institutional Review Board/Animal Ethics Committee of Texas A & M University (IACUC 2023-0173).

## Supporting Information Available

Full characterization data (^1^H and TOCSY NMR, HRMS, HPLC, UV–vis, and fluorescence spectra) for all compounds synthesized; compound cytotoxicity assays on HeLa-TrkA, HEK293-TrkB, NIH3T3-TrkC, and PC12 cells (PDF)
