# Supplementary material for: Selective, Intrinsically Fluorescent Trk Modulating Probes
Source: ACS Chem Neurosci. 2024 Oct 2;15(20):3679–91. doi: 10.1021/acschemneuro.4c00290 (PMC11487604; doi:10.1021/acschemneuro.4c00290)
Supplement: Supplementary file 1 — cn4c00290_si_001.pdf [file cn4c00290_si_001.pdf]

SUPPORTING INFORMATION

Selective, Intrinsically Fluorescent, Trk Modulator Probes

Thitima Pewklang,[1,2] Tye Thompson,[1] Arthur Sefiani,[3,4] Cédric G. Geoffroy,[3,4] Anyanee Kamkaew,[2] and Kevin Burgess*[1]

[1] *Department of Chemistry, Texas A & M University, Box 30012, College Station, TX 77842-3012, USA.*

[2] *School of Chemistry, Institute of Science, Suranaree University of Technology, Nakhon Ratchasima 30000, Thailand.*

[3] *Department of Neuroscience and Experimental Therapeutics, Texas A&M University Health Science Center, Bryan, TX, 77807, United States.*

[4] *NeuroCreis, Inc., College Station, TX, 77840, United States.*

E-mail: burgess@tamu.edu

Compound Synthesis and Characterization

All reagents have been purchased from commercially available sources and used without further purification. Compounds were synthesized, purified, and characterized according to our previously reported methodology on a 0.02 mmol scale.[1] ^{1}H and TOCSY-NMR spectra were recorded on Bruker Avance III at 400 MHz at room temperature in solvent of 90% H_2O + 10% D_2O. Chemical shifts of ^{1}H NMR spectra

were reported in ppm as follows: chemical shift, multiplicity (s = singlet, d = doublet, t = triplet, m = multiplet, dd = doublet of doublet), coupling constants, and number of protons. Mass spectra (MS) were measured by high-resolution electrospray ionization mass spectrometry (ESI-MS) in negative mode. Analytical HPLC was performed on an Agilent 1260 Infinity II system with 5-95% MeCN in water gradient with 0.1% TFA over 10 min using an Eclipse XDB-C18 reverse-phase column with 5 µm particle size, ID 4.6 mm, length 250 mm. Prep HPLC was performed on an Agilent PrepStar in a 30-95% MeCN/water gradient with 0.1% TFA over 25 mins using an Agilent 5 Prep-C18 column with 5 µm particle size, ID 30 mm, length 100 mm. All statistical analyses were carried out by GraphPad Prism version 10.2 or later.

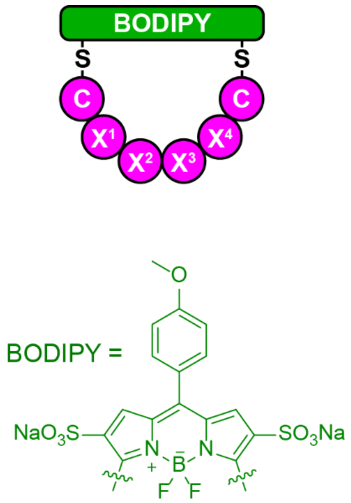

compound	X^1	X^2	X^3	X^4
**5a(i)**	D	I	K	G
**5a(ii)**	I	N	N	S
**5a(iii)**	D	G	K	Q
**5a(iii)m**	D	E	K	Q
**5b(i)**	D	M	S	G
**5b(ii)**	V	S	K	G
**5b(iii)**	D	S	K	K
**5c(i)**	D	I	R	G
**5c(ii)**	T	Q	N	S
**5c(ii)m**	T	G	N	S
**5c(iii)**	E	N	N	K
**5d(i)**	D	L	R	G
**5d(ii)**	A	G	G	S
**5d(iii)**	D	A	Q	G

5a(i) The purity was found to be 98% by HPLC analysis at 280 and 550 nm detection, retention time 8.662 min. The isolated yield was 1.2 mg (6%). ^{1}H NMR (400 MHz, 90% H_2O + 10% D_2O) δ 8.46 (d, *J* = 7.29 Hz,1H), 8.41 (d, *J* = 6.45 Hz, 1H), 8.18 (d, *J* = 5.87 Hz, 1H), 8.10 (t, *J* = 6.38 Hz, 1H), 7.98 (d ,*J* = 7.91 Hz, 1H), 7.72 (d, *J* = 6.63 Hz,1H), 7.65 (d, *J* = 9.23 Hz, 2H), 7.57 (s, 3H), 7.45 (s, 1H), 7.37 (s, 1H), 7.22 (d, *J* = 9.25 Hz, 2H), 7.14 (s, 2H), 4.14-4.15 (m, 1H), 4.10-4.12 (m, 1H), 3.91-3.97 (m, 2H), 3.94 (s, 3H), 3.74 (dd, *J* = 13.86, 6.27 Hz, 2H), 3.58-3.60 (m, 1H), 3.55-3.57 (m, 1H), 2.90-2.96 (m, 2H), 2.79-2.86 (m, 2H), 2.04-2.06 (m, 2H), 1.77-1.82 (m, 1H), 1.72-1.96 (m, 1H), 1.59-1.68 (m, 2H), 1.38-1.44 (m, 2H), 1.28-1.36 (m, 1H), 1.08-1.18 (m, 1H), 0.83 (t, *J* = 7.96 Hz, 3H), 0.71 (t, *J* = 7.96 Hz, 3H) High Resolution ESI$^-$: m/z calcd for $[C_{42}H_{53}BF_2N_{10}O_{16}S_4]^{2-}$ 565.1287 found 565.1305.

5b(i) The purity was found to be 91% by HPLC analysis at 280 and 550 nm detection, retention time 7.438 min. The isolated yield was 5.1 mg (24%). ^{1}H NMR (400 MHz, 90% H_2O + 10% D_2O) δ 8.55 (d, *J* = 7.10 Hz, 1H), 8.45 (d, *J* = 5.76 Hz, 1H), 8.33 (s, 1H), 8.31 (s, 1H), 8.14 (d, *J* = 12.66 Hz, 1H), 7.86 (d, *J* = 7.89 Hz, 2H), 7.65 (d, *J* = 9.37 Hz, 2H), 7.46 (s, 1H), 7.36 (s, 1H), 7.22 (d, *J* = 9.37 Hz, 2H), 7.08 (s, 2H), 4.29-4.33 (m, 1H), 4.07-4.17 (m, 1H), 3.94 (s, 3H), 3.92 (s, 1H), 3.91 (s, 1H), 3.90 (s, 2H), 3.88 (s, 1H), 3.86 (s, 1H), 3.82 (d, *J* = 5.64 Hz, 2H), 3.95 (d, *J* = 5.09 Hz, 1H), 3.71 (d, *J* = 5.13 Hz, 1H), 3.58 (d, *J* = 3.63 Hz, 1H), 3.54 (d, *J* = 5.38 Hz, 1H), 2.74-2.85 (m, 2H), 2.60-

2.71 (m, 2H), 2.12-2.21 (m, 2H), 2.04 (s, 3H), 1.99-2.06 (m, 3H) High Resolution ESI$^-$: m/z calcd for $[C_{38}H_{44}BF_2N_9O_{17}S_5]^{2-}$ 553.5754 found 553.5773.

5c(i) The purity was found to be 100% by HPLC analysis at 280 and 550 nm detection, retention time 7.035 min. The isolated yield was 8.6 mg (39%). ^{1}H NMR (400 MHz, 90% H_2O + 10% D_2O) δ 8.45 (d, *J* = 6.91 Hz, 1H), 8.42 (d, *J* = 6.15 Hz, 1H), 8.10 (s, 1H), 8.07 (s,1H), 8.05 (s,1H), 7.75 (d, *J* = 7.07 Hz, 1H), 7.62 (d, *J* = 9.41 Hz, 2H), 7.55 (s,1H), 7.44 (s,1H), 7.32 (s,1H), 7.19 (d, *J* = 9.45 Hz, 2H), 7.05-7.12 (m, 4H), 4.15-4.19 (m,1H), 4.09-4.13 (m,1H), 3.98 (d, *J* = 5.36 Hz, 2H), 3.93 (s, 3H), 3.88 (d, *J* = 5.36 Hz, 2H), 3.75 (d, *J* = 6.59 Hz, 1H), 3.72 (d, *J* = 6.28 Hz, 1H), 3.62 (d, *J* = 6.43 Hz, 1H), 3.52-3.59 (m,1H), 3.06 (d, *J* = 6.60 Hz, 2H), 2.90-2.96 (m, 1H), 2.82-2.88 (m, 2H), 2.04 (s, 3H), 1.78-1.83 (m, 1H), 1.71-1.76 (m, 2H), 1.53-1.57 (m, 2H), 1.27-1.35 (m, 1H), 1.07-1.18 (m, 1H), 0.84 (d, *J* = 7.36 Hz, 3H), 0.73 (t, *J* = 16.47 Hz, 3H) High Resolution ESI$^-$: m/z calcd for $[C_{42}H_{53}BF_2N_{12}O_{16}S_4]^{2-}$ 579.1318 found 579.1332.

5d(i) The purity was found to be 91% by HPLC analysis at 280 and 550 nm detection, retention time 6.861 min. The isolated yield was 3.2 mg (14%). ^{1}H NMR (400 MHz, 90% H_2O + 10% D_2O) δ 8.56 (d, *J* = 6.31 Hz, 1H), 8.41 (d, *J* = 6.21 Hz, 1H), 8.08 (d, *J* = 6.56 Hz, 1H), 8.04 (s, 1H), 8.02 (s, 1H), 8.01 (s, 1H), 7.65 (d, *J* = 9.35 Hz, 2H), 7.48 (s, 1H), 7.45 (s, 1H), 7.35 (s, 1H), 7.21 (d, *J* = 9.40 Hz, 2H), 7.09 (s, 2H), 4.31-4.34 (m, 1H), 4.19-4.24 (m,1H), 4.09-4.10 (m, 2H), 4.05 (d, *J* = 7.33 Hz, 1H), 3.87-3.91 (m, 2H), 3.84 (d, *J* = 5.12, 1H), 3.58-3.75 (m, 2H), 3.45-3.52 (m, 1H), 3.03-3.08 (m, 2H), 2.65-2.83 (m, 2H), 2.06 (s, 3H), 1.72-1.79 (m, 2H), 1.60-1.68 (m, 2H), 1.53-1.59 (m, 2H), 1.22 (s, 1H), 0.82-0.89 (m, 6H) High Resolution ESI$^-$: m/z calcd for $[C_{42}H_{53}BF_2N_{12}O_{16}S_4]^{2-}$ 579.1318 found 579.1332.

5a(ii) The purity was found to be 98% by HPLC analysis at 280 and 550 nm detection, retention time 7.996 min. The isolated yield was 2.8 mg (13%). ^{1}H NMR (400 MHz, 90% H_2O + 10% D_2O) δ 8.49 (d, *J* = 7.30 Hz, 1H), 8.42 (s, 1H), 8.41 (s, 1H), 8.28 (d, *J* = 7.59 Hz, 1H), 8.13, (d, *J* = 7.78 Hz, 1H), 7.85 (d, *J* = 6.82 Hz, 1H), 7.73 (d, *J* = 6.40 Hz, 1H), 7.66 (d, *J* = 9.37 Hz, 2H), 7.52 (s, 1H), 7.49 (s, 1H), 7.33 (s,1H), 7.22 (d, *J* = 9.36 Hz, 2H), 7.10 (s, 1H), 7.05 (s, 1H), 6.82 (s, 1H), 4.14-4.16 (m, 1H), 4.10-4.12 (m, 1H), 3.97 (s, 1H), 3.96 (s, 1H), 3.94 (s, 1H), 3.75-3.79 (m, 1H), 3.69-3.73 (m, 1H), 3.55-3.60 (m, 1H), 3.48-3.54 (m, 1H), 2.83-2.88 (m, 2H), 2.67-2.73 (m, 2H), 2.20-2.28 (m, 2H), 2.05 (s, 3H), 1.83-1.88 (m, 1H), 1.45-1.53 (m, 1H), 1.16-1.28 (m, 1H), 0.90 (d, *J* = 7.64 Hz, 3H), 0.85 (d, *J* = 8.00 Hz, 3H). High Resolution ESI$^-$: m/z calcd for $[C_{41}H_{50}BF_2N_{11}O_{17}S_4]^{2-}$ 572.6161 found 572.6172.

5b(ii) The purity was found to be 96% by HPLC analysis at 280 and 550 nm detection, retention time 7.619 min. The isolated yield was 5.4 mg (26%). ^{1}H NMR (400 MHz, 90% H_2O + 10% D_2O) δ 8.43 (d, *J* = 6.54 Hz, 1H), 8.38 (d, *J* = 6.83 Hz, 1H), 8.16, (d, *J* = 7.07 Hz, 1H), 8.12, (d, *J* = 7.97 Hz, 1H), 7.88, (d, *J* = 5.96 Hz, 1H), 7.77, (d, *J* = 7.11 Hz, 1H), 7.65 (d, *J* = 9.33 Hz, 2H), 7.52 (s, 1H), 7.47 (s, 1H), 7.33, (s, 1H), 7.21 (d, *J* = 9.44 Hz, 2H), 7.10 (s, 1H), 4.18-4.23 (m, 1H), 4.02 (s, 1H), 3.97 (s, 1H), 3.94 (s, 3H), 3.88 (s, 2H), 3.86 (s, 2H), 3.67-3.69 (m, 1H), 3.63-3.66 (m, 2H), 3.58-3.60 (m, 2H), 3.55-3.56 (m, 1H), 2.82-2.85 (m, 2H), 2.15 (m, 1H), 2.06 (s, 3H), 1.61-1.70 (m, 2H),

1.44-1.54 (m, 2H), 1.22-1.26 (m, 2H), 0.96 (t, *J* = 8.63 Hz, 6H). High Resolution ESI$^-$: m/z calcd for $[C_{40}H_{51}BF_2N_{10}O_{15}S_4]^{2-}$ 544.1234 found 544.1248.

5c(ii) The purity was found to be 96% by HPLC analysis at 280 and 550 nm detection, retention time 7.236 min. The isolated yield was 1.3 mg (6%). ^{1}H NMR (400 MHz, 90% H_2O + 10% D_2O) δ 8.51 (t, *J* = 5.82 Hz, 1H), 8.48 (s, 1H), 8.46 (s, 1H), 8.36 (d, *J* = 7.21 Hz, 1H), 8.29 (d, *J* = 7.84 Hz, 1H), 8.10 (d, *J* = 6.76 Hz, 1H), 7.65 (d, *J* = 10.19 Hz, 2H), 7.50 (s, 1H), 7.46 (s, 1H), 7.37 (s,1H), 7.21 (d, *J* = 8.83 Hz, 2H), 4.27-4.41 (m, 1H), 4.04-4.05 (m, 1H), 4.00 (s, 1H), 3.98 (s,1H), 3.97 (s, 1H), 3.93 (s, 2H), 3.92 (s, 1H), 3.87-3.89 (m, 2H), 3.63-3.68 (m, 2H), 3.54-3.60 (m, 2H), 2.87-2.92 (m, 2H), 2.72-2.78 (m, 2H), 2.00-2.05 (m, 1H), 2.03 (s, 3H), 1.21 (d, *J* = 6.44 Hz, 3H). High Resolution ESI$^-$: m/z calcd for $[C_{40}H_{48}BF_2N_{11}O_{18}S_4]^{2-}$ 573.6056 found 573.6074.

5c(ii)m The purity was found to be 97% by HPLC analysis at 280 and 550 nm detection, retention time 8.788 min. The isolated yield was 4.5 mg (22%). ^{1}H NMR (400 MHz, 90% H_2O + 10% D_2O) δ 8.50 (s, 1H), 8.47 (s, 1H), 8.07 (d, *J* = 7.70 Hz, 1H), 7.91 (d, *J* = 6.85 Hz, 1H), 7.65 (d, *J* = 9.71 Hz, 2H), 7.47 (s, 1H), 7.40 (s, 1H), 7.35 (s, 1H), 7.22 (d, *J* = 9.74 Hz, 2H), 7.10 (s, 1H), 4.28-4.33 (m, 1H), 4.13-4.18 (m, 1H) 3.94 (s, 3H), 3.88 (d, *J* = 5.98 Hz, 2H), 3.85 (d, *J* = 5.77 Hz, 1H), 3.81 (d, *J* = 4.41 Hz, 1H), 3.78 (d, *J* = 4.77 Hz, 2H), 3.63 (d, *J* = 6.45 Hz, 2H), 3.45 (dd, *J* = 15.17, 11.09 Hz, 1H), 2.45-2.50 (m, 2H), 2.31 (t, *J* = 7.88 Hz, 2H), 2.08-2.15 (m, 1H), 2.16 (s, 3H), 1.94-2.00 (m, 1H), 1.23 (d, *J* = 6.37 Hz, 3H). High Resolution ESI$^-$: m/z calcd for $[C_{37}H_{43}BF_2N_{10}O_{17}S_4]^{2-}$ 538.0870 found 538.0884.

5d(ii) The purity was found to be 92% by HPLC analysis at 280 and 550 nm detection, retention time 6.624 min. The isolated yield was 2.2 mg (12%). ^{1}H NMR (400 MHz, 90% H_2O + 10% D_2O) δ 8.65 (d, *J* = 3.91 Hz, 1H), 8.36 (d, *J* = 6.09 Hz, 1H), 8.32 (d, *J* = 7.58 Hz, 1H), 8.26 (s, 1H), 8.23 (d, *J* = 6.73 Hz, 1H), 7.67 (d, *J* = 10.03 Hz, 2H), 7.44 (s, 1H), 7.41 (s, 1H), 7.22 (d, *J* = 10.03 Hz, 2H), 7.07 (s, 1H), 4.24-4.28 (m, 1H), 4.08-4.14 (m, 1H), 4.05 (s, 1H), 3.98 (d, *J* = 5.06, 2H), 3.92 (d, *J* = 5.40 Hz, 2H), 3.81-3.82 (m, 1H), 3.74-3.78 (m, 2H), 3.66-3.70 (m, 2H), 3.49-3.55 (m, 2H), 1.98 (s, 3H), 1.41 (d, *J* = 7.76 Hz, 3H). High Resolution ESI$^-$: m/z calcd for $[C_{34}H_{38}BF_2N_9O_{15}S_4]^{2-}$ 494.5710 found 494.5721.

5a(iii) The purity was found to be 100% by HPLC analysis at 280 and 550 nm detection, retention time 6.541 min. The isolated yield was 7.0 mg (32%). ^{1}H NMR (400 MHz, 90% H_2O + 10% D_2O) δ 9.08 (d, *J* = 6.30 Hz, 1H), 8.27 (d, *J* = 6.15 Hz, 1H), 8.13 (t, *J* = 5.95 Hz, 1H), 7.86 (d, *J* = 7.38 Hz, 1H), 7.71 (d, *J* = 5.49 Hz, 1H), 7.65 (d, *J* = 9.44 Hz, 2H), 7.46 (s, 1H), 7.39 (s,1H), 7.34 (s, 3H), 7.21 (d, *J* = 9.45 Hz, 2H), 7.10 (s, 2H), 4.22-4.30 (m, 1H), 4.13-4.19 (m, 1H), 3.94 (s, 3H), 3.77-3.80 (m, 1H), 3.76-3.79 (m, 1H), 3.68-3.72 (m, 1H), 3.67-3.70 (m, 1H), 3.30 (d, *J* = 11.86 Hz, 1H), 3.27 (d, *J* = 11.94 Hz, 1H), 2.91-2.96 (m, 2H), 2.81-2.88 (m, 1H), 2.15-2.23 (m, 2H), 2.07-2.12 (m, 2H), 2.05 (s, 3H), 2.02-2.07 (m, 2H), 1.78-1.89 (m, 2H), 1.56-1.64 (m, 2H), 1.39-1.47 (m, 2H) High Resolution ESI$^-$: m/z calcd for $[C_{41}H_{50}BF_2N_{11}O_{17}S_4]^{2-}$ 572.6160 found 572.6175.

5a(iii)m The purity was found to be 97% by HPLC analysis at 280 and 550 nm detection, retention time 7.036 min. The isolated yield was 7.3 mg (31%). ^{1}H NMR (400 MHz, 90% H_2O + 10% D_2O) δ 8.58 (d, *J* = 5.00 Hz, 1H), 8.31 (d, *J* = 7.20 Hz, 1H), 8.27 (d, *J* = 7.31 Hz, 1H), 8.10 (d, *J* = 7.36 Hz, 1H), 8.06 (d, *J* = 7.29 Hz, 1H), 7.92 (d, *J* = 7.01 Hz, 1H), 7.65 (d, *J* = 9.79 Hz, 2H), 7.52 (s, 3H), 7.46 (S, 1H), 7.39 (S, 1H), 7.21 (d, *J* = 10.01 Hz, 2H), 7.07 (S, 2H), 4.26-4.33 (m, 1H), 4.18-4.24 (m, 1H), 3.94 (S, 3H), 3.90-3.92 (m, 1H), 3.85-3.86 (m, 1H), 3.76-3.83 (m, 2H), 3.65-3.72 (m, 2H), 3.62 (d, *J* = 4.72 Hz, 1H), 3.59 (d, *J* = 4.69 Hz, 1H), 2.95-3.02 (m, 2H), 2.83-2.93 (m, 2H), 2.43 (t, *J* = 16.13 Hz, 2H), 2.25 (t, *J* = 19.46 Hz, 2H), 2.18 (t, *J* = 19.46, 2H), 2.01 (s, 3H), 1.81-1.88 (m, 2H), 1.60-1.66 (m, 2H), 1.35-1.42 (m, 2H) High Resolution ESI$^-$: m/z calcd for $[C_{44}H_{54}BF_2N_{11}O_{19}S_4]^{2-}$ 608.6265 found 608.6286.

5b(iii) The purity was found to be 99% by HPLC analysis at 280 and 550 nm detection, retention time 8.897 min. The isolated yield was 1.5 mg (7%). ^{1}H NMR (400 MHz, 90% H_2O + 10% D_2O) δ 8.88 (d, *J* = 6.79 Hz, 1H), 8.39 (d, *J* = 6.02 Hz, 1H), 7.91 (d, *J* = 5.40 Hz, 1H), 7.72 (d, *J* = 7.18 Hz, 1H), 7.64 (d, *J* = 9.27 Hz, 2H), 7.58 (t, *J* = 17.35 Hz, 1H), 7.45 (s, 1H), 7.40 (s, 1H), 7.31 (s, 1H), 7.21 (d, *J* = 9.32 Hz, 2H), 7.10 (s, 2H), 4.18 (d, *J* = 4.05 Hz, 1H), 4.15 (d, *J* = 3.52 Hz, 1H), 4.06 (s, 1H), 4.04 (s, 1H), 3.94 (s, 3H), 3.75 (t, *J* = 10.59 Hz, 2H), 3.37-3.40 (m, 1H), 3.40-3.37 (m, 1H), 3.05 (d, *J* = 7.31 Hz, 1H), 3.01 (s, 1H), 2.97 (s, 2H), 2.85 (s, 2H), 2.80 (d, *J* = 6.74 Hz, 1H), 2.75 (d, *J* = 6.60 Hz, 1H), 2.03 (s, 3H), 1.98-2.04 (m, 2H), 1.81-1.87 (m, 2H), 1.70-1.81 (m, 2H), 1.64-1.66 (m, 2H), 1.59-1.62 (m, 2H), 1.48-1.55 (m, 2H), 1.36-1.46 (m, 2H), 1.26-1.28 (m, 2H), 1.22-1.24 (m, 2H) High Resolution ESI$^-$: m/z calcd for $[C_{43}H_{56}BF_2N_{11}O_{17}S_4]^{2-}$ 587.6394 found 587.6413.

5c(iii) The purity was found to be 98% by HPLC analysis at 280 and 550 nm detection, retention time 6.783 min. The isolated yield was 1.5 mg (7%). ^{1}H NMR (400 MHz, 90% H_2O + 10% D_2O) δ 8.77 (d, *J* = 6.29 Hz, 1H), 8.37 (d, *J* = 6.86 Hz, 1H), 8.30 (d, *J* = 7.67 Hz, 1H), 8.11 (d, *J* = 7.52 Hz, 1H), 7.97-8.01 (m, 1H), 7.93 (d, *J* = 6.00 Hz, 1H), 7.65 (d, *J* = 9.48 Hz, 2H), 7.51 (s, 1H), 7.47 (s, 1H), 7.38 (s, 1H), 3.36 (s, 3H), 7.22 (d, *J* = 9.55 Hz, 2H), 7.10 (s, 1H), 6.77 (s, 2H), 4.20-4.25 (m, 1H), 4.13-4.18 (m, 1H), 3.94 (s, 3H), 3.96 (s, 1H), 3.91 (s, 1H), 3.71-3.76 (m, 1H), 3.61-3.66 (m, 1H), 3.42 (d, *J* = 11.84 Hz, 1H), 3.38 (d, *J* = 11.53 Hz, 1H), 2.86-2.70 (m, 2H), 2.74-2.85 (m, 2H), 2.51-2.52 (m, 2H), 2.45-2.49 (m, 2H), 2.08-2.18 (m, 2H), 2.06 (s, 3H), 1.68-1.78 (m, 2H), 1.48-1.56 (m, 2H), 1.41-1.46 (m, 2H), 1.22-1.28 (m, 2H) High Resolution ESI$^-$: m/z calcd for $[C_{43}H_{53}BF_2N_{12}O_{18}S_4]^{2-}$ 601.1267 found 601.1285.

5d(iii) The purity was found to be 99% by HPLC analysis at 280 and 550 nm detection, retention time 7.078 min. The isolated yield was 7.2 mg (35%). ^{1}H NMR (400 MHz, 90% H_2O + 10% D_2O) δ 8.55 (t, *J* = 7.58 Hz, 1H), 8.30-8.32 (m, 1H), 8.26-8.28 (m, 1H), 8.09-8.11 (m, 1H), 8.00-8.01 (m, 1H), 7.89-7.99 (m, 1H), 7.65 (d, *J* = 9.33 Hz, 2H), 7.46 (s, 1H), 7.21 (d, *J* = 9.33 Hz, 2H), 7.10 (s, 1H), 7.05 (s, 1H), 6.92 (s, 1H), 4.21-4.25 (m, 1H), 4.04-4.06 (m, 1H), 3.97-4.00 (m, 1H), 3.94 (s, 3H), 3.88-3.91 (m, 2H), 3.84-3.85 (m, 1H), 3.78-3.80 (m, 1H), 3.75-3.76 (m, 1H), 3.57-3.61 (m, 1H), 3.52-3.55 (m, 1H), 2.86-2.34 (m, 2H), 2.46 (t, *J* = 7.83 Hz, 1H), 2.33 (t, *J* = 7.85 Hz, 1H), 2.07-2.14 (m, 2H),

2.02 (s, 1H), 1.42 (dd, J = 7.13, 3.80 Hz, 3H). High Resolution ESI$^-$: m/z calcd for $[C_{38}H_{43}BF_2N_{10}O_{17}S_4]^{2-}$ 544.0870 found 544.0884.

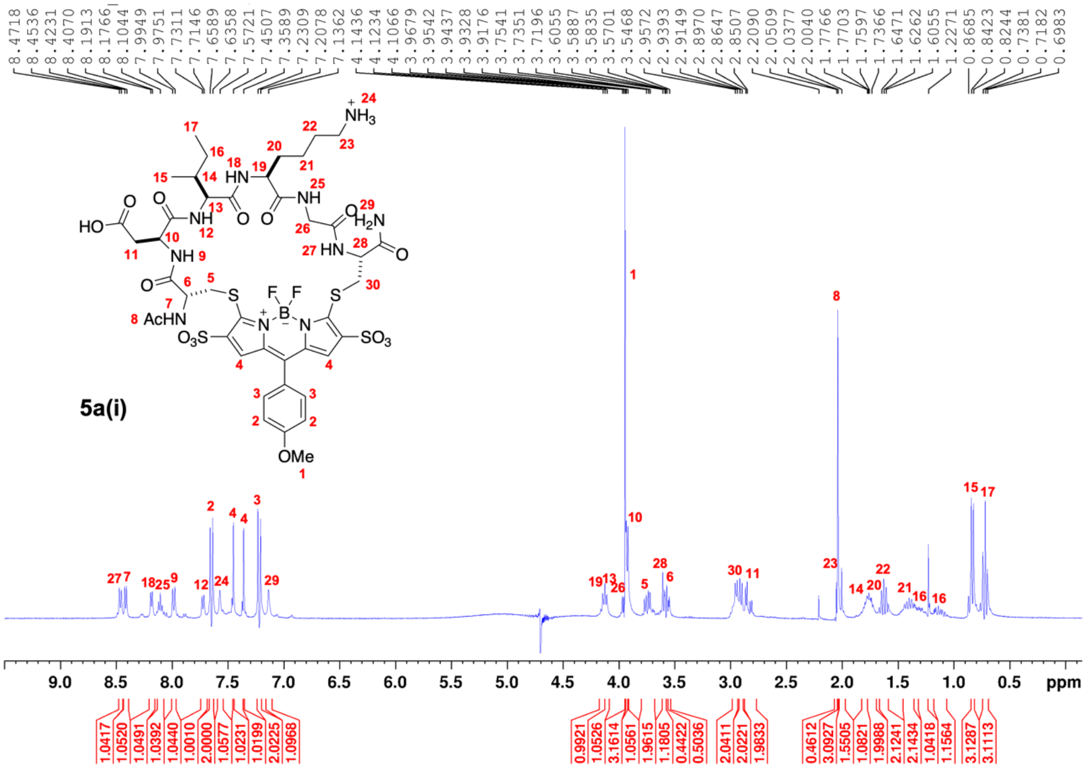

^{1}H NMR of 5a(i)

5a(i)

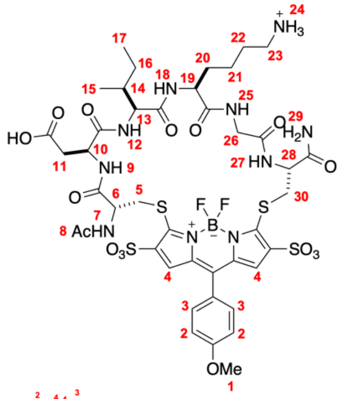

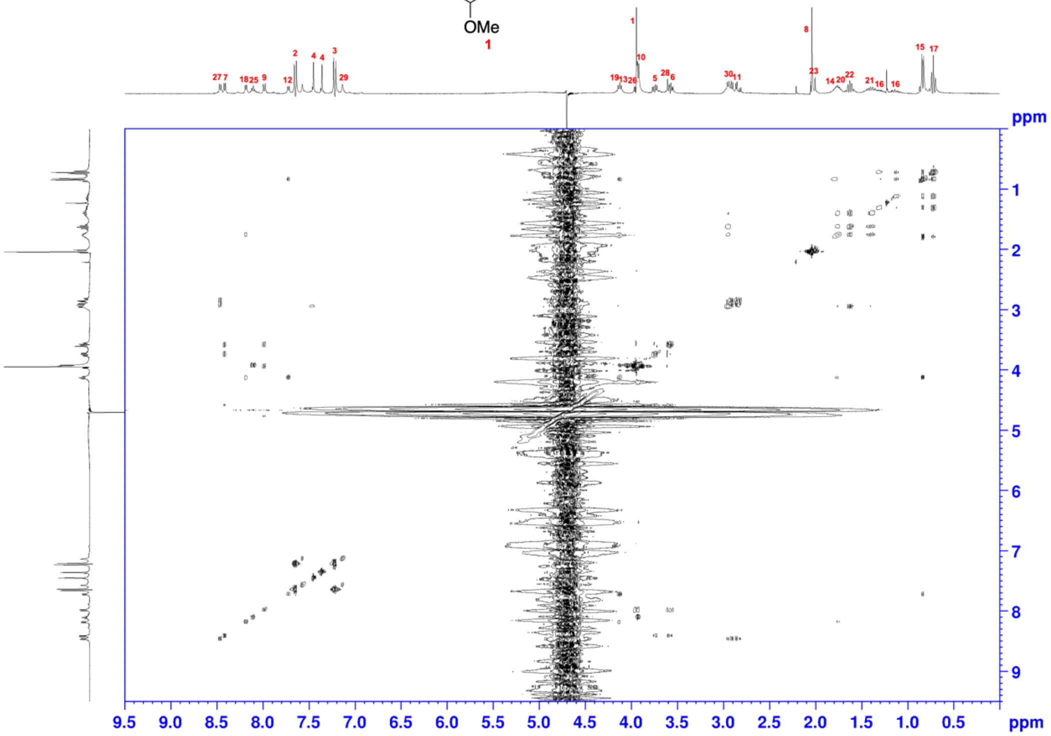

TOCSY of 5a(i)

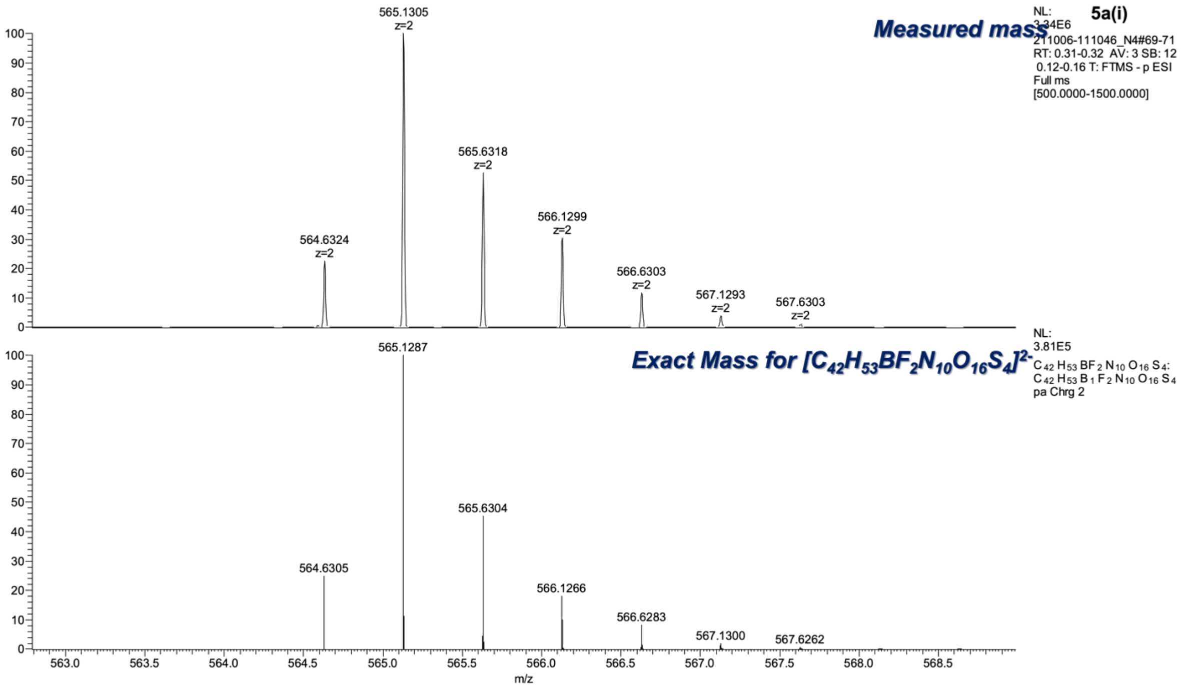

HRMS of 5a(i)

Analytical HPLC of **5a(i)** at 280 nm detection

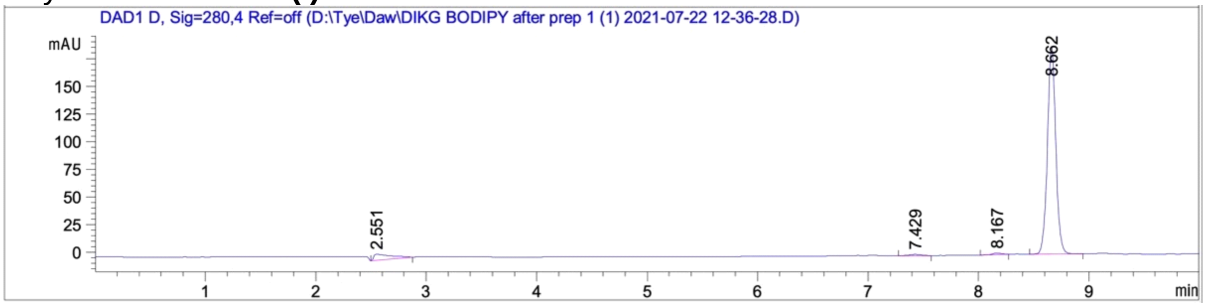

Analytical HPLC of **5a(i)** at 550 nm detection

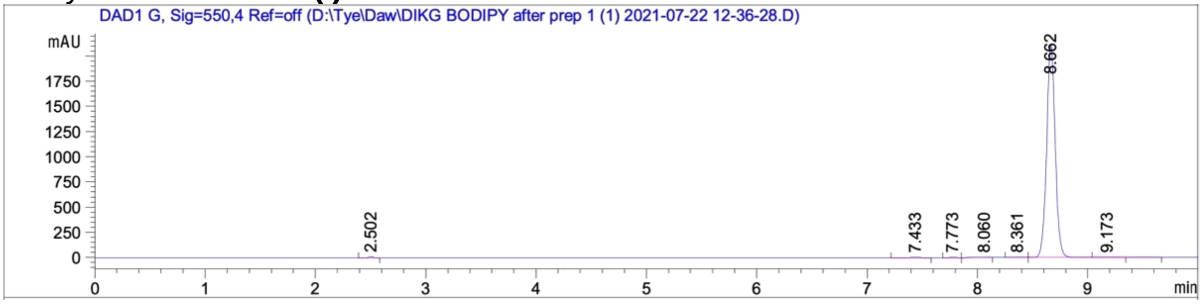

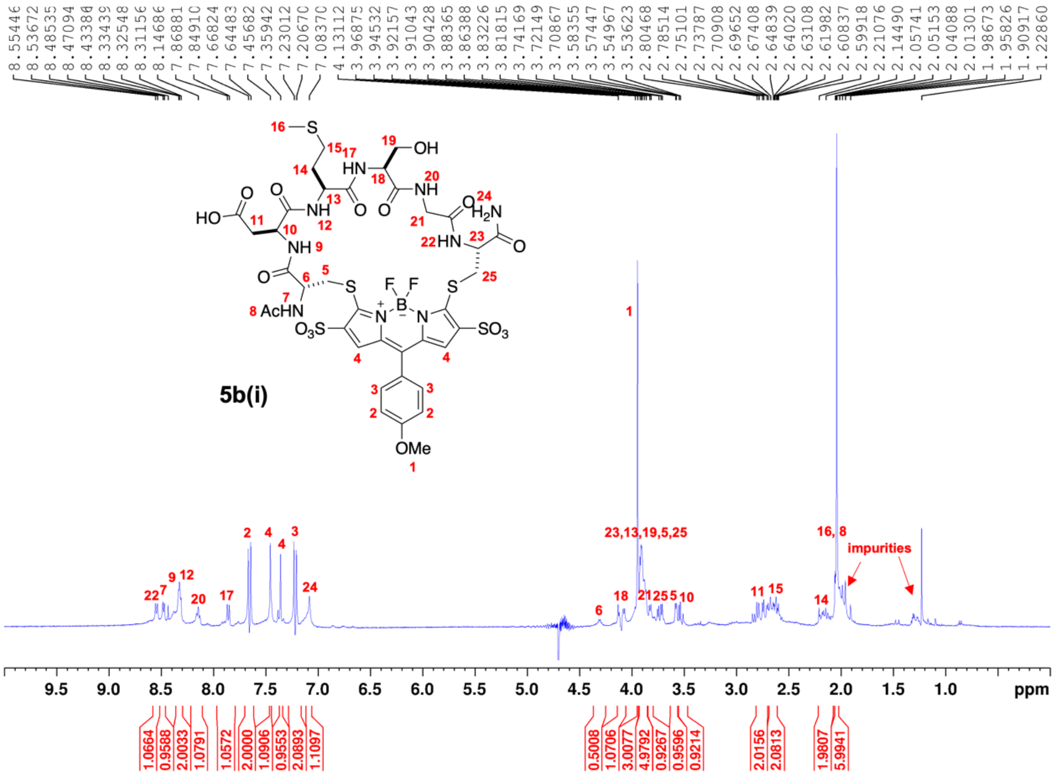

^1H NMR of 5b(i)

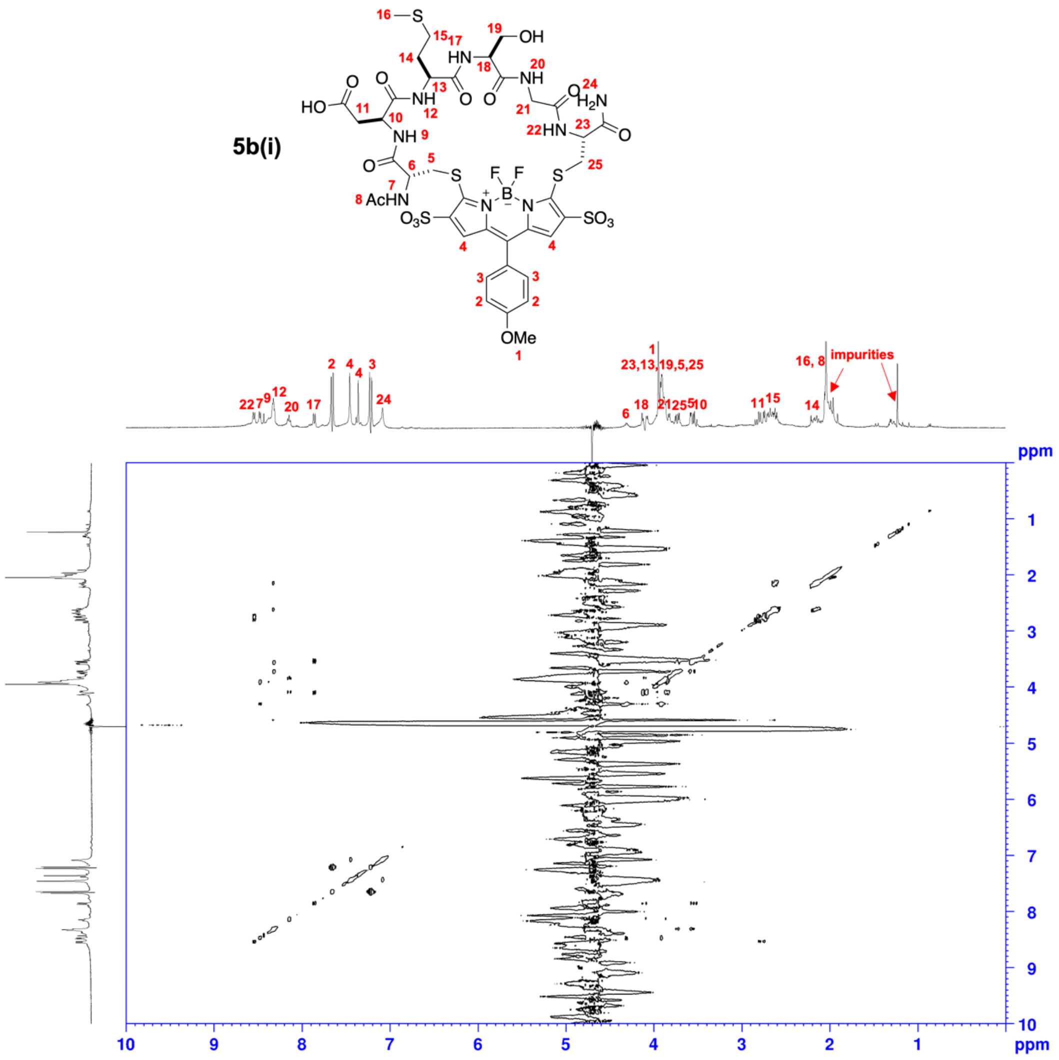

TOCSY of 5b(i)

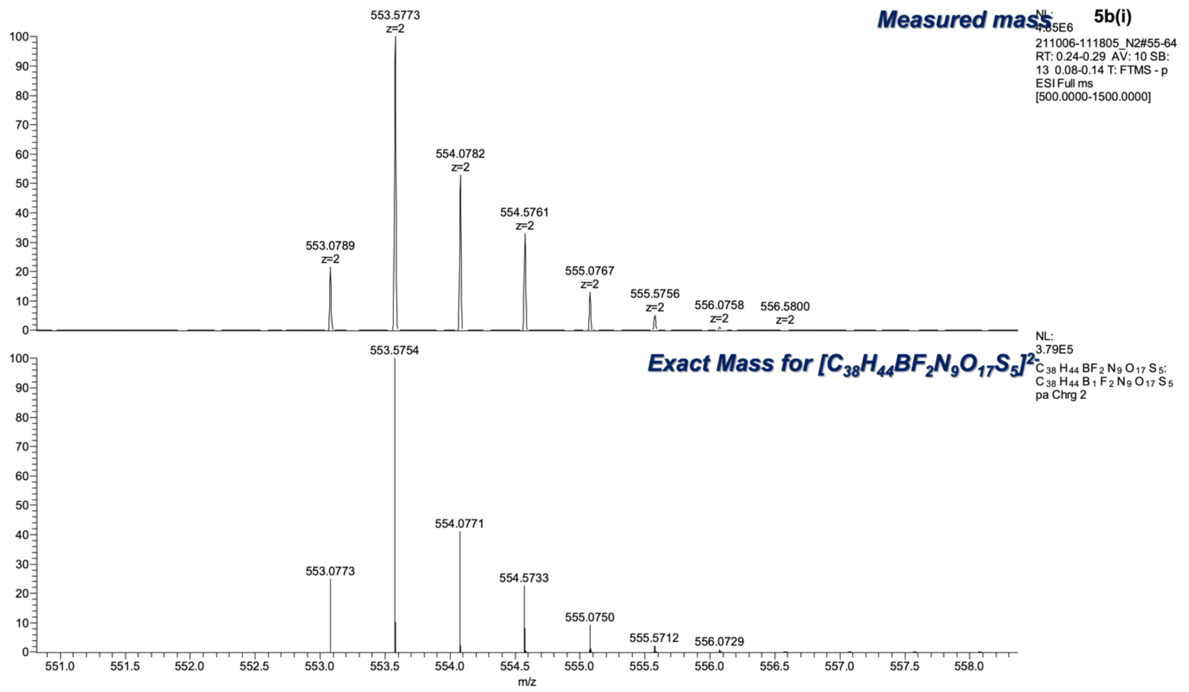

HRMS of 5b(i)

Analytical HPLC of **5b(i)** at 280 nm detection

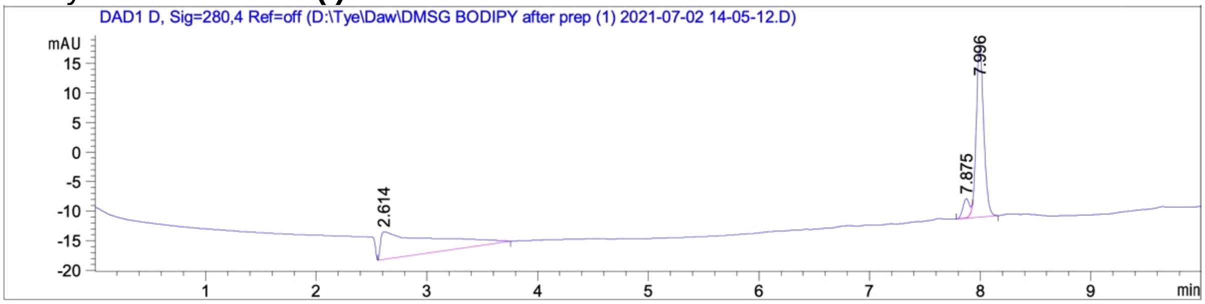

Analytical HPLC of **5b(i)** at 550 nm detection

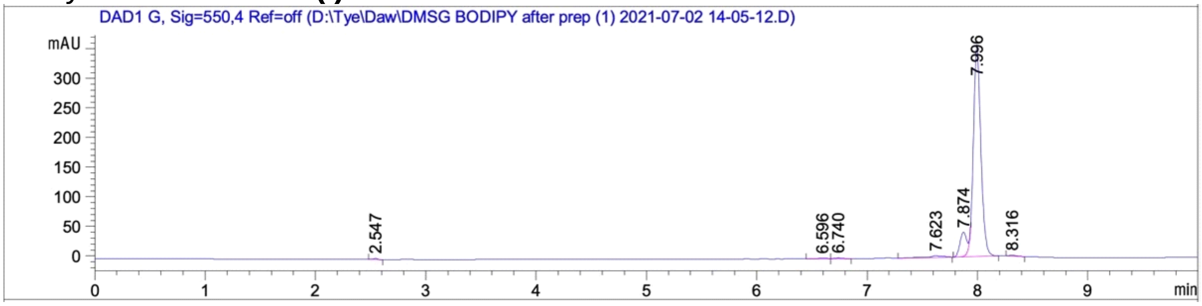

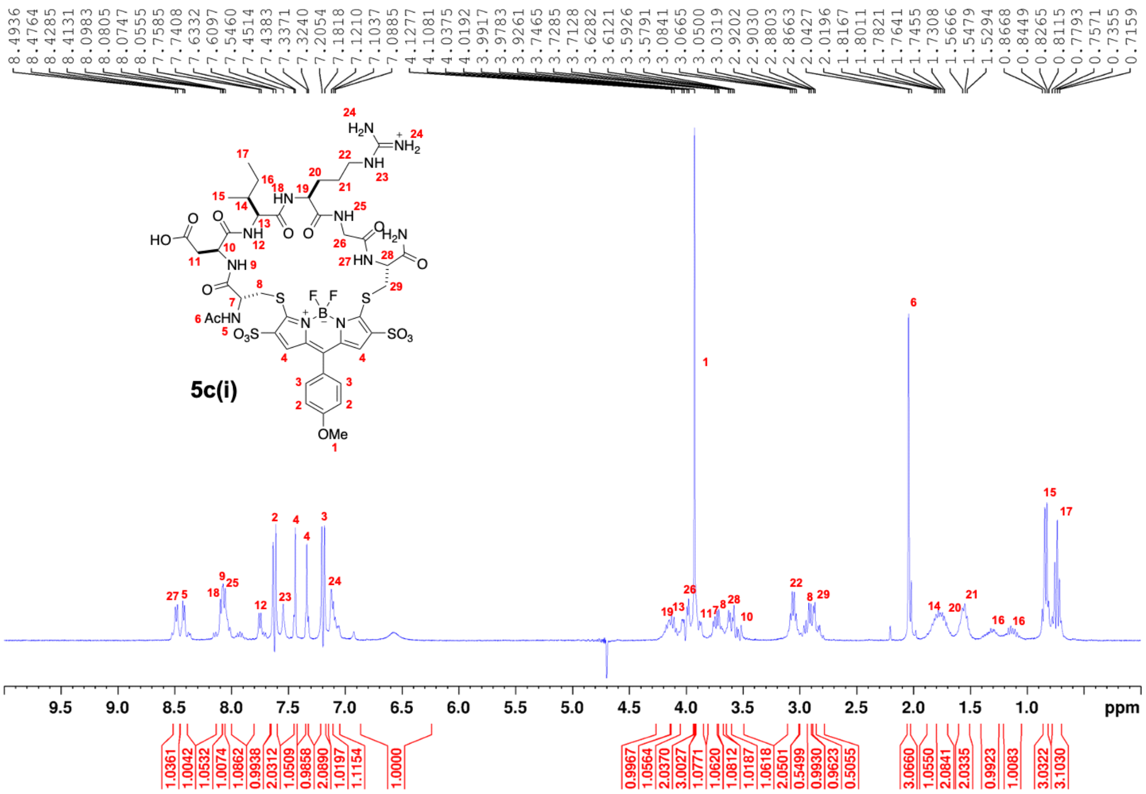

^{1}H NMR of 5c(i)

5c(i)

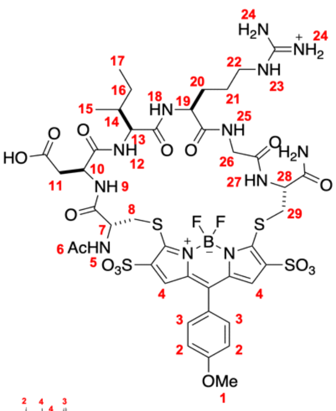

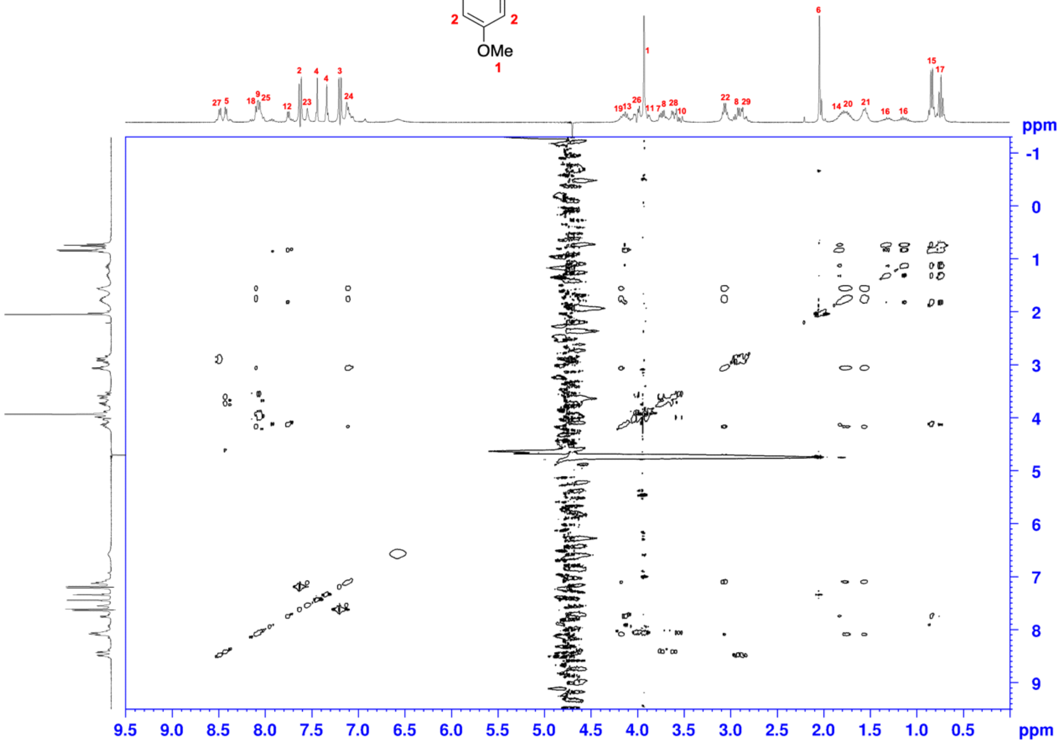

TOCSY of 5c(i)

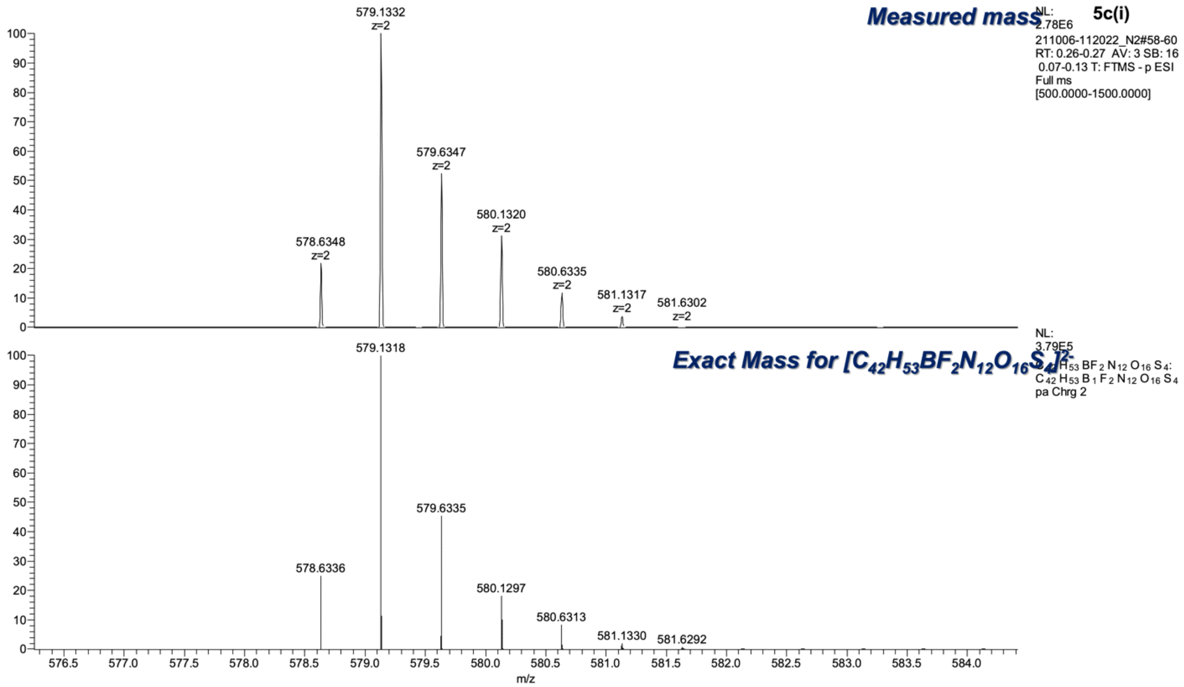

HRMS of 5c(i)

Analytical HPLC of **5c(i)** at 280 nm detection

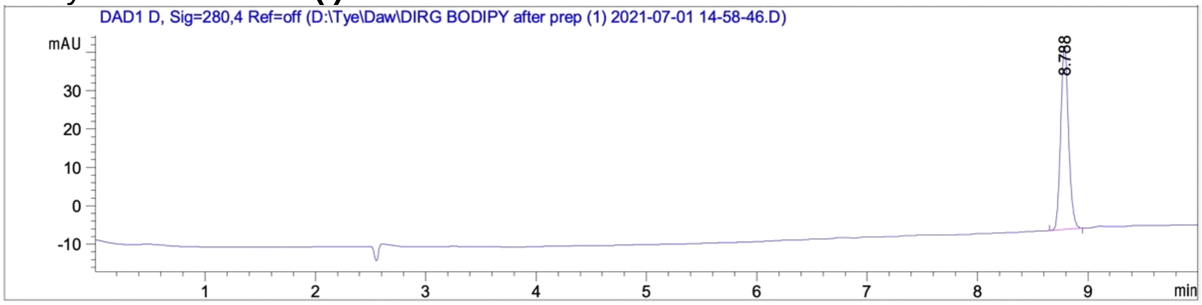

Analytical HPLC of **5c(i)** at 550 nm detection

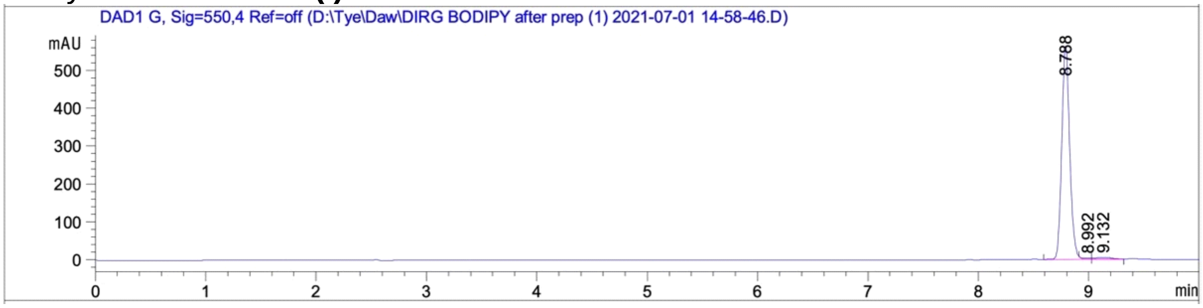

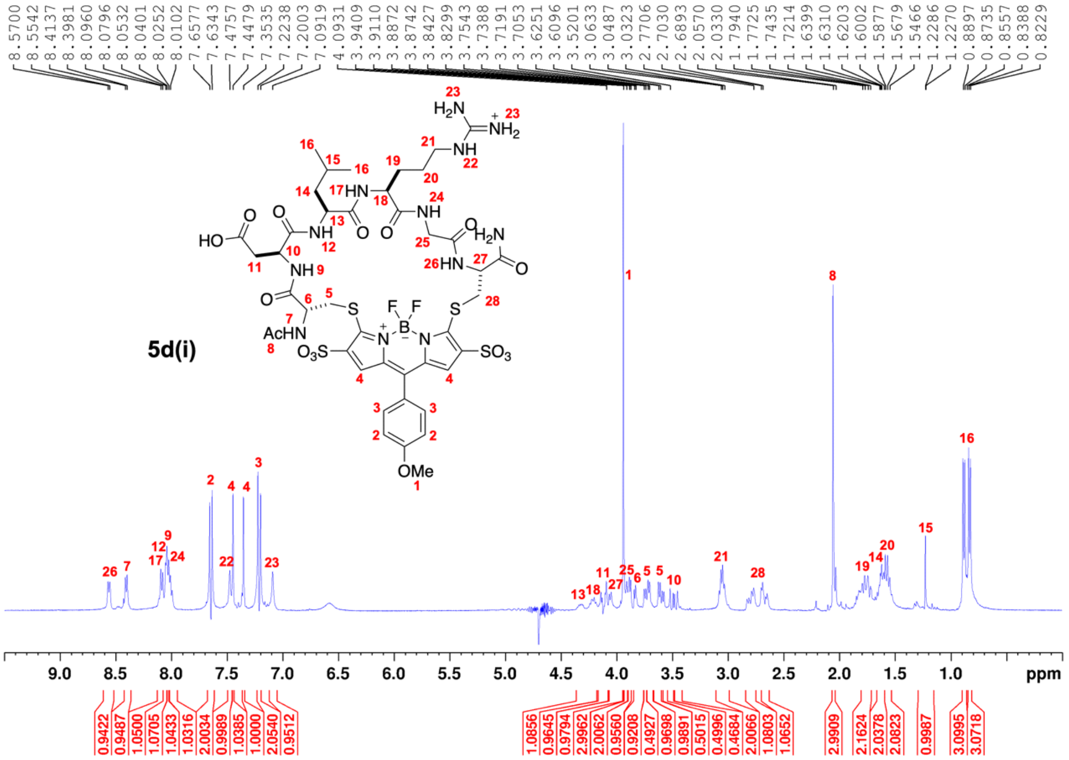

^{1}H NMR of 5d(i)

5d(i)

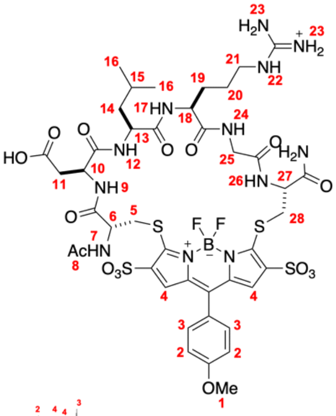

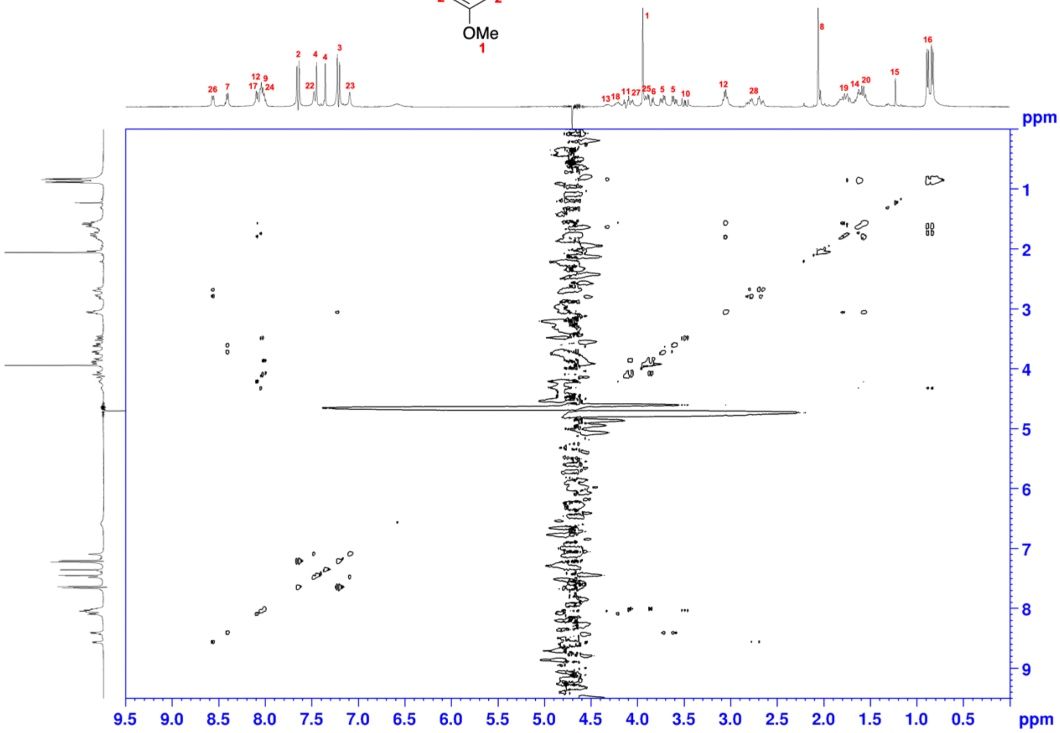

TOCSY of 5d(i)

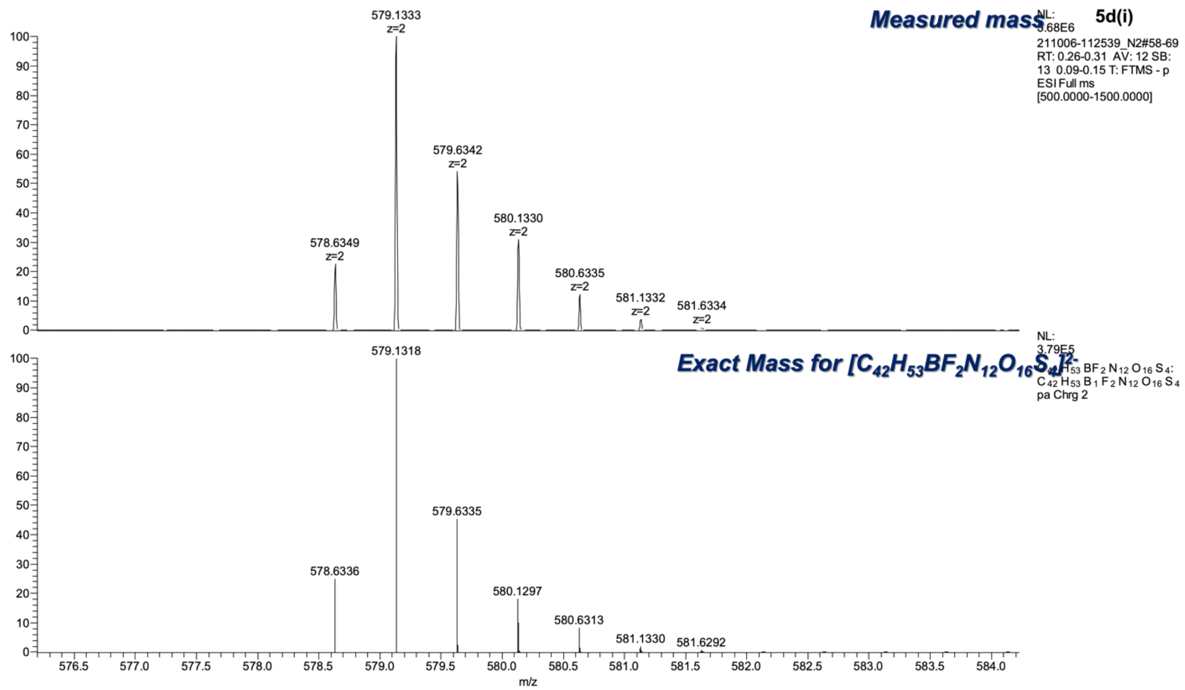

HRMS of 5d(i)

Analytical HPLC of **5d(i)** at 280 nm detection

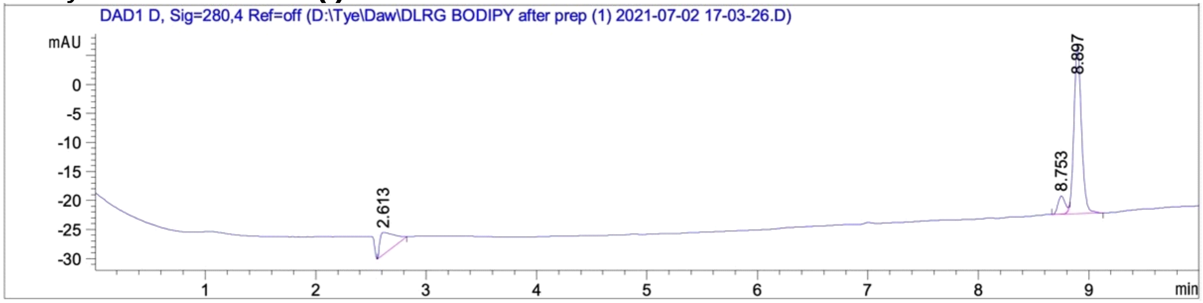

Analytical HPLC of **5d(i)** at 550 nm detection

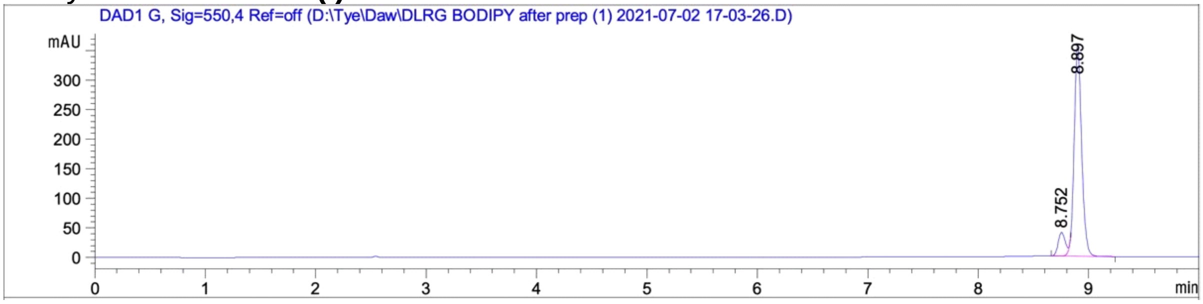

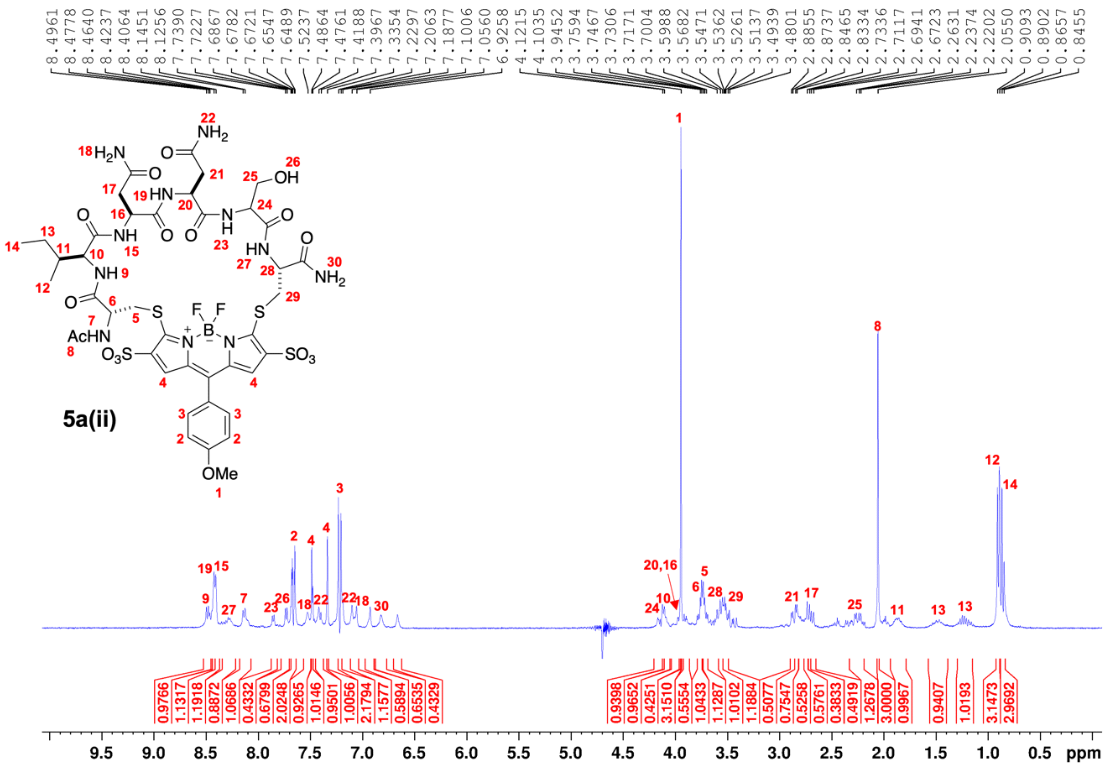

^{1}H NMR of 5a(ii)

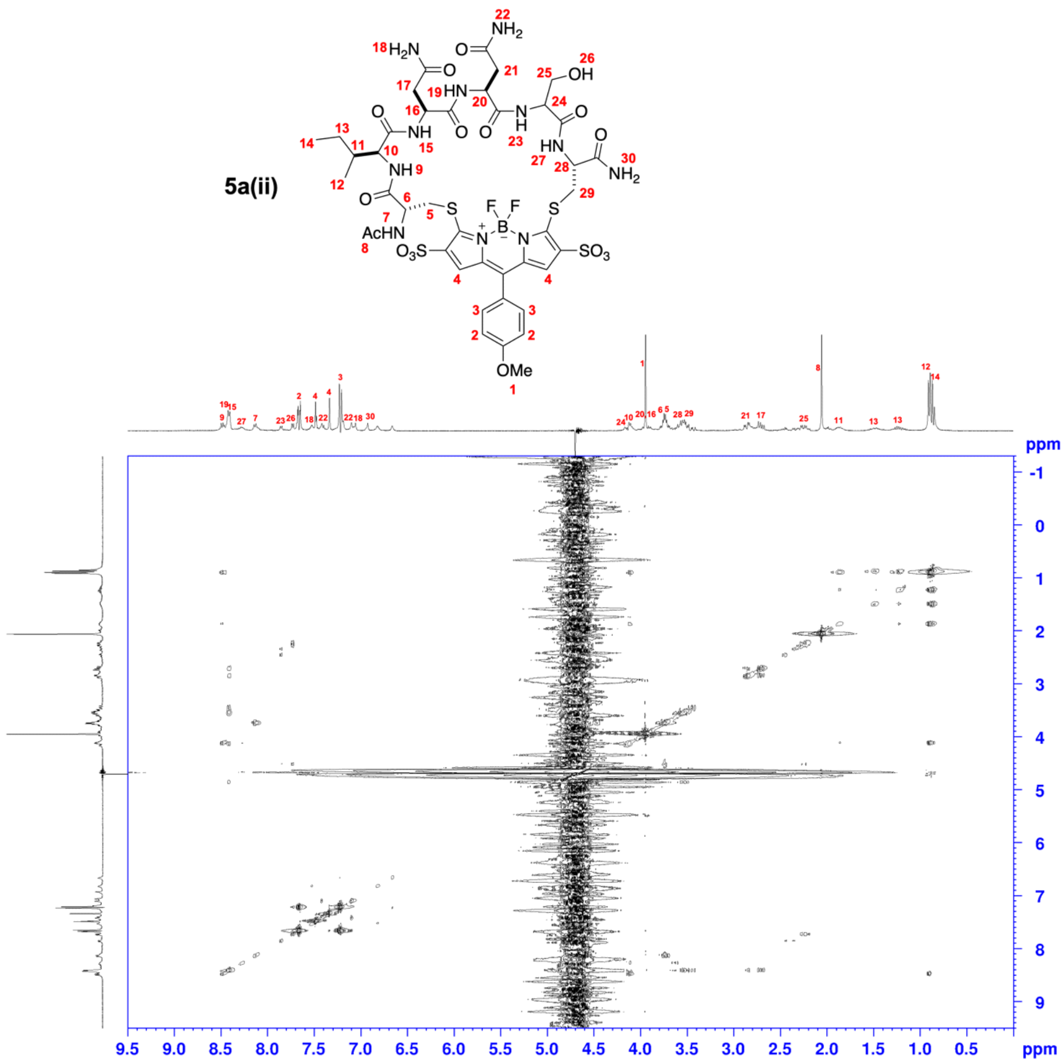

TOCSY of 5a(ii)

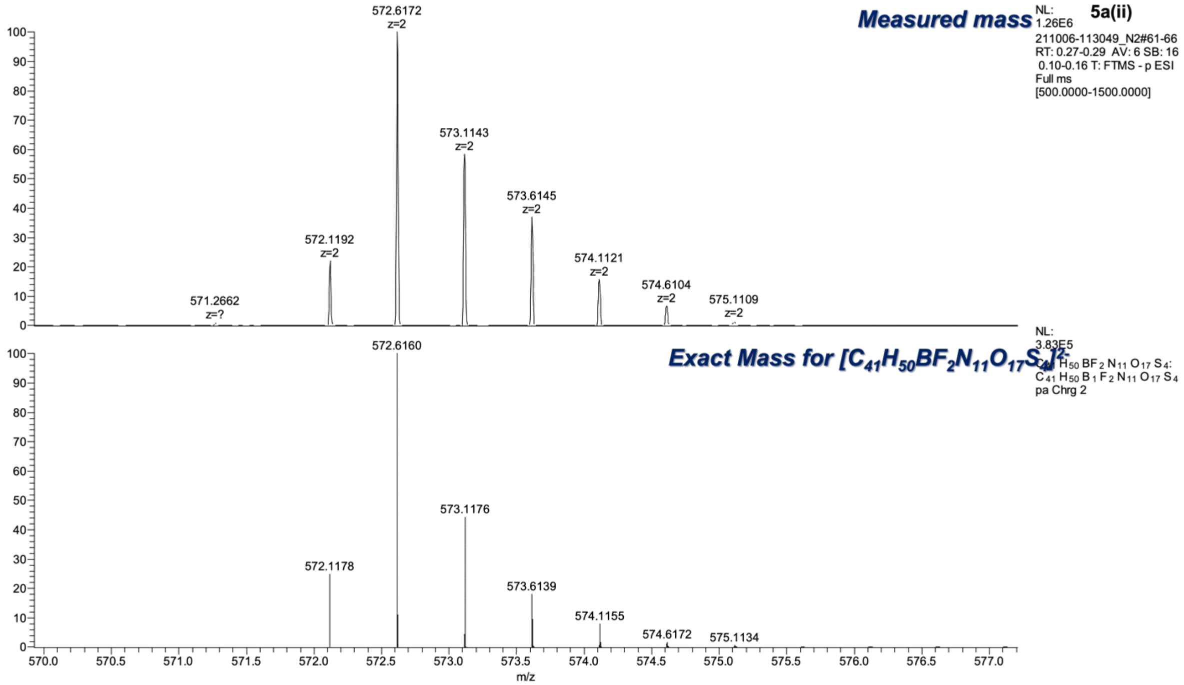

HRMS of 5a(ii)

Analytical HPLC of **5a(ii)** at 280 nm detection

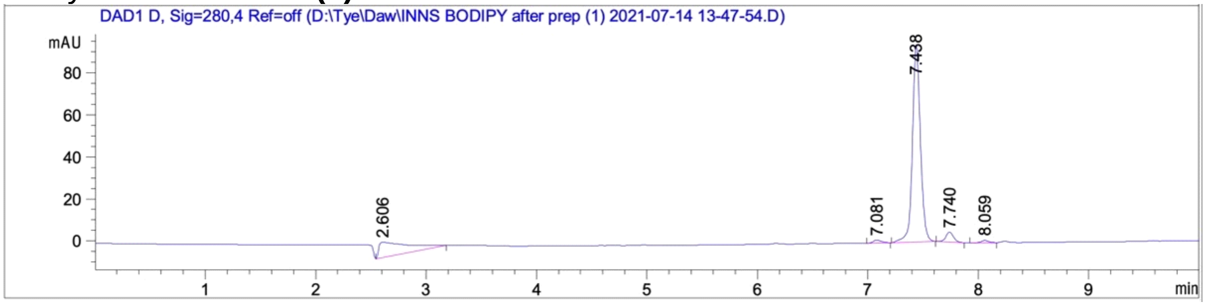

Analytical HPLC of **5a(ii)** at 550 nm detection

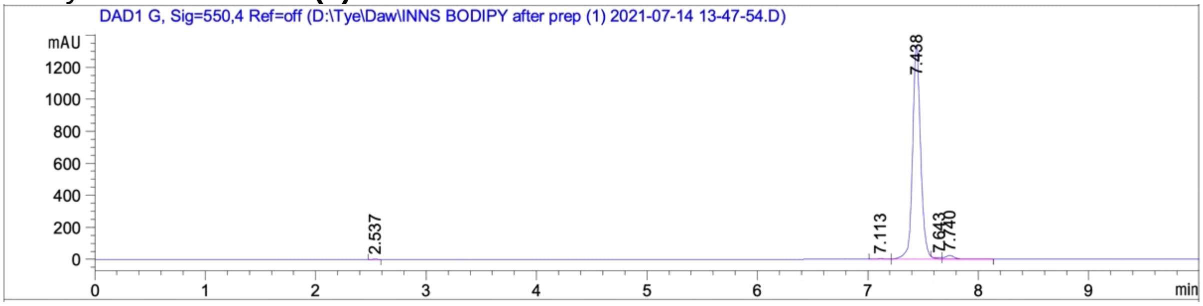

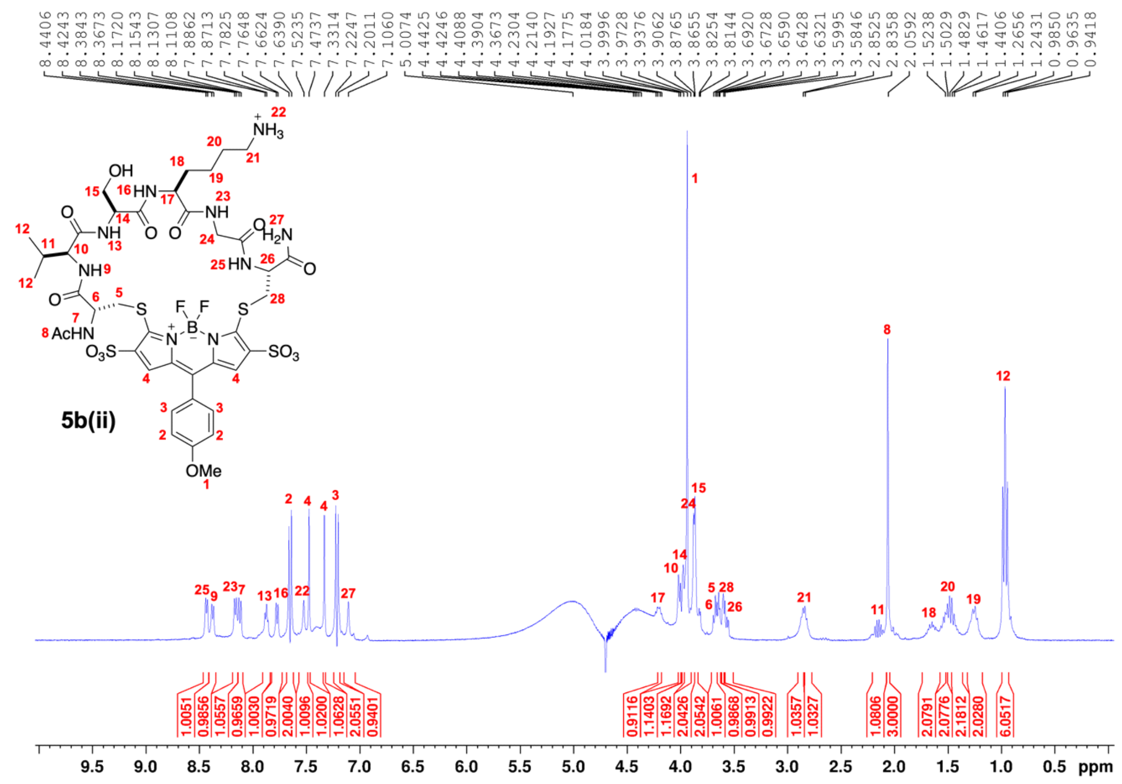

^{1}H NMR of 5b(ii)

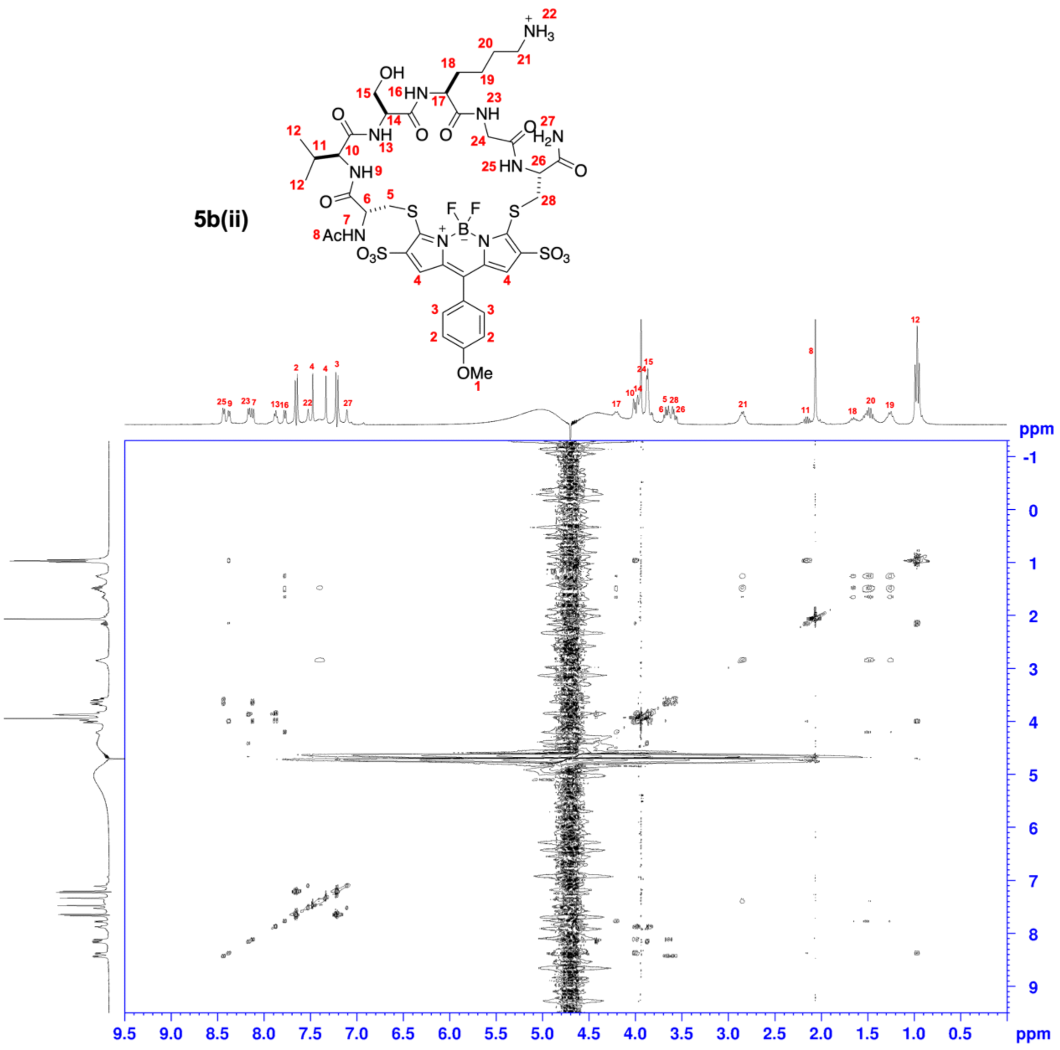

TOCSY of 5b(ii)

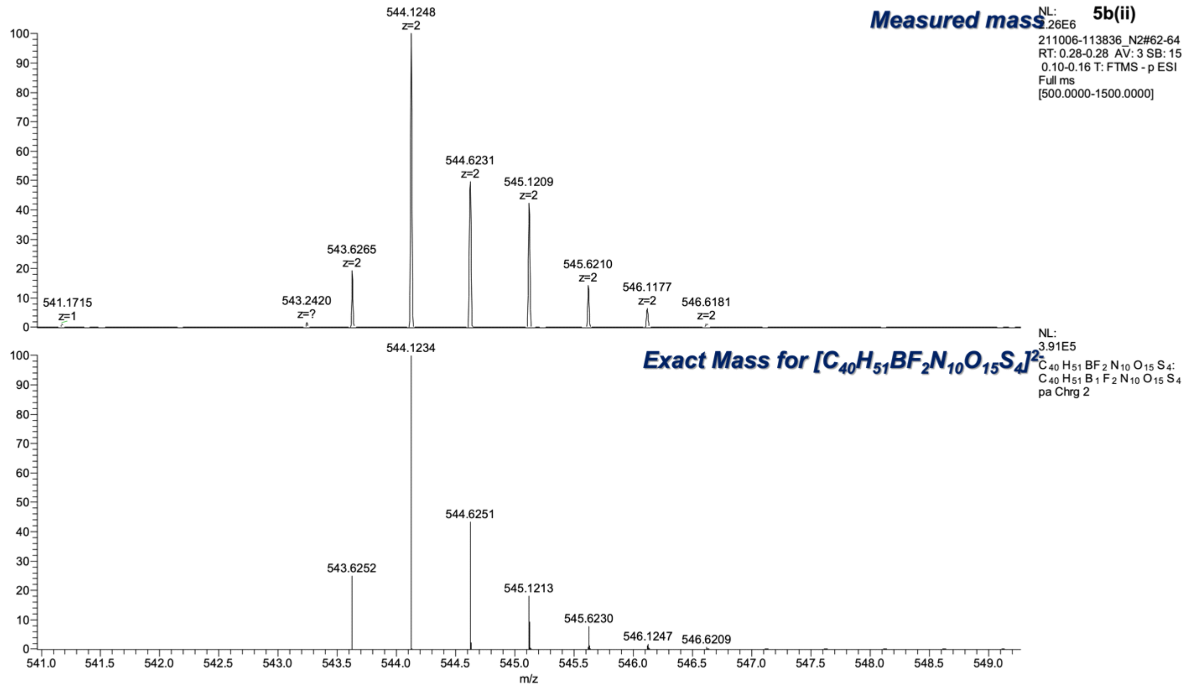

HRMS of 5b(ii)

Analytical HPLC of **5b(ii)** at 280 nm detection

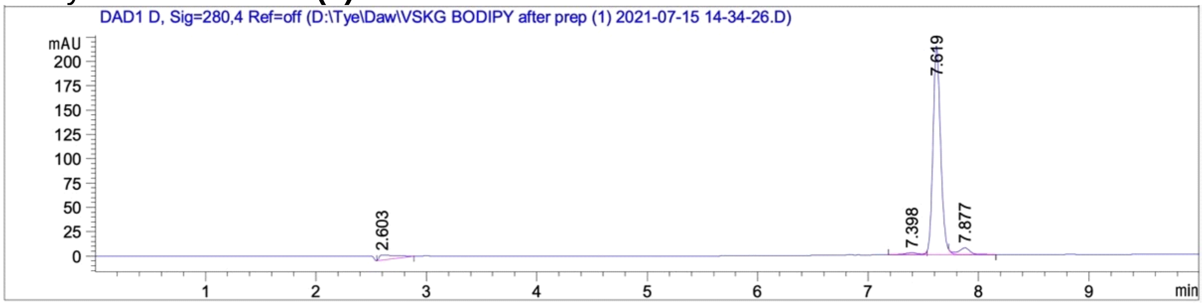

Analytical HPLC of **5b(ii)** at 550 nm detection

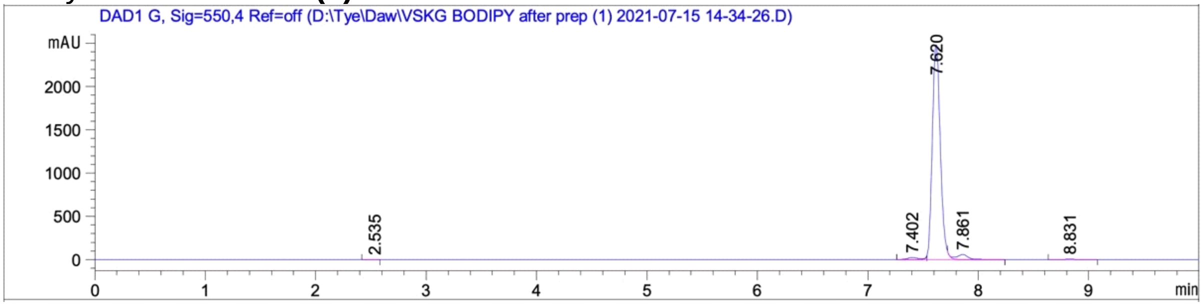

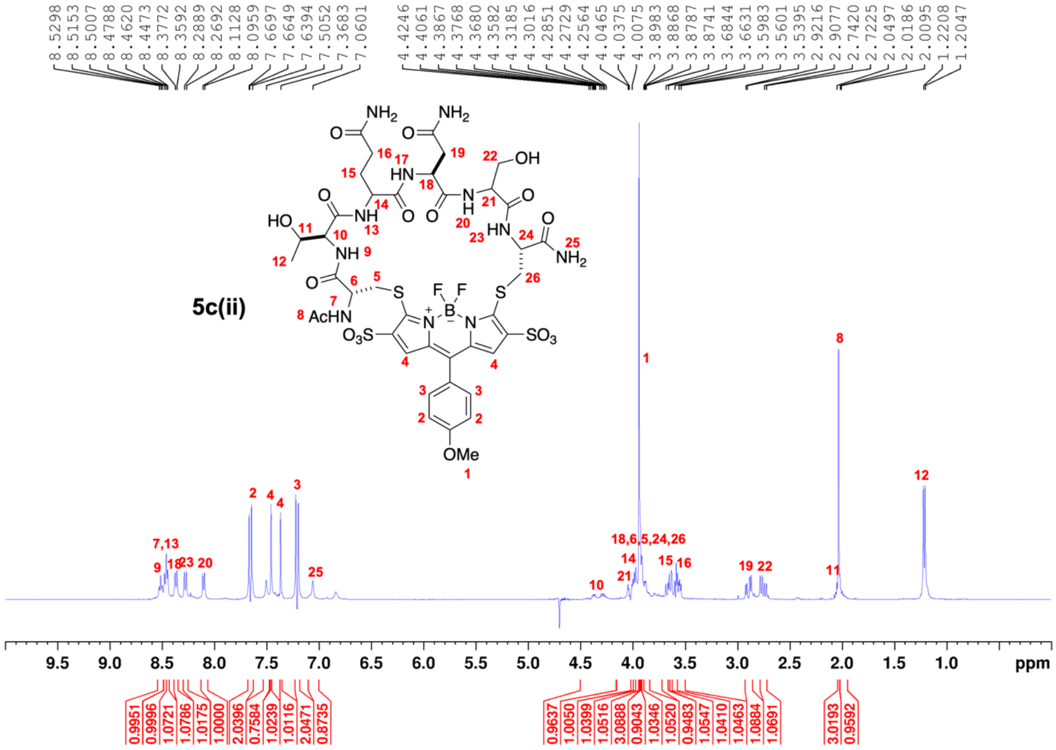

^{1}H NMR of 5c(ii)

5c(ii)

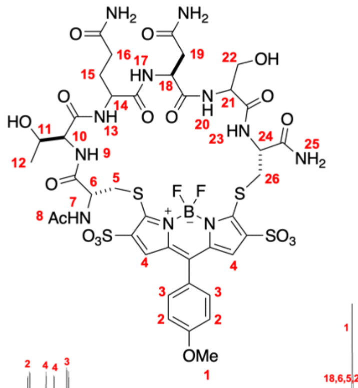

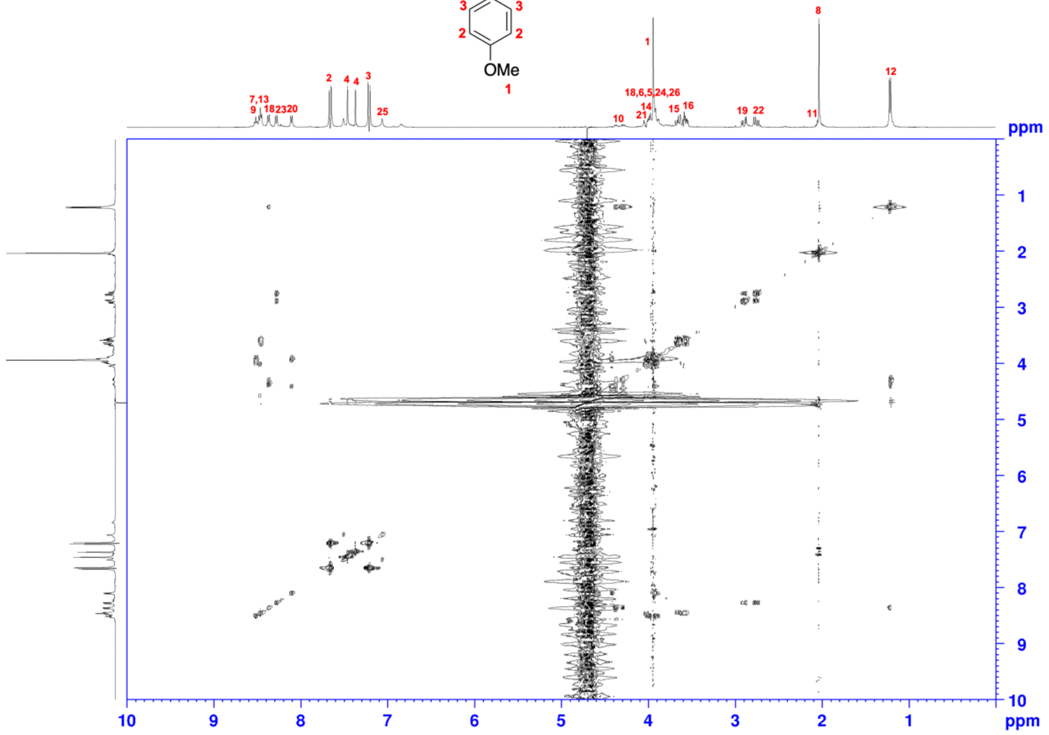

TOCSY of 5c(ii)

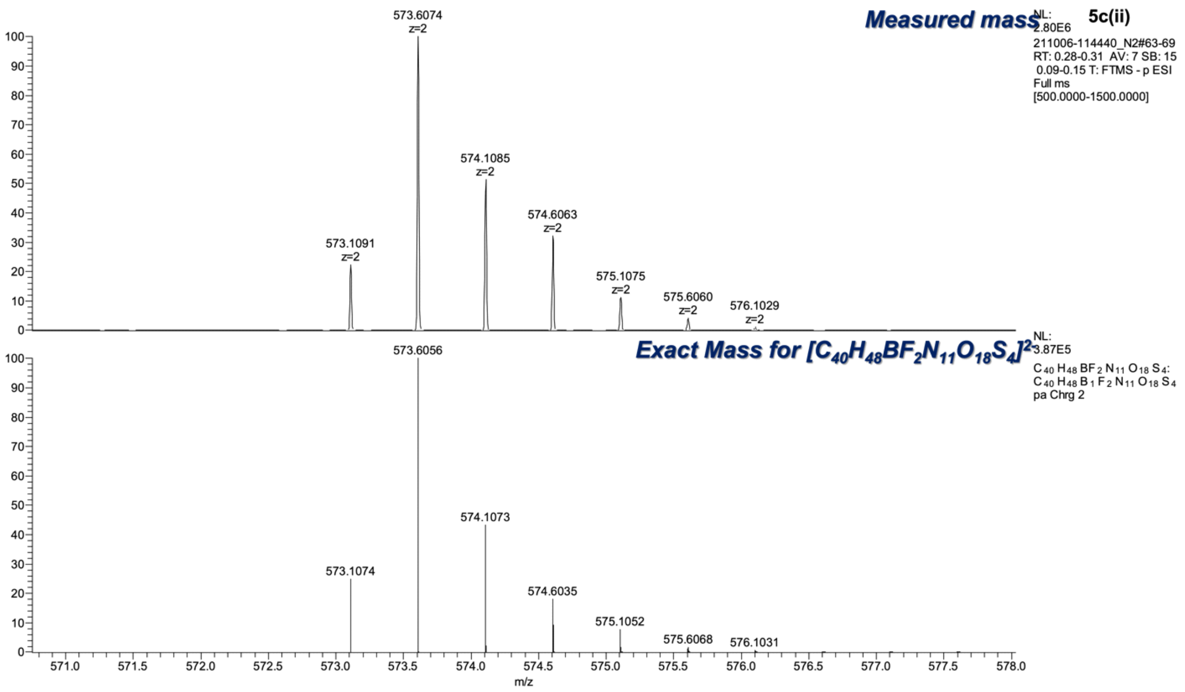

HRMS of 5c(ii)

Analytical HPLC of **5c(ii)** at 280 nm detection

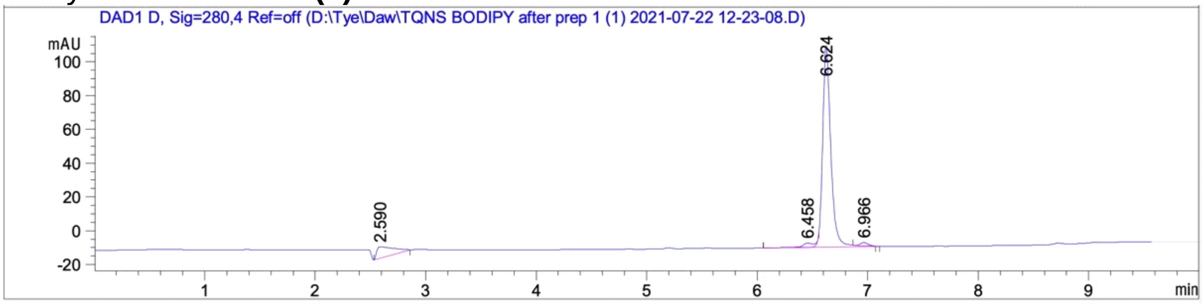

Analytical HPLC of **5c(ii)** at 550 nm detection

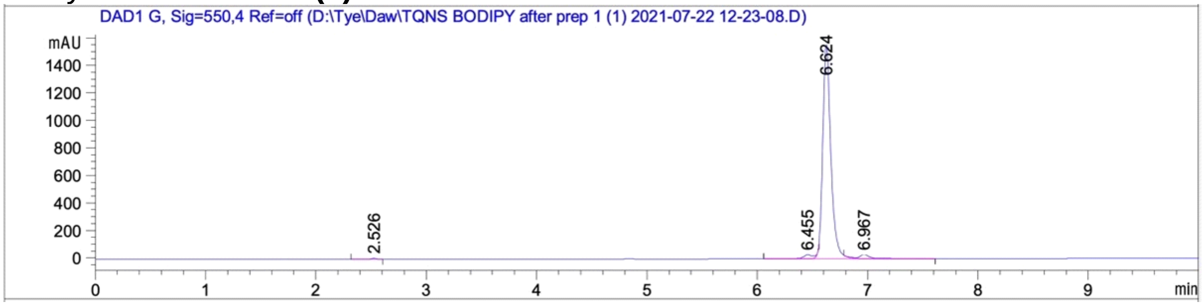

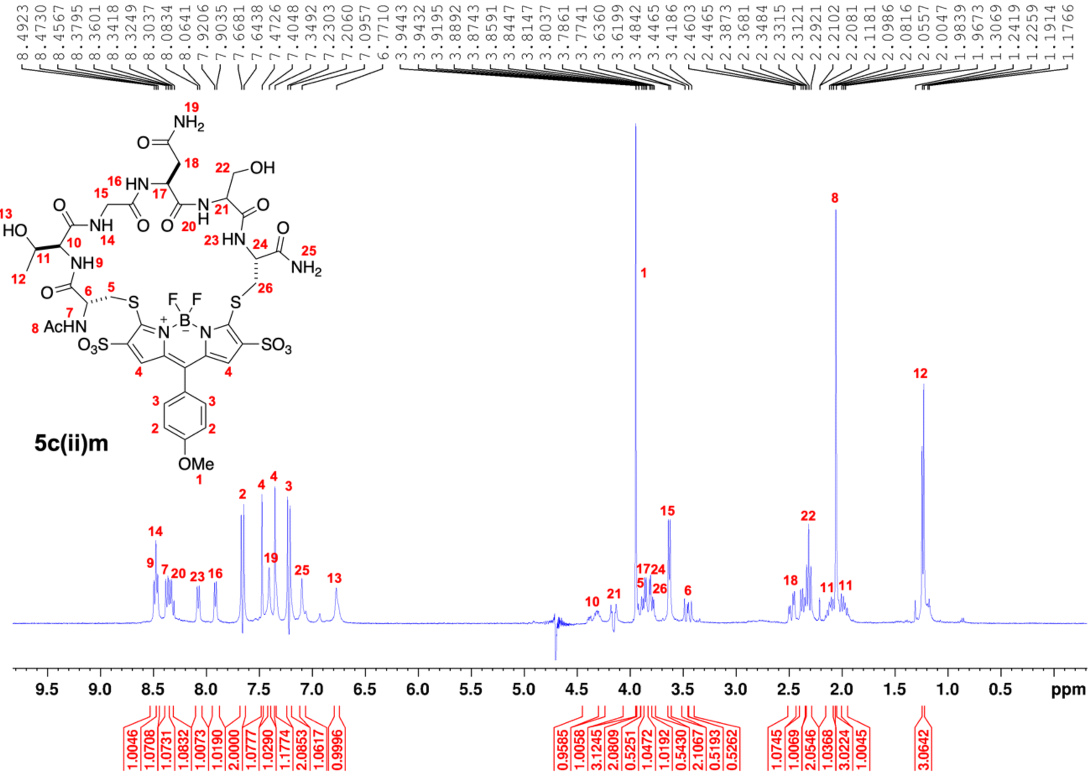

^{1}H NMR of 5c(ii)m

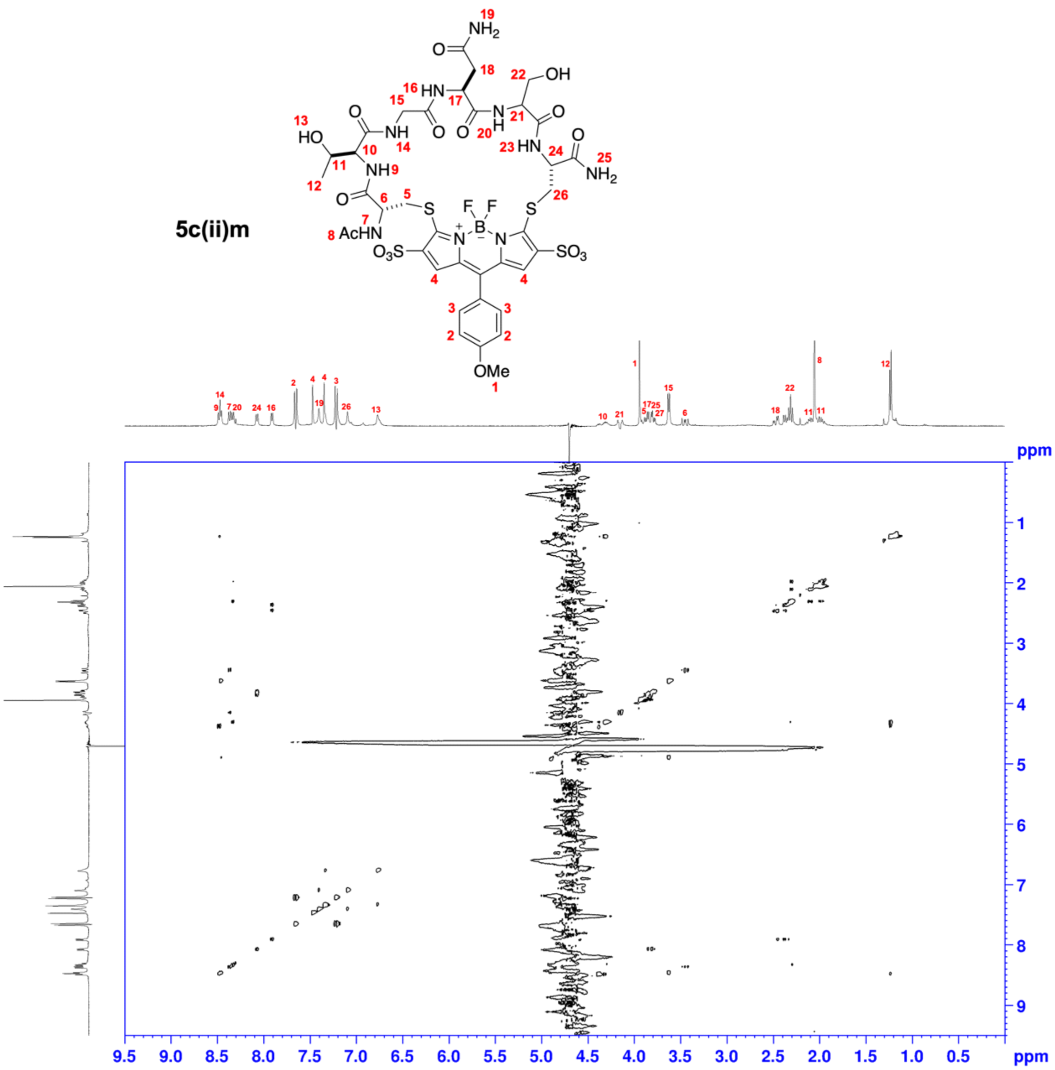

TOCSY of 5c(ii)m

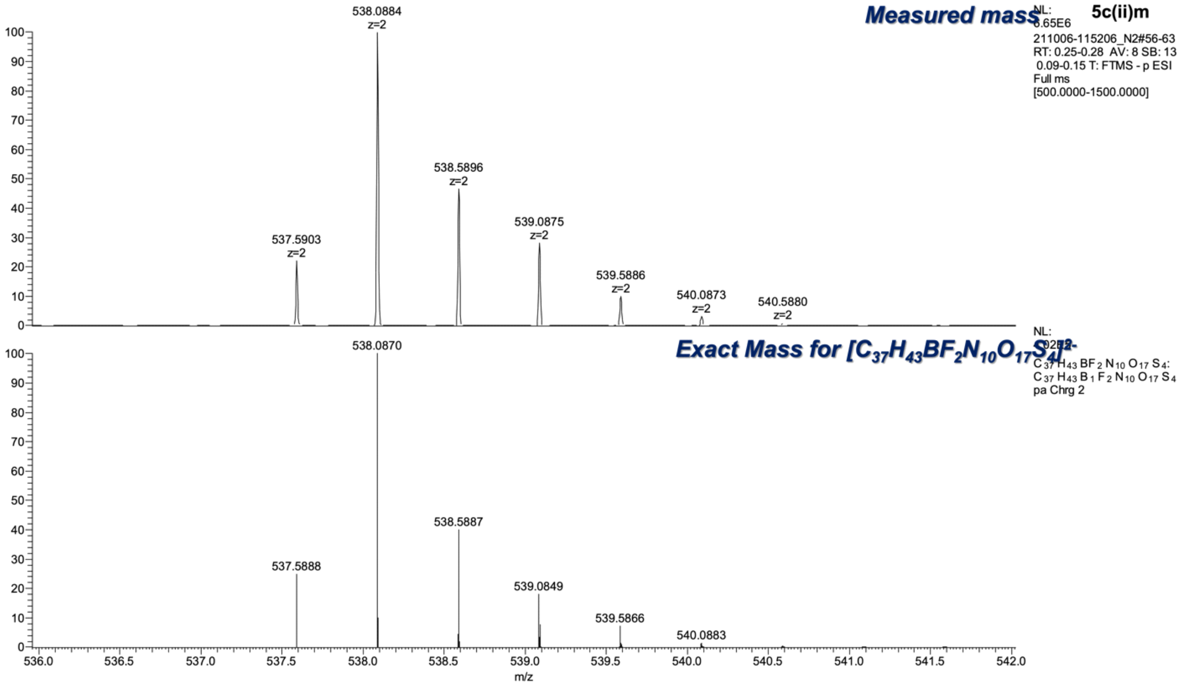

HRMS of 5c(ii)m

Analytical HPLC of **5c(ii)m** at 280 nm detection

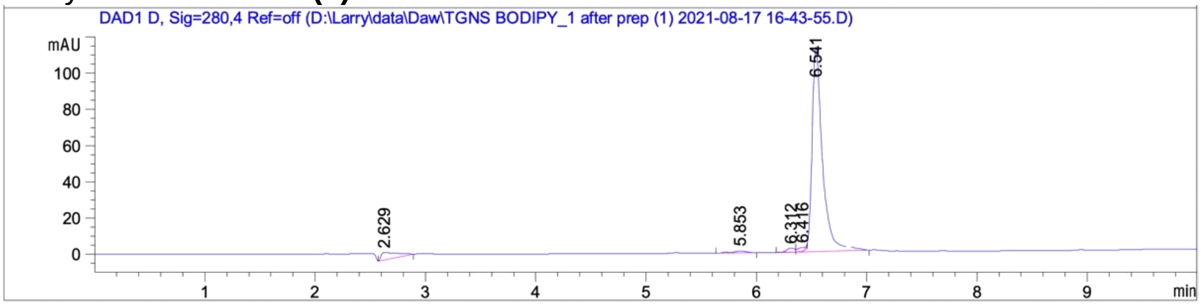

Analytical HPLC of **5c(ii)m** at 550 nm detection

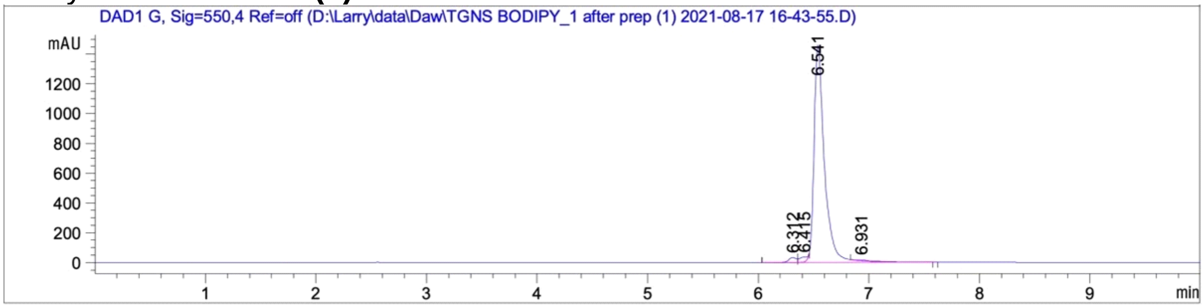

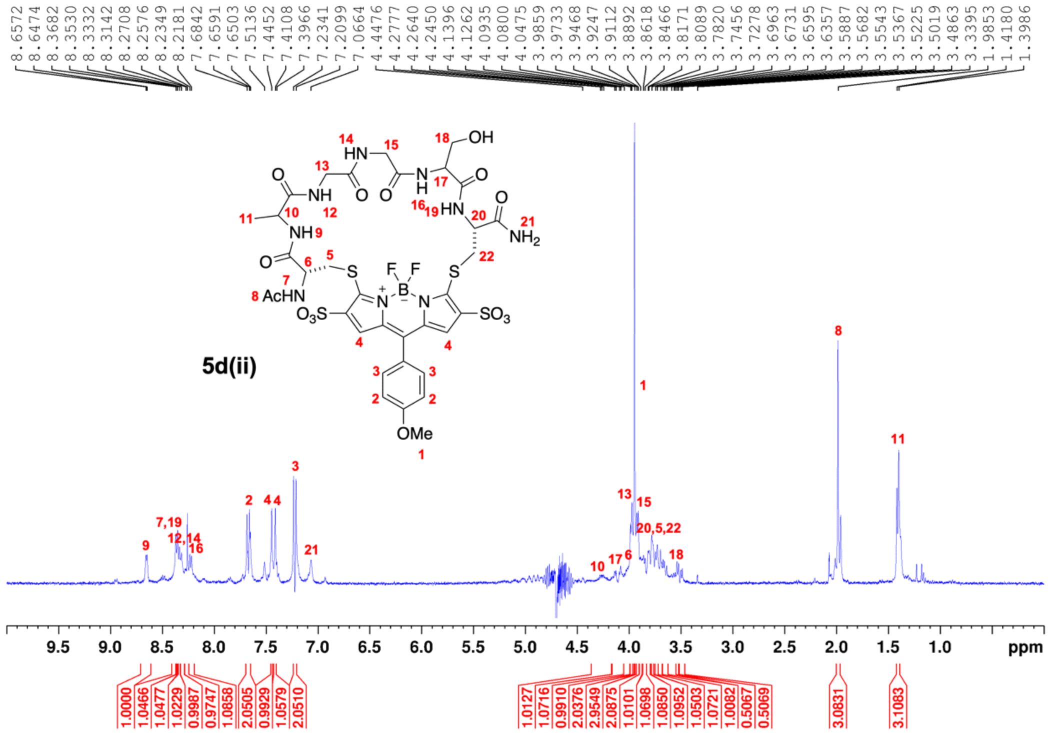

^{1}H NMR of 5d(ii)

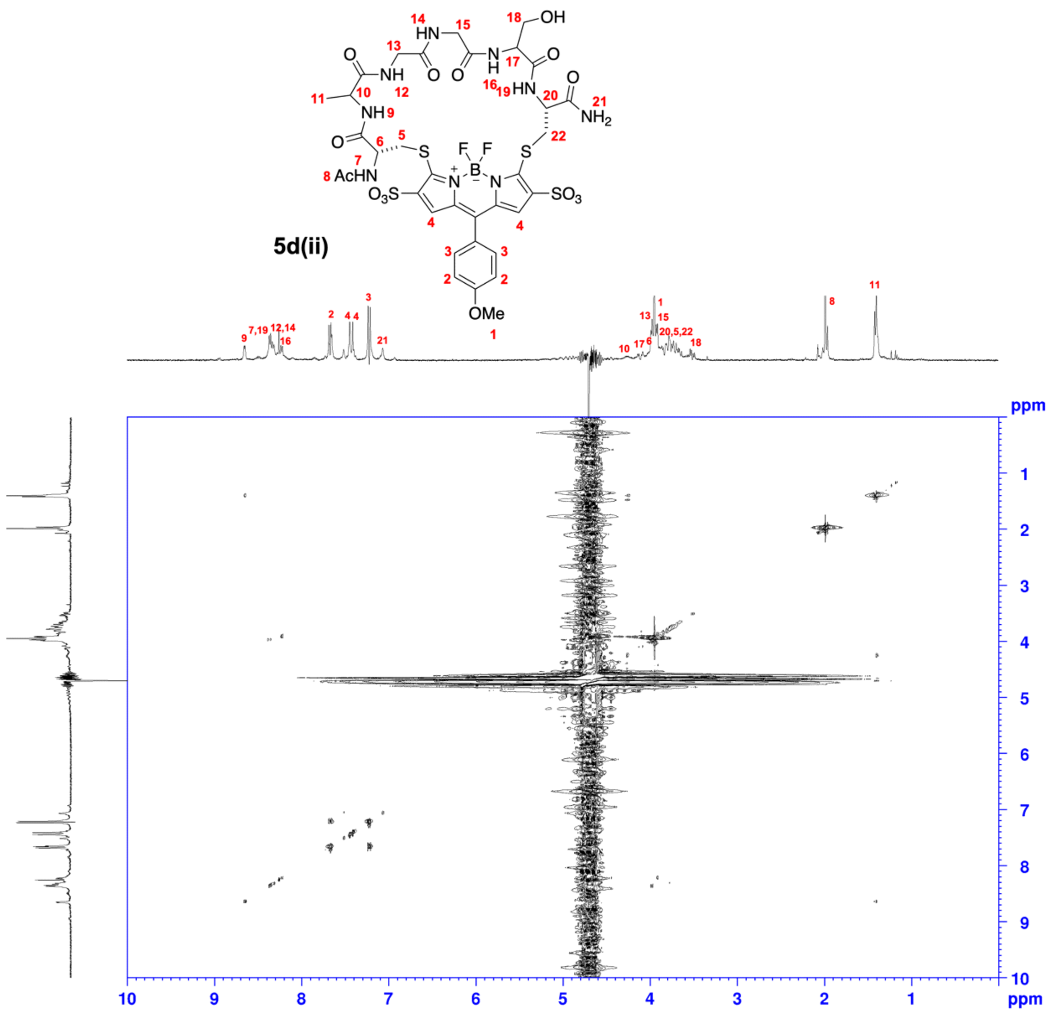

TOCSY of 5d(ii)

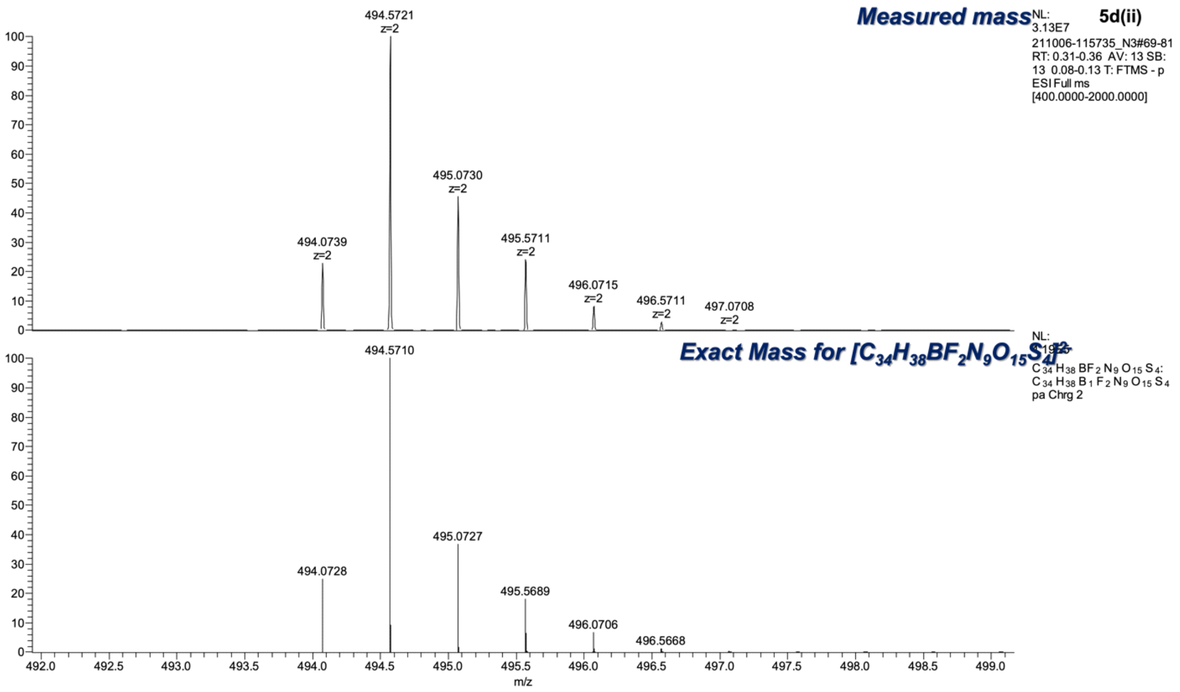

HRMS of 5d(ii)

Analytical HPLC of **5d(ii)** at 280 nm detection

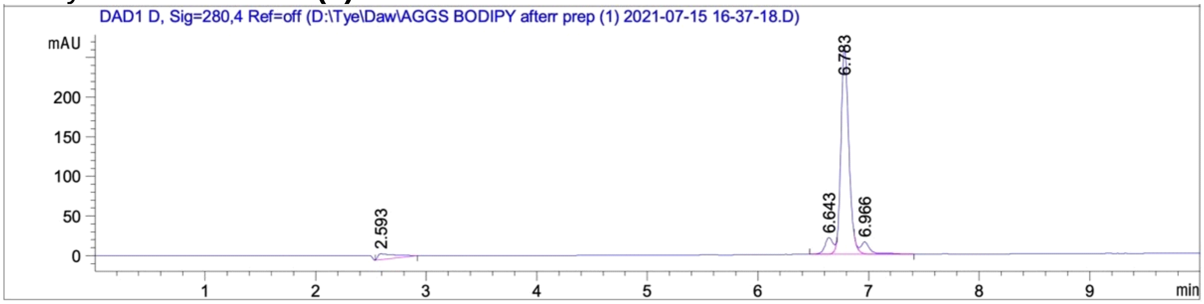

Analytical HPLC of **5d(ii)** at 550 nm detection

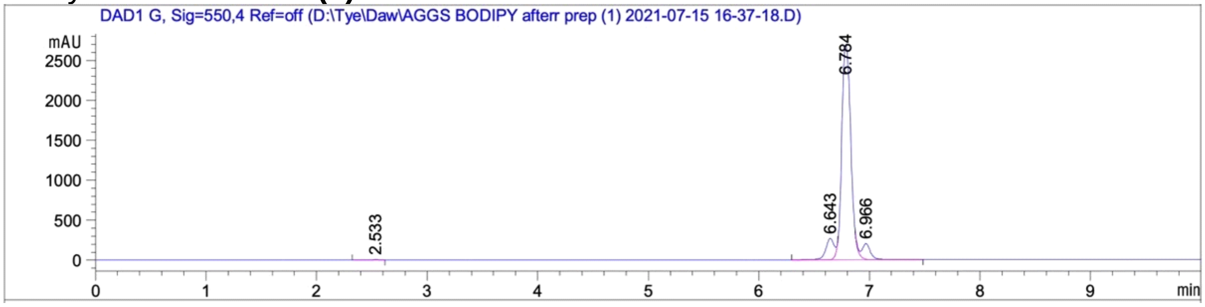

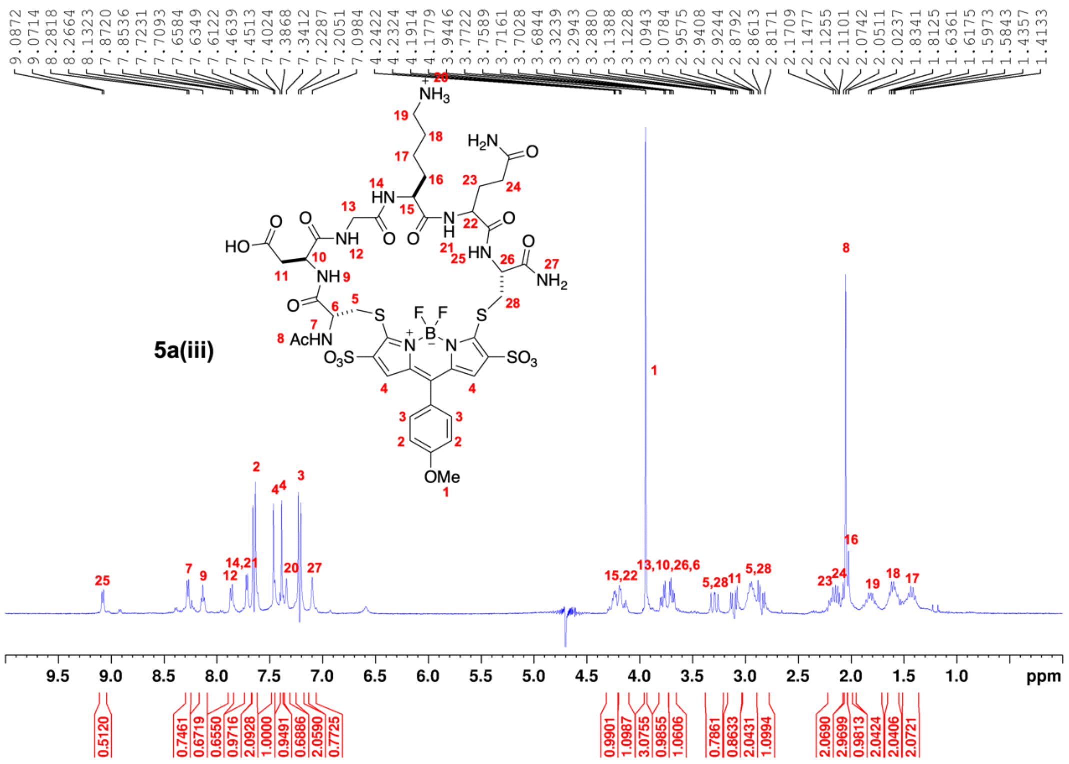

^{1}H NMR of 5a(iii)

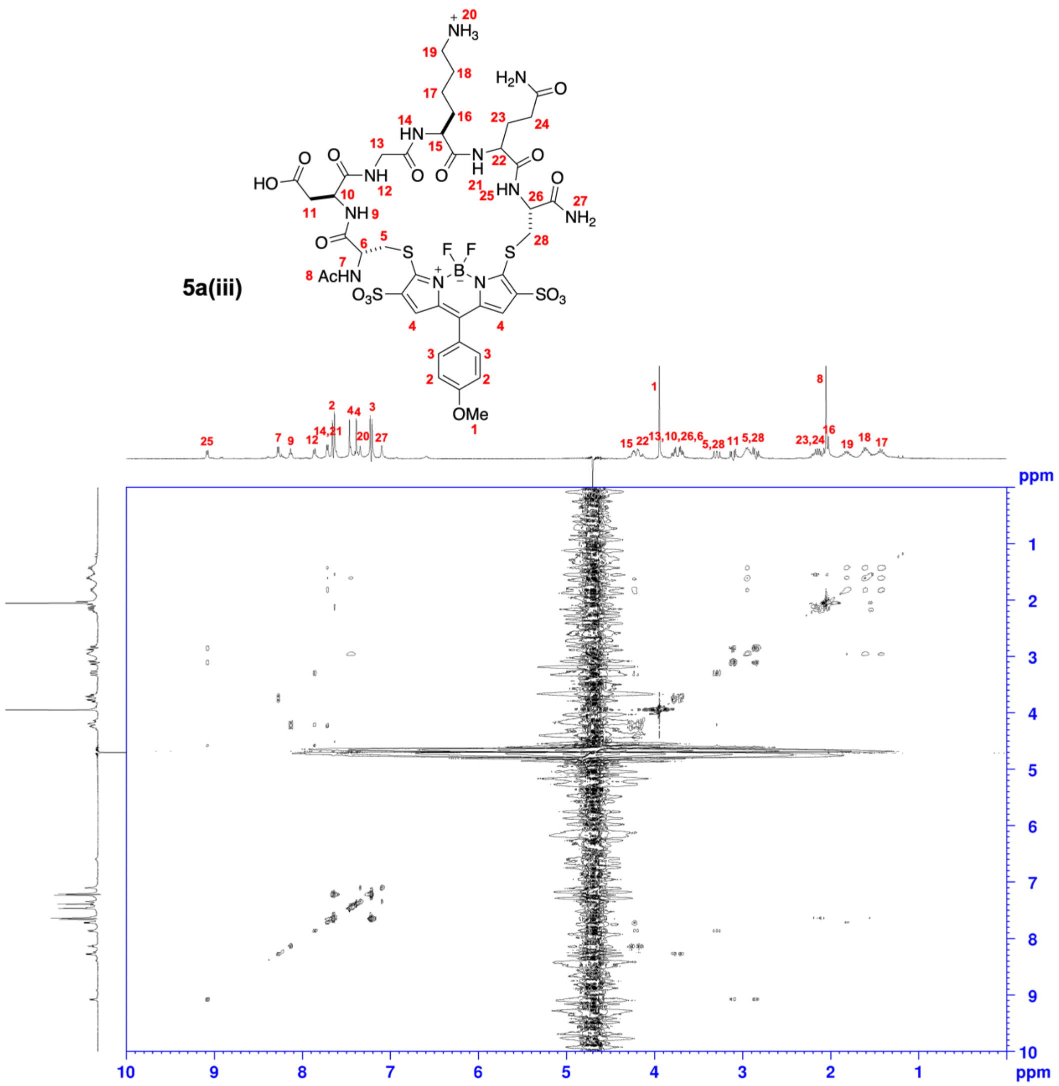

TOCSY of 5a(iii)

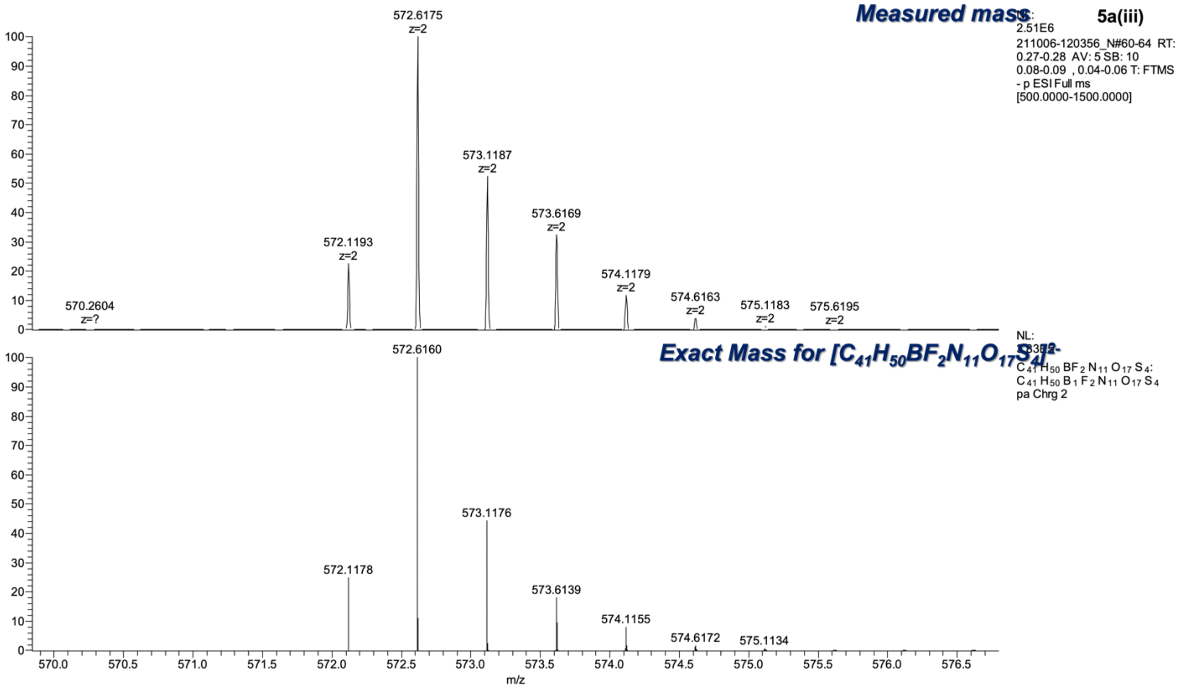

HRMS of 5a(iii)

Analytical HPLC of **5a(iii)** at 280 nm detection

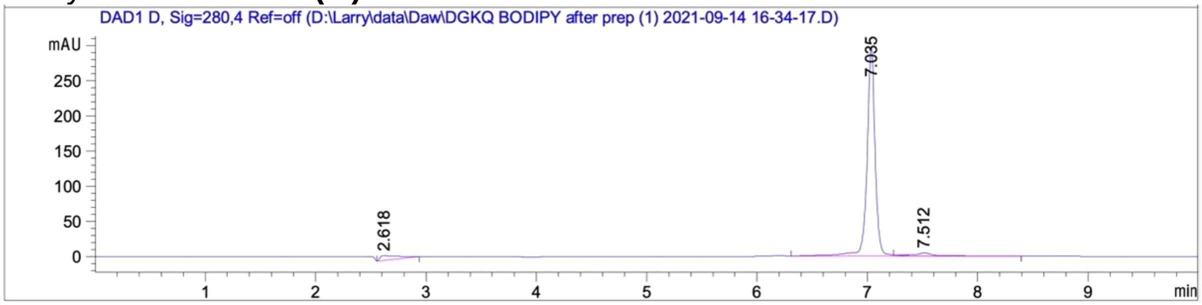

Analytical HPLC of **5a(iii)** at 550 nm detection

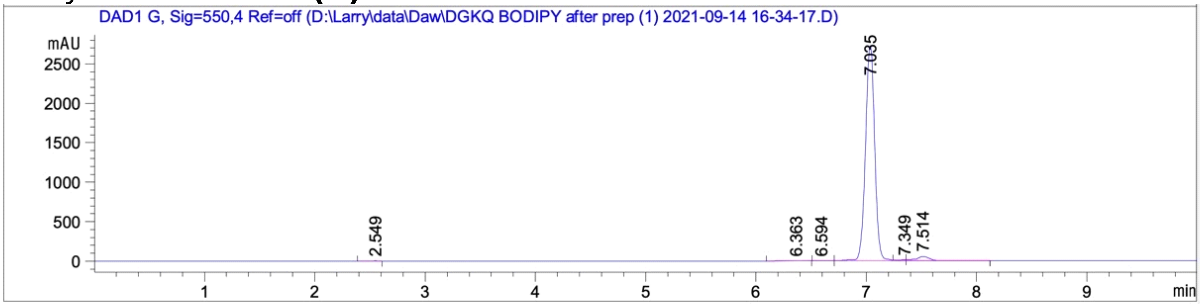

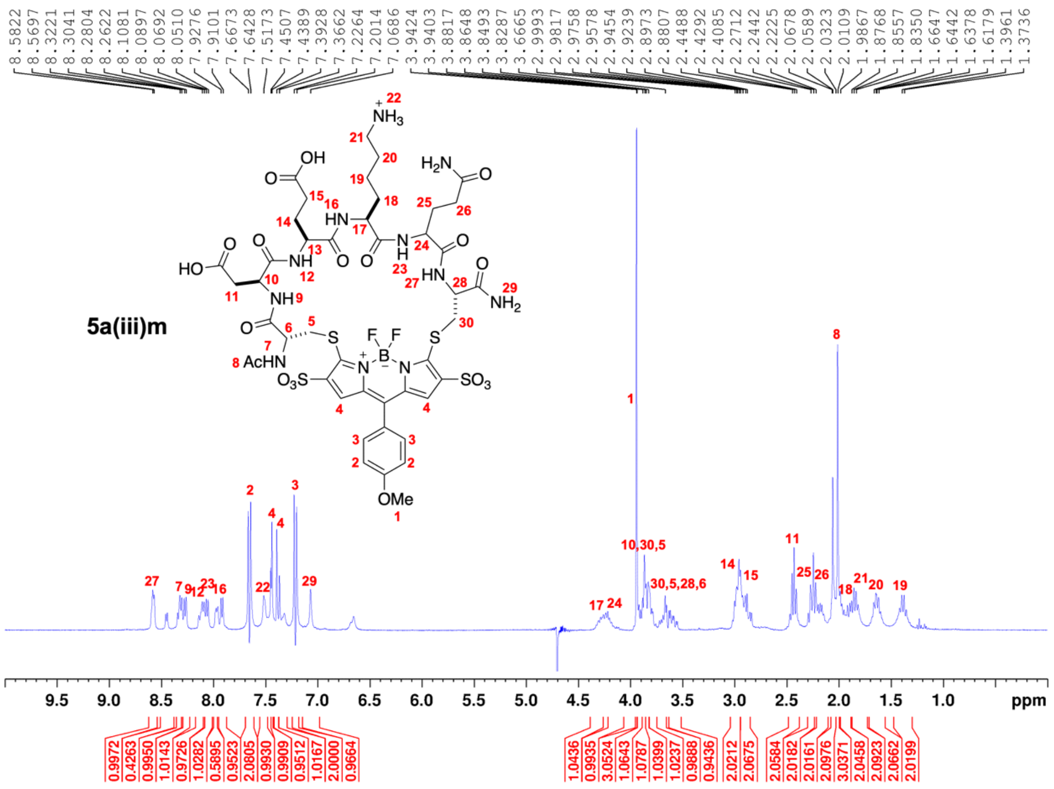

^{1}H NMR of 5a(iii)m

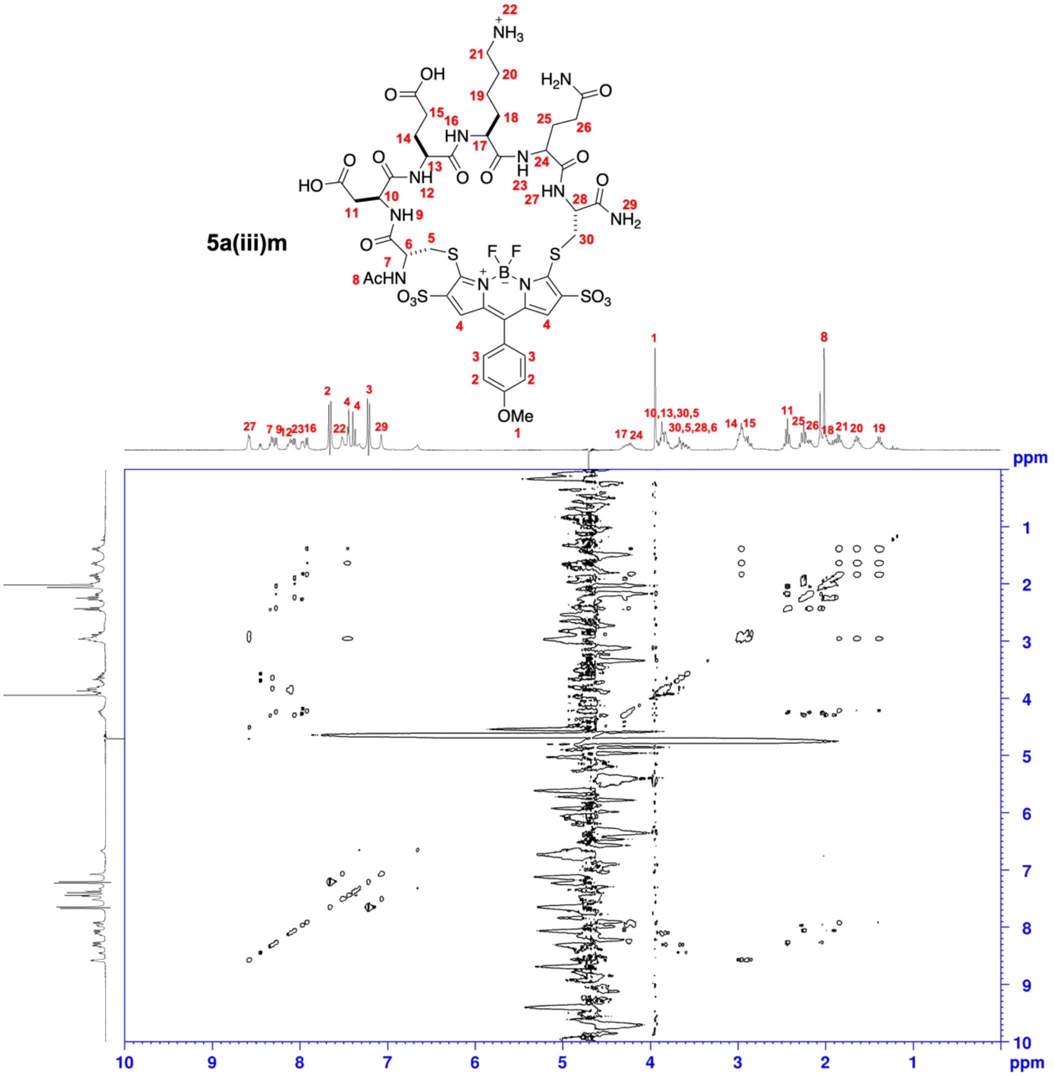

TOCSY of 5a(iii)m

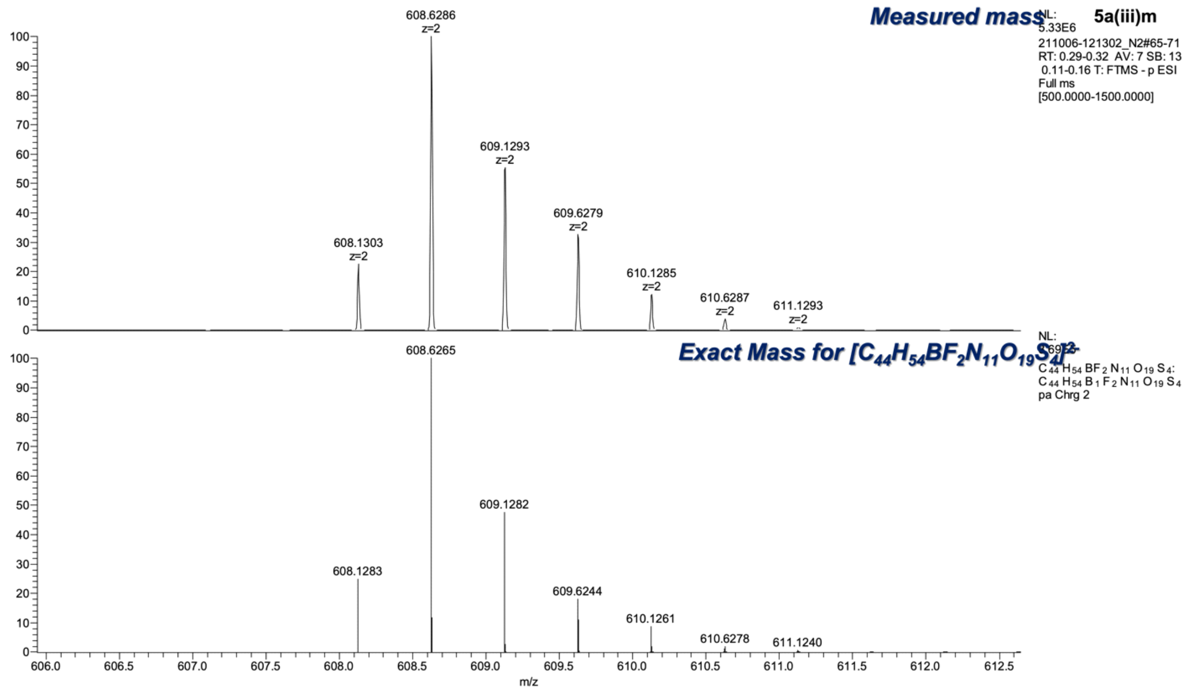

HRMS of 5a(iii)m

Analytical HPLC of **5a(iii)m** at 280 nm detection

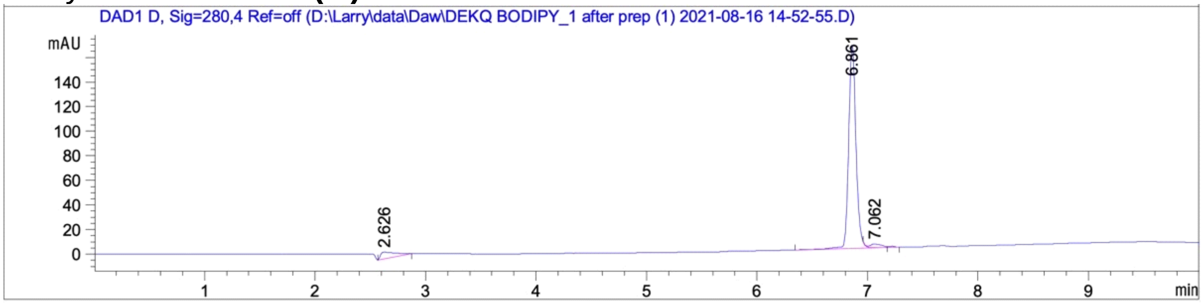

Analytical HPLC of **5a(iii)m** at 550 nm detection

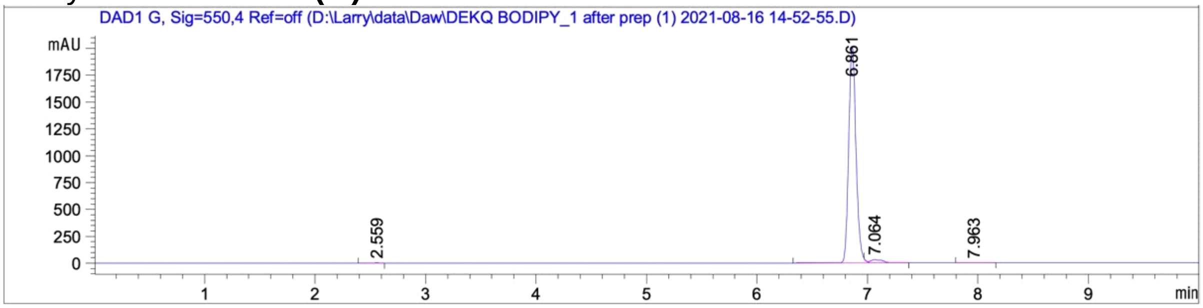

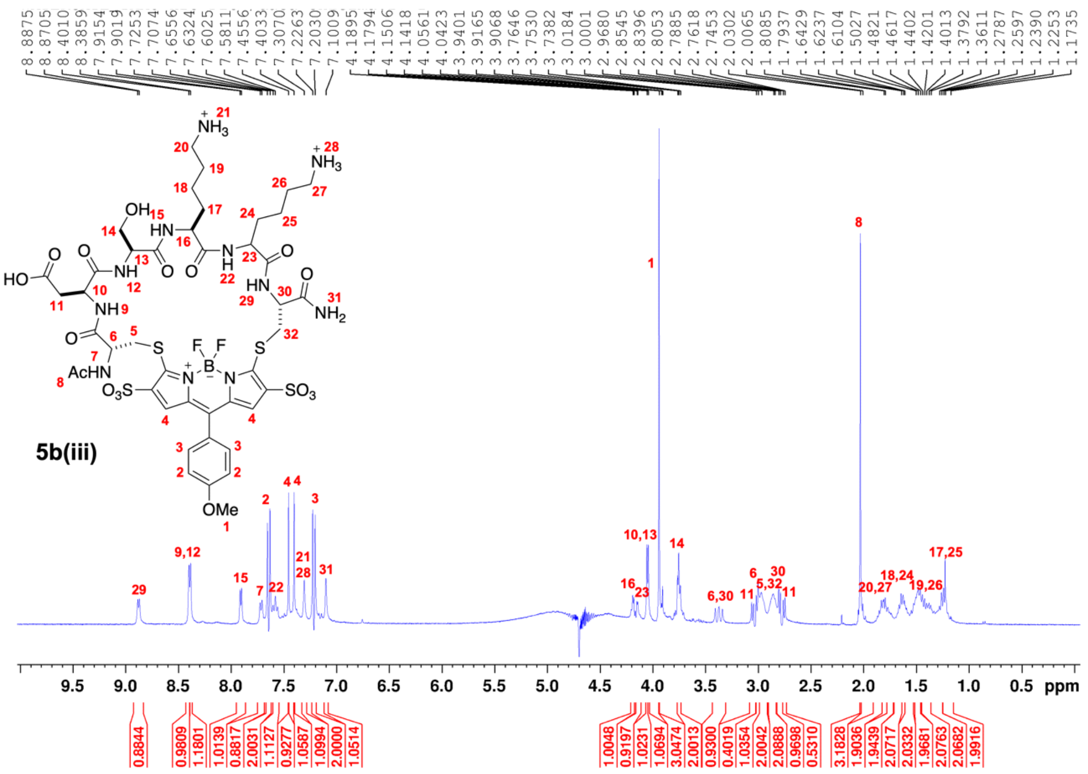

^1H NMR of 5b(iii)

5b(iii)

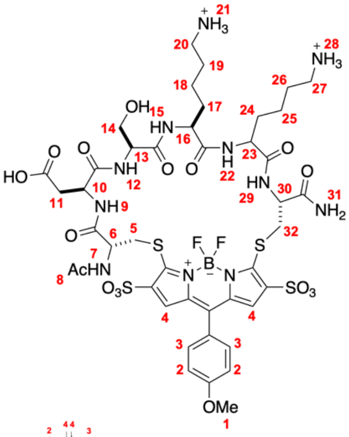

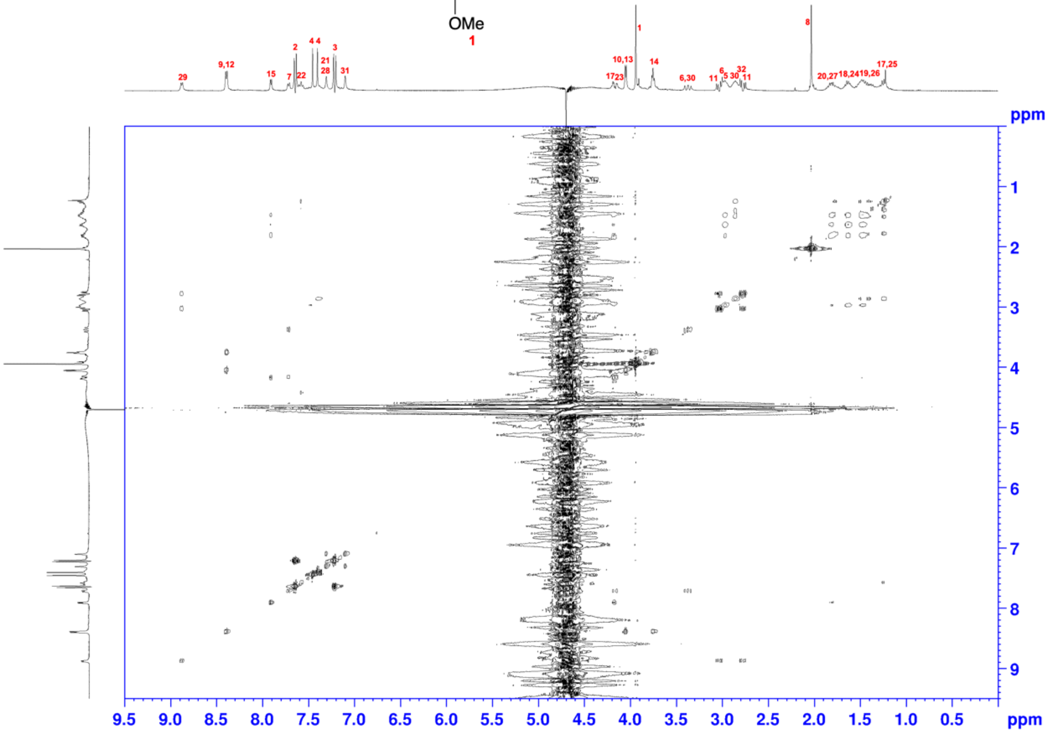

TOCSY of 5b(iii)

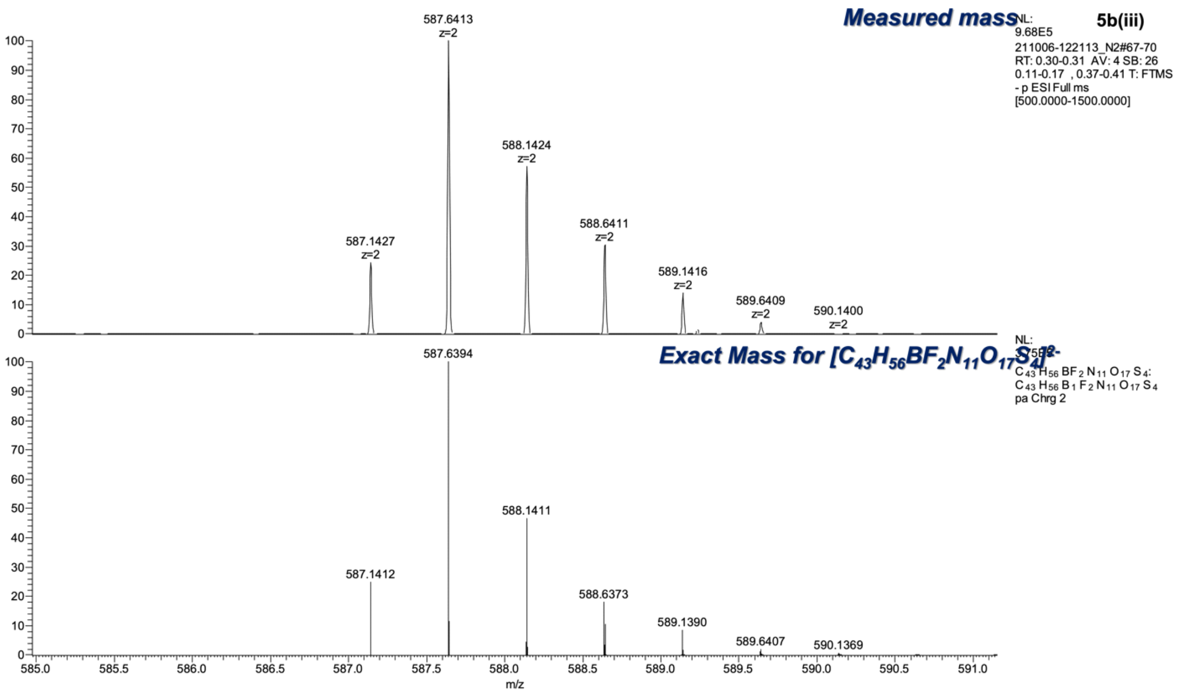

HRMS of 5b(iii)

Analytical HPLC of **5b(iii)** at 280 nm detection

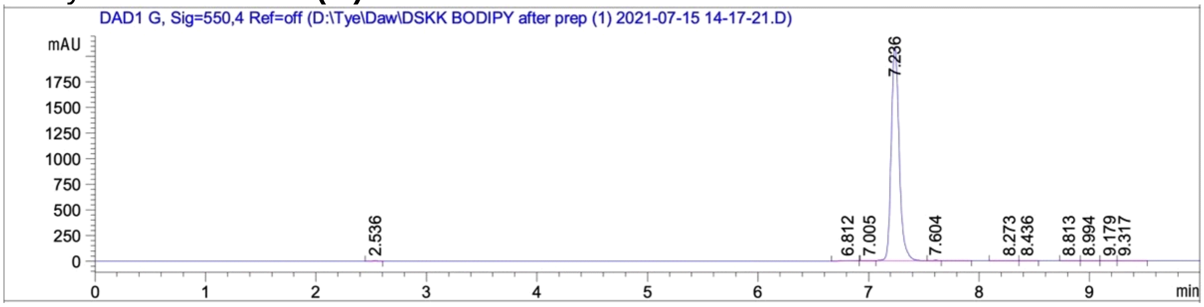

Analytical HPLC of **5b(iii)** at 550 nm detection

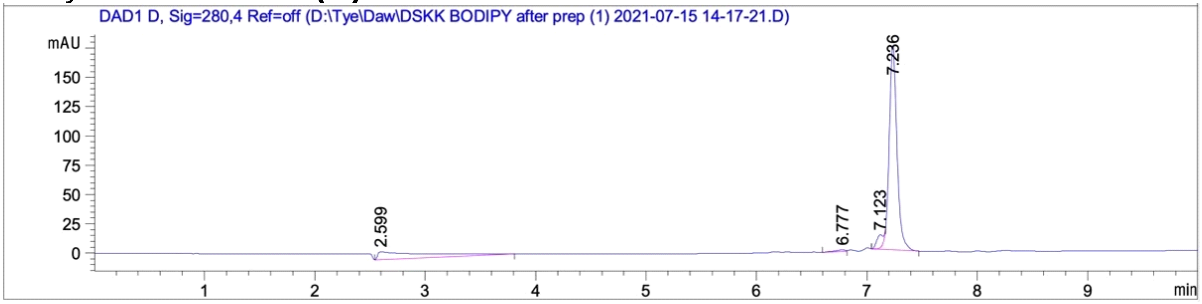

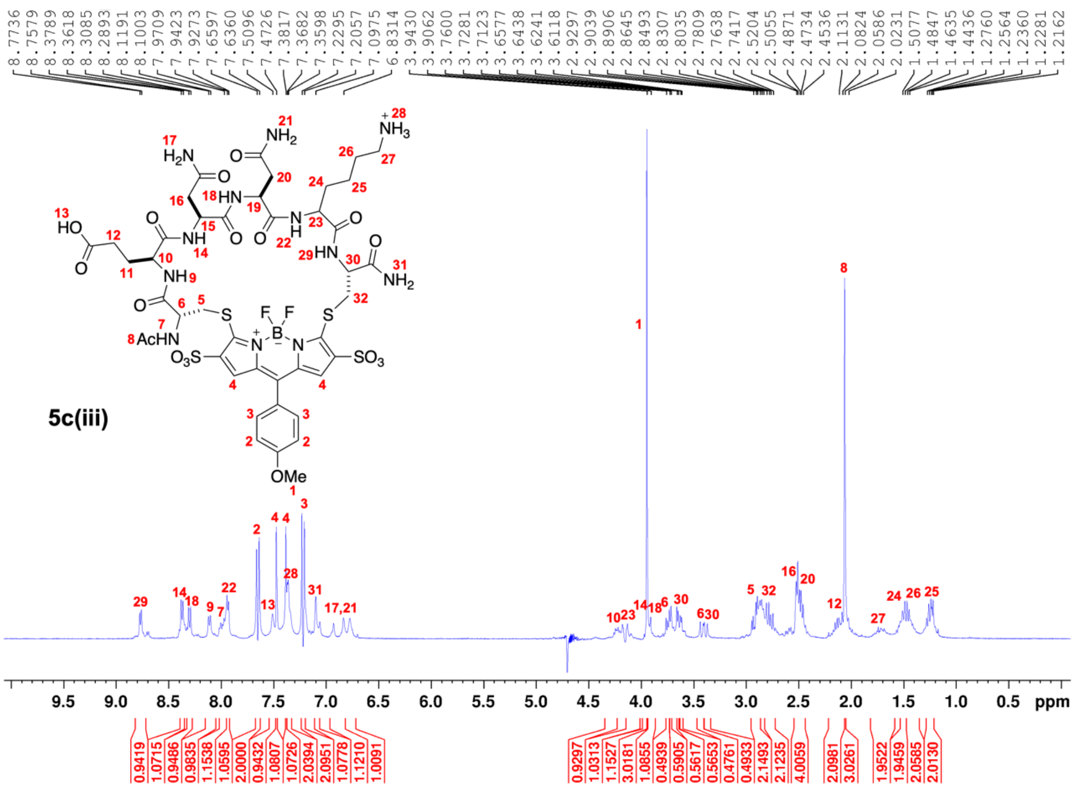

^{1}H NMR of 5c(iii)

5c(iii)

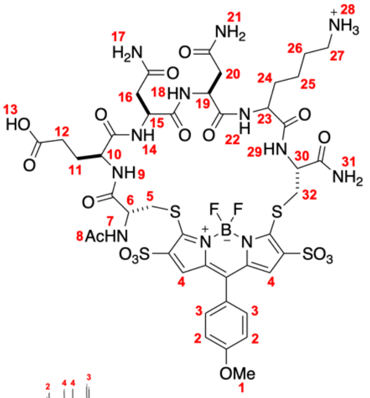

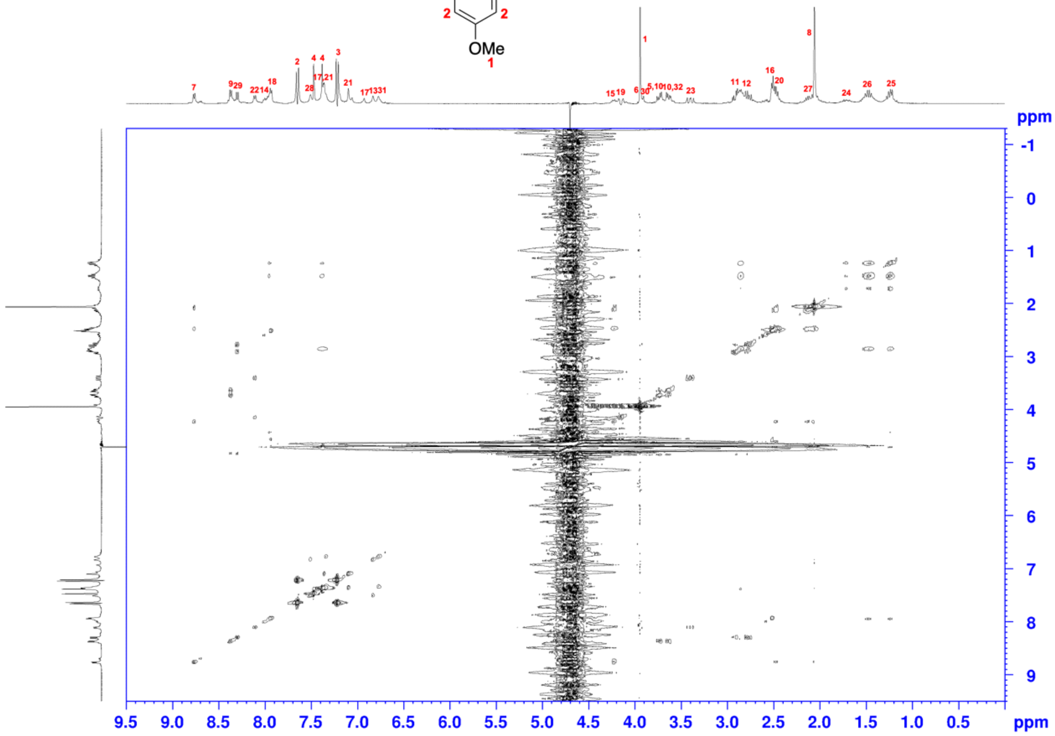

TOCSY of 5c(iii)

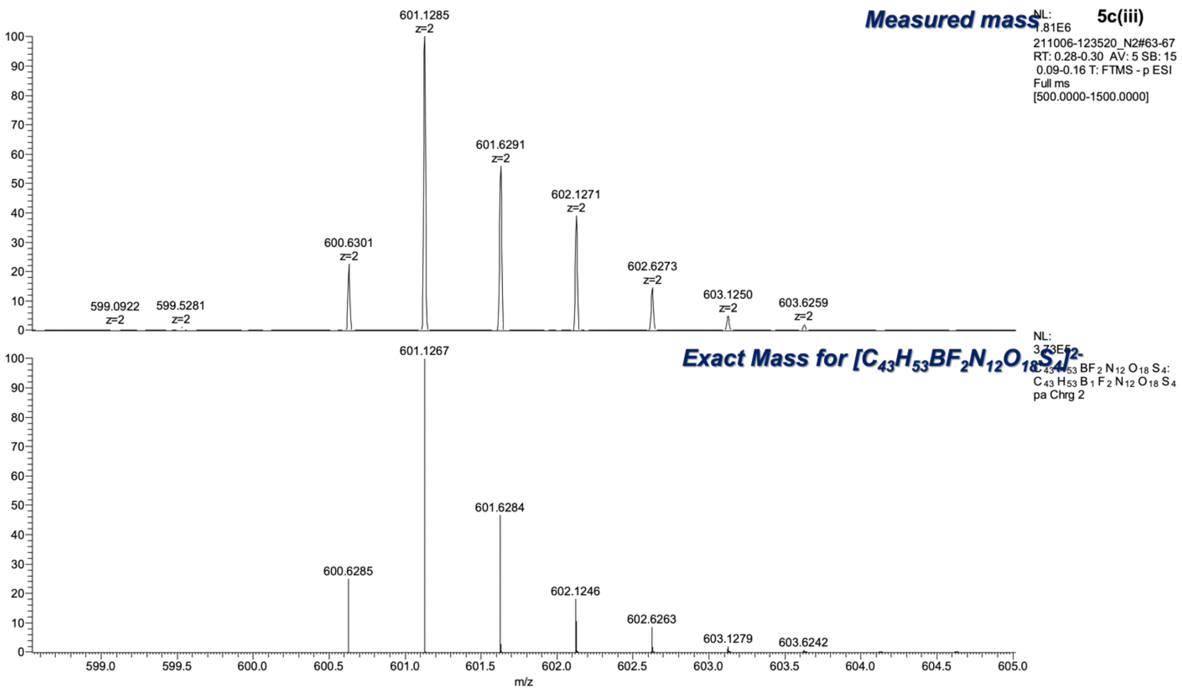

HRMS of 5c(iii)

Analytical HPLC of **5c(iii)** at 280 nm detection

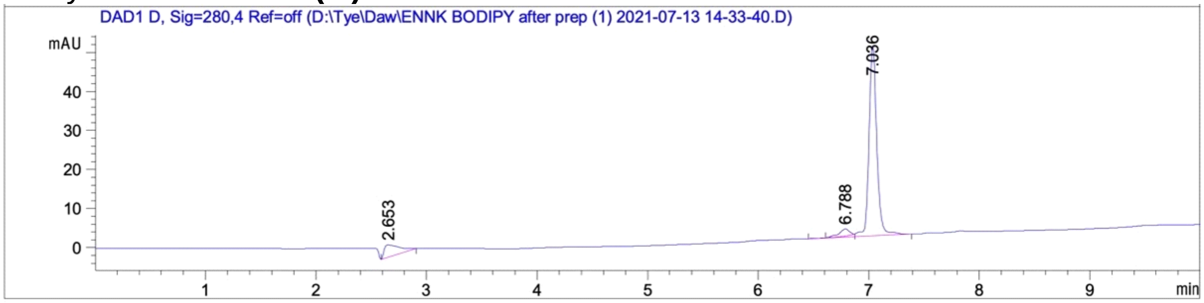

Analytical HPLC of **5c(iii)** at 550 nm detection

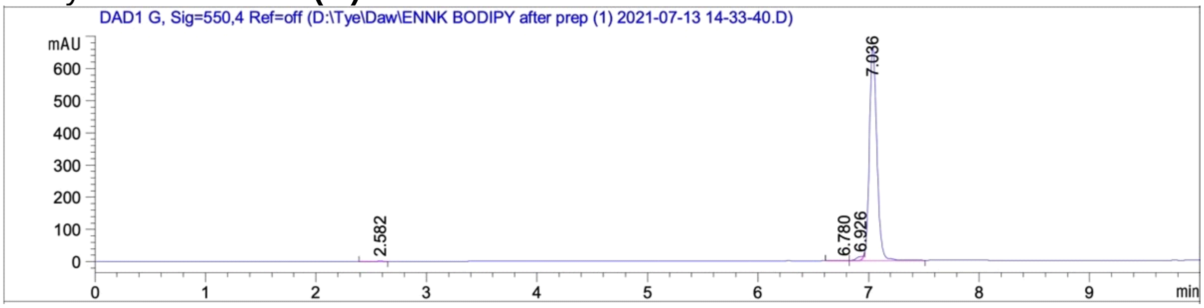

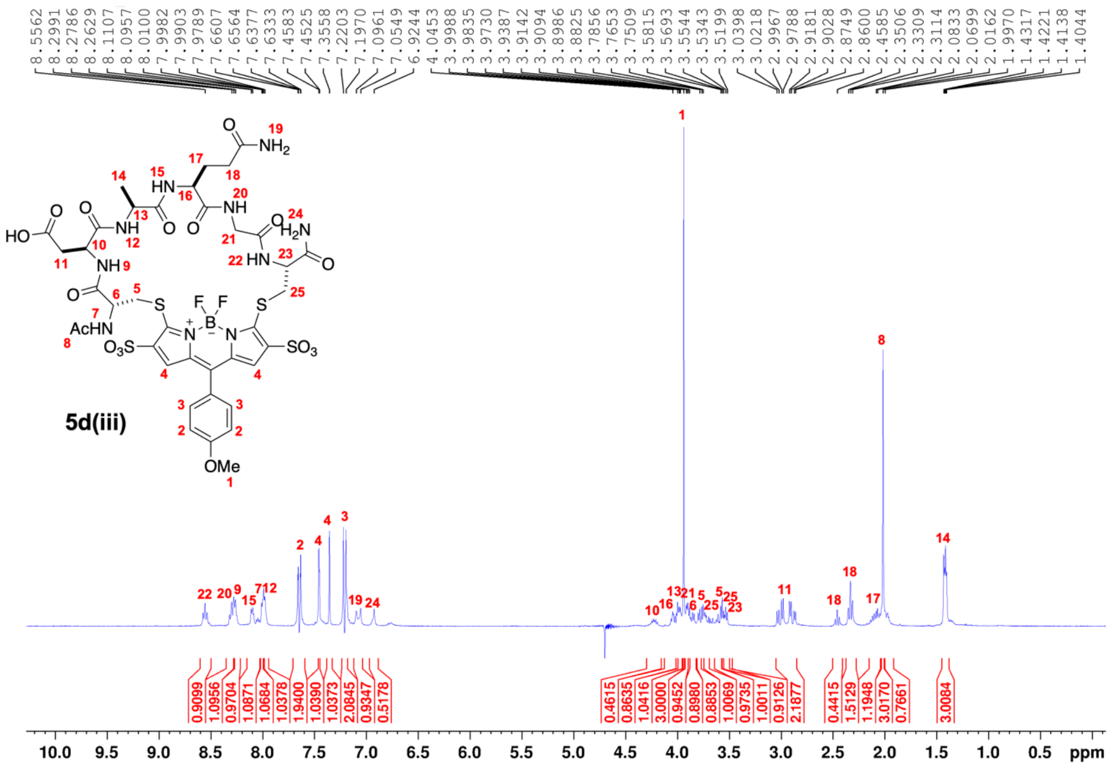

^{1}H NMR of 5d(iii)

5d(iii)

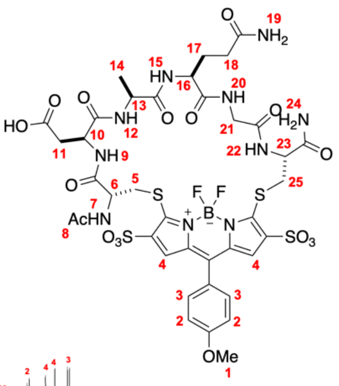

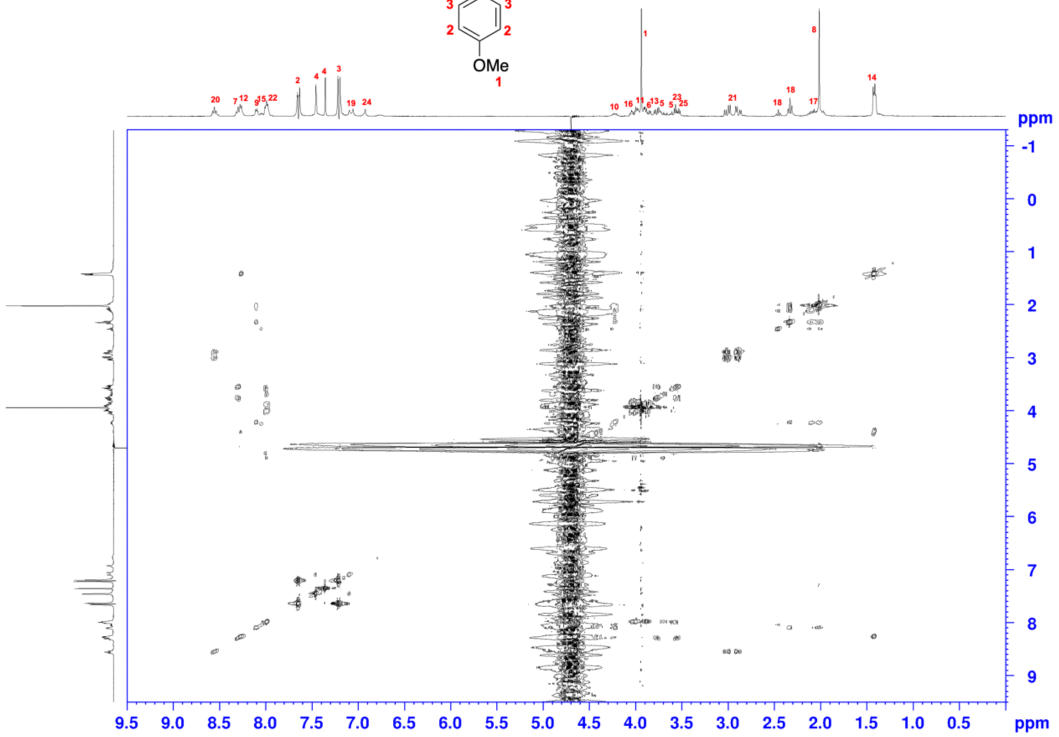

TOCSY of 5d(iii)

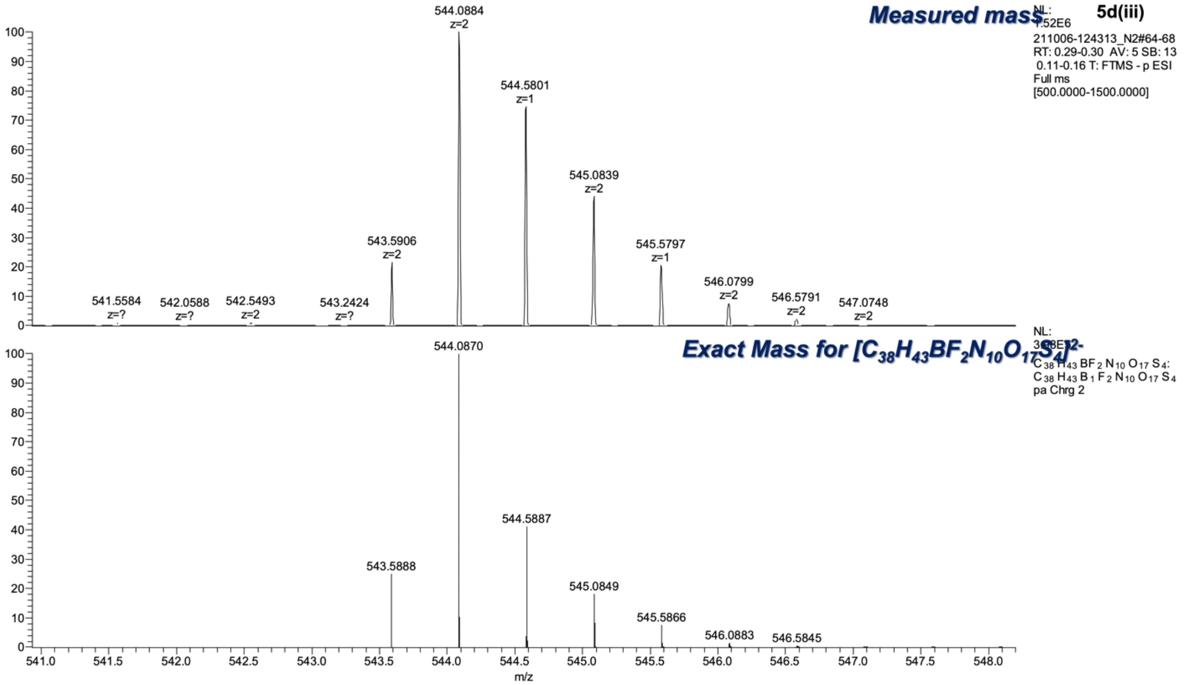

HRMS of 5d(iii)

Analytical HPLC of **5d(iii)** at 280 nm detection

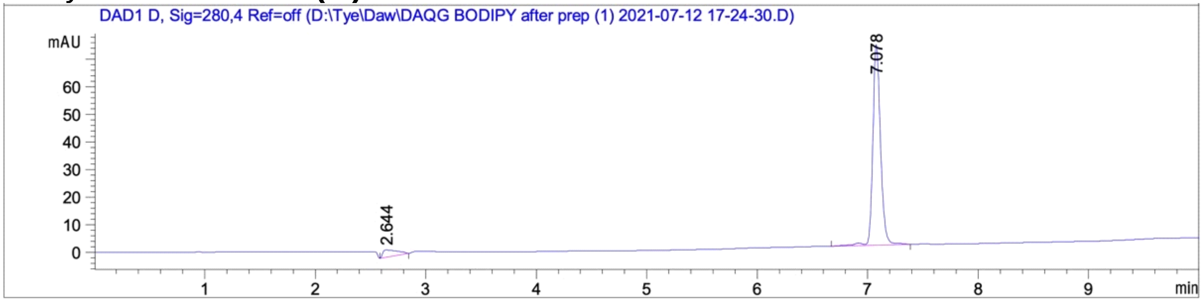

Analytical HPLC of **5d(iii)** at 550 nm detection

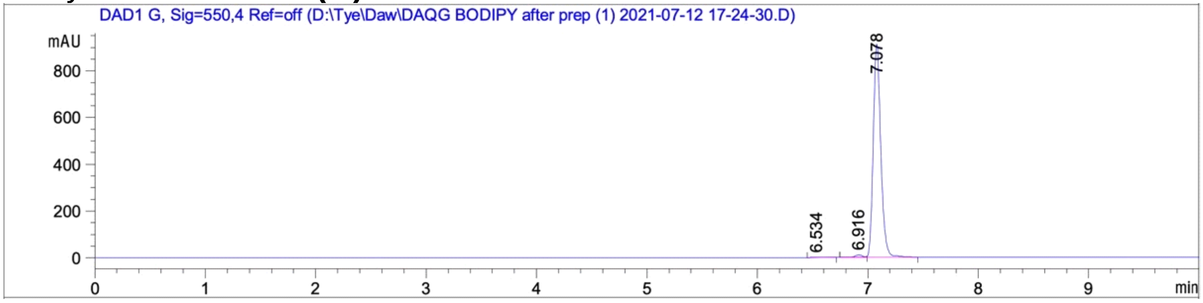

Summary of HRMS and % purity of compounds

Compound ID	Sequence C-(X^1,X^2,X^3,X^4)-C	Exact mass **$[M]^{2-}$** (calc'd)	m/z found By HRMS **$[M]^{2-}$**	% Purity by HPLC (at 280 nm)
5a(i)	-DIKG-	565.1287	565.1305	98

5b(i)	-DMSG-	553.5754	553.5773	91
5c(i)	-DIRG-	579.1318	579.1332	100
5d(i)	-DLRG-	579.1318	579.1333	91
5a(ii)	-INNS-	572.6161	572.6172	93
5b(ii)	-VSKG-	544.1234	544.1248	94
5c(ii)	-TQNS-	573.6074	573.6056	96
5c(ii)m	-TGNS-	538.0870	538.0884	97
5d(ii)	-AGGS-	494.5710	494.5721	92
5a(iii)	-DGKQ-	572.6160	572.6175	99
5a(iii)m	-DEKQ-	608.6265	608.6286	97
5b(iii)	-DSKK-	587.6394	587.6413	94
5c(iii)	-ENNK-	601.1267	601.1285	98
5d(iii)	-DAQG-	544.0870	544.0884	99

Absorption and Fluorescence Spectra

UV/vis absorption spectra and fluorescence were recorded on a Cary 100 Bio UV-Visible Spectrophotometer and a Cary Eclipse Fluorescence Spectrophotometer using a quartz cuvette with a 1 cm path length. In both experiments, stock solutions of compounds were prepared in DMSO. 2 μM solutions of compound in DI water were prepared for working solutions. All compounds have a maximum absorption wavelength between 543-550 nm. For fluorescence experiments, the emission spectra were recorded at excitation wavelength of 500 nm and maximum emission wavelength of all compounds are between 572-577 nm.

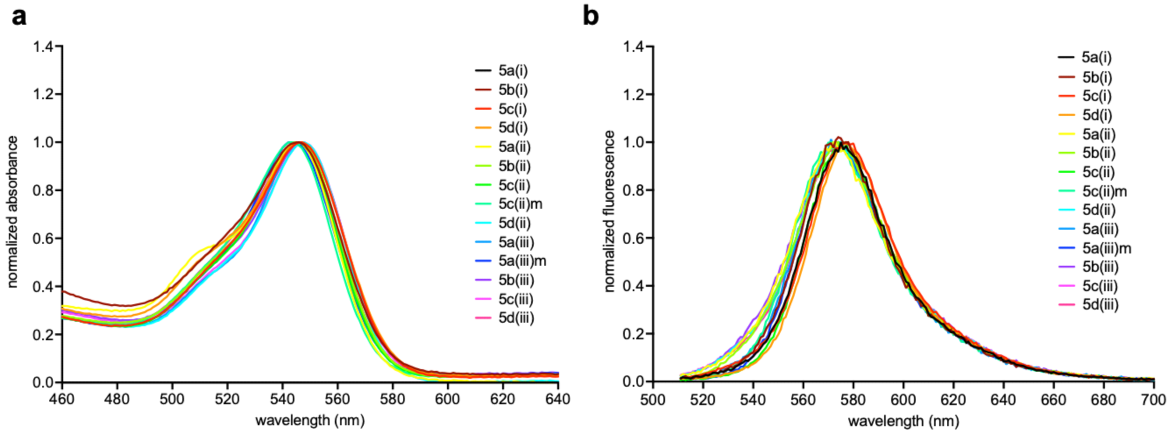

a) UV-vis absorption and b) fluorescent spectra of all compounds (2 μM) excited at 500 nm in DI water.

Cytotoxicity Assays

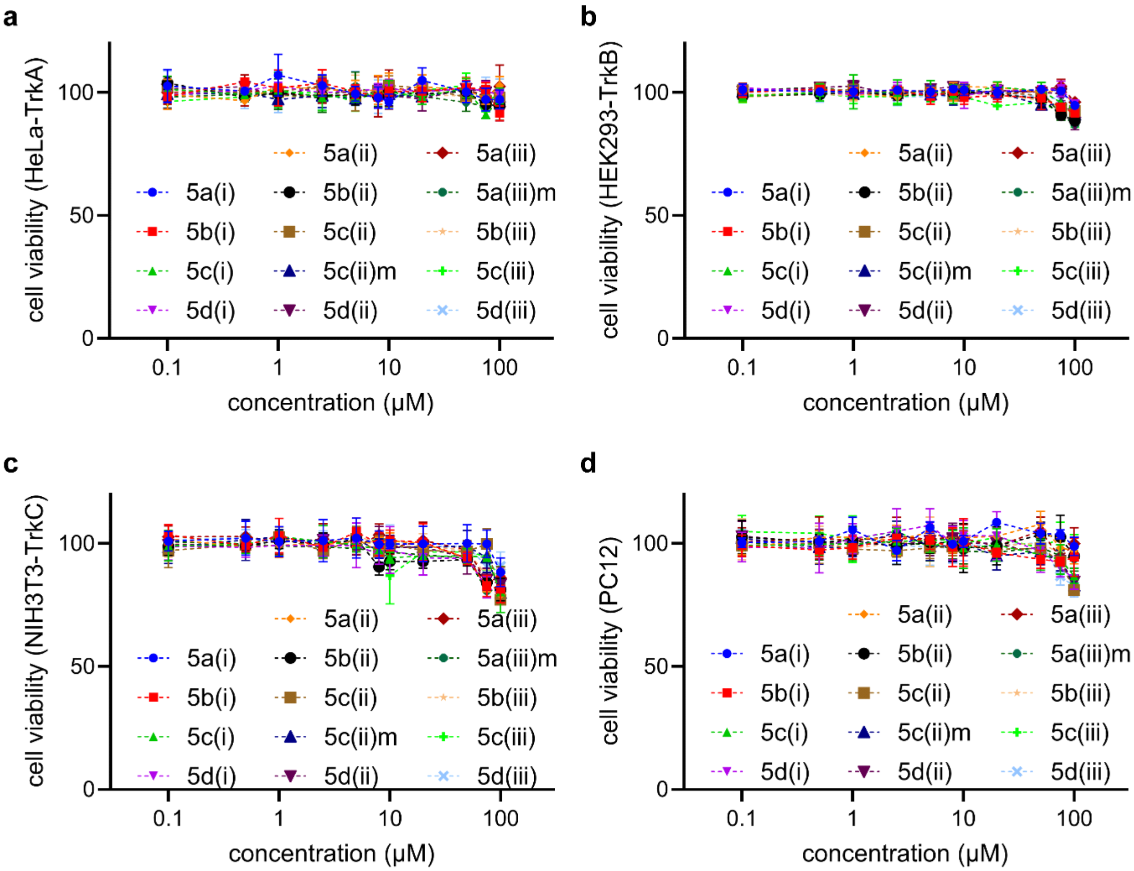

Fig S1 Cytotoxicity via cell viability of all compounds in **a** HeLa-TrkA, **b** HEK293-TrkB, **c** NIH3T3-TrkC, and **d** PC12 cells.
